# Heritability Enrichment of Immunoglobulin G N-Glycosylation in Specific Tissues

**DOI:** 10.3389/fimmu.2021.741705

**Published:** 2021-11-03

**Authors:** Xingang Li, Hao Wang, Yahong Zhu, Weijie Cao, Manshu Song, Youxin Wang, Haifeng Hou, Minglin Lang, Xiuhua Guo, Xuerui Tan, Jingdong J. Han, Wei Wang

**Affiliations:** ^1^ Centre for Precision Health, Edith Cowan University, Joondalup, WA, Australia; ^2^ School of Medical and Health Sciences, Edith Cowan University, Joondalup, WA, Australia; ^3^ Beijing Key Laboratory of Clinical Epidemiology, School of Public Health, Capital Medical University, Beijing, China; ^4^ Beijing Lucidus Bioinformation Technology Co., Ltd., Beijing, China; ^5^ School of Public Health, Shandong First Medical University & Shandong Academy of Medical Sciences, Tai’an, China; ^6^ Chinese Academy of Sciences (CAS) Center for Excellence in Biotic Interactions, College of Life Science, University of Chinese Academy of Sciences, Beijing, China; ^7^ The First Affiliated Hospital, Shantou University Medical College, Shantou, China; ^8^ Peking-Tsinghua Center for Life Sciences, Academy for Advanced Interdisciplinary Studies, Center for Quantitative Biology (CQB), Peking University, Beijing, China

**Keywords:** genome-wide association study, immunoglobulin G, N-glycosylation, single nucleotide polymorphism, transcriptome-wide association study

## Abstract

Genome-wide association studies (GWAS) have identified over 60 genetic loci associated with immunoglobulin G (IgG) N-glycosylation; however, the causal genes and their abundance in relevant tissues are uncertain. Leveraging data from GWAS summary statistics for 8,090 Europeans, and large-scale expression quantitative trait loci (eQTL) data from the genotype-tissue expression of 53 types of tissues (GTEx v7), we derived a linkage disequilibrium score for the specific expression of genes (LDSC-SEG) and conducted a transcriptome-wide association study (TWAS). We identified 55 gene associations whose predicted levels of expression were significantly associated with IgG N-glycosylation in 14 tissues. Three working scenarios, i.e., tissue-specific, pleiotropic, and coassociated, were observed for candidate genetic predisposition affecting IgG N-glycosylation traits. Furthermore, pathway enrichment showed several IgG N-glycosylation-related pathways, such as asparagine N-linked glycosylation, N-glycan biosynthesis and transport to the Golgi and subsequent modification. Through phenome-wide association studies (PheWAS), most genetic variants underlying TWAS hits were found to be correlated with health measures (height, waist-hip ratio, systolic blood pressure) and diseases, such as systemic lupus erythematosus, inflammatory bowel disease, and Parkinson’s disease, which are related to IgG N-glycosylation. Our study provides an atlas of genetic regulatory loci and their target genes within functionally relevant tissues, for further studies on the mechanisms of IgG N-glycosylation and its related diseases.

## Introduction

Glycosylation is one of the most ubiquitous and essential posttranslational modifications (PTM) for extracellular proteins in eukaryotes, with the addition of linear or branched oligosaccharide sidechains called glycans to the backbones of proteins (1). According to the glycans covalently attached to asparagine, threonine, or serine side chains, they are named either “N-linked” or “O-linked” (2). Based on the well-known asparagine (Asn)-X-Serine (Ser)/threonine (Thr) sequon, a given eukaryotic glycoprotein may have one or more N-linked glycosylation (N-glycosylation) sites (3). In terms of the relatively clear functional domains and the highly conserved glycosylation site at the equivalent position of Asn-297 of each heavy chain across mammalian species, immunoglobulin G (IgG) has been regarded as an ideal N-glycoprotein model for researching N-glycosylation (4, 5).

N-Glycan is initially synthesized from a lipid-linked, oligosaccharide moiety (Glc3Man9GlcNAc2-P-P-dol) on the lumen side of the endoplasmic reticulum (ER) and transferred to the nascent polypeptide chains in the ER. N-glycan is then conservatively trimmed to a core moiety (Man5GlcNAc2-Asn) by a series of exoglycosidases in the ER before transfer to the Golgi apparatus for the following optional glycan assembly (6). Assembly of the glycan-extended tree is controlled by multiple exoglycosidases and the Golgi-localized glycosyltransferases, resulting in a wide variety of oligosaccharide structures showing high species specificity (7). At present, almost 200 glycosylation-related genes have been identified in the human genome (summarized in GlycoGene Database (GGDB, https://acgg.asia/ggdb2/) (8), representing approximately 1% of all human genes. However, glycan branching in the Golgi is highly dependent on microenvironment, such as tissue-specific regulation of the expression of glycoenzymes along the Golgi assembly line. Due to the lack of N-glycan profiling data for particular tissues, even to the best-known glycoprotein, human IgG, it remains unclear whether its N-glycosylation is regulated differentially across multiple tissues and how tissue-specific regulation contributes to its diverse N-glycosylation.

GWAS have identified over 60 susceptibility loci associated with the alternative N-glycan peaks (N-GPs) of IgG, which is the qualification and quantification of enzymatically released N-glycans by ultra-performance liquid chromatography (UPLC) after the IgG is isolated from plasma (9–12). Four of the 200 glycogenes (8) are located in these identified GWAS loci, including *FUT6*, *FUT8*, *B4GALT1*, and *MGAT3*, implying their contribution to the alternative IgG N-glycosylation. However, over 90% of identified GWAS hits are difficult to characterize biologically due to the pitfalls of GWAS approach, e.g., very small effect size, within the noncoding region, pleiotropic, and/or noncausative (13). Thus, a large number of functionally relevant genes underpinning these GWAS associations of IgG N-glycosylation remain unidentified.

Furthermore, immune cells, e.g., plasma cells which synthesize and secrete IgG, are highly motile between blood and lymphatic circulation, traveling around the lymphoid nodes and mucosa-associated lymphoid tissues (MALTs), a diffuse lymphoid tissue system found in submucosal parts of the body (e.g., gastrointestinal tract, nasopharynx, thyroid, breast, lung, salivary glands, and skin), throughout the body to reach a site of inflammation (14). On amount of the existence of tissue-specific gene expression (15) and the limitation that only plasma IgG has been investigated in population-based studies for the genetic effect of IgG N-glycosylation, it is still unclear whether or not the N-glycosylation of IgG is regulated differentially among multiple MALTs. Likewise, how the genetic susceptibility of quantitative trait loci (QTL) identified by GWAS affects IgG N-glycosylation through the tissue-specific regulation of gene expression remains unknown. Recent genomic/transcriptomic-based statistical approaches (16, 17) may help to shed light on the complicated mechanisms of IgG N-glycan biosynthesis, especially concerning tissue-specific regulation.

**Figure 1:**
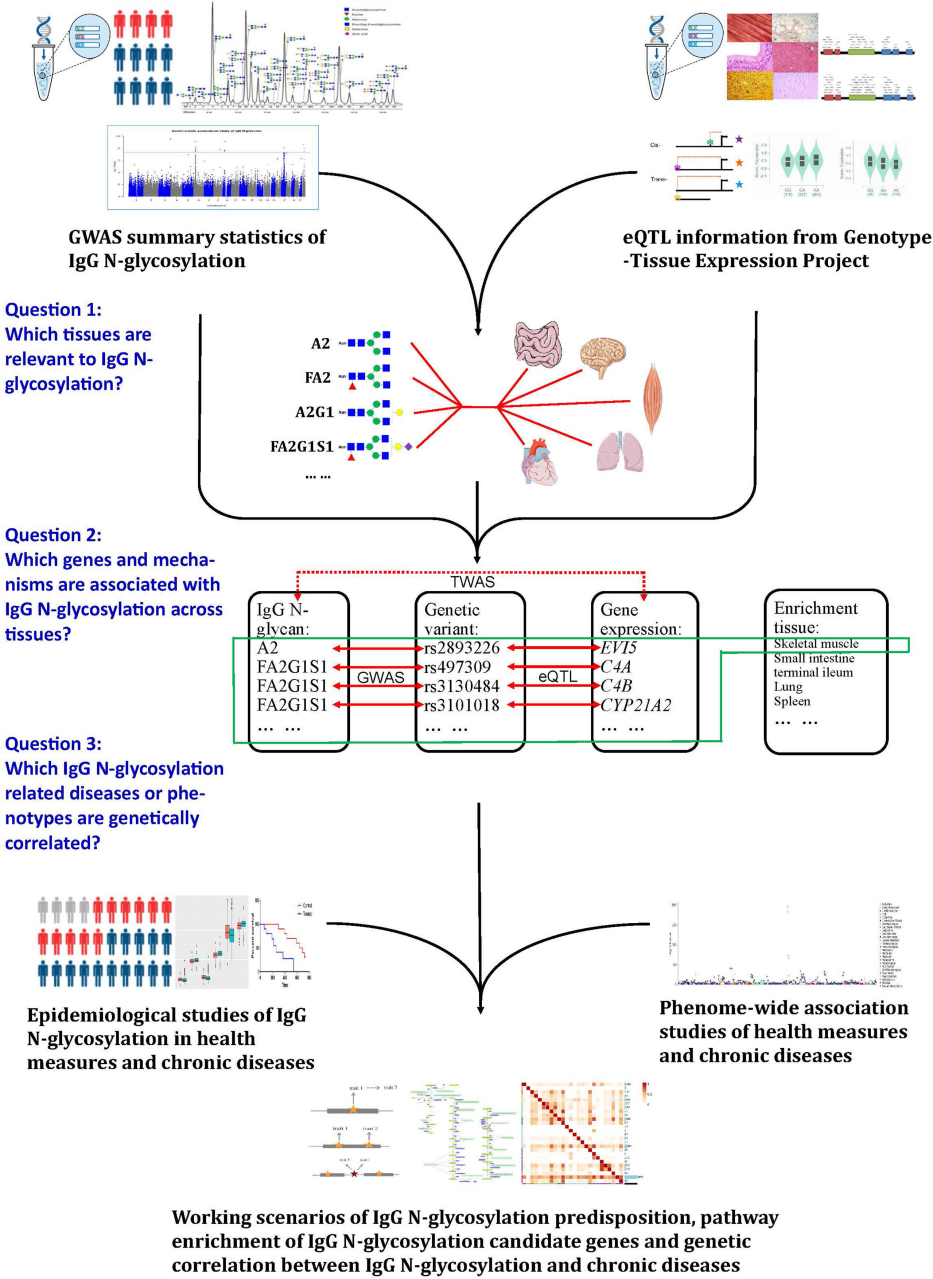
A schematic analysis plan in the study. Leveraging IgG N-glycosylation genome-wide association studies (GWAS), summary statistics, and gene expression datasets: (1) to filtrate the tissues enriched in IgG N-glycosylation signal, (2) to identify the genes most significantly associated with IgG N-glycosylation features, and (3) to investigate the diseases or phenotypes involved with IgG N-glycosylation.

In the present study, to identify genetically regulated genes associated with IgG N-glycosylation traits across the multitude of tissues, we leveraged the data of GWAS on IgG N-glycosylation from 8,090 participants of European ancestry (11) and the data from a large-scale expression QTL (eQTL) study, i.e., Genotype-Tissue Expression of 53 types of tissue (GTEx v7) (18). We first conducted a linkage disequilibrium scores for the specific expression of genes (LDSC-SEG) (16) to filtrate which tissues are most likely to be enriched with each specific IgG N-GPs having significant GWAS results (20 out of 23 GPs have significant GWAS hits). To avoid the tissue bias in following transcriptome-wide association study (TWAS), we selected corresponding tissue types in expression panels of functional summary-based imputation (FUSION), matched with the LDSC-SEG screening tissues and the LDSC-SEG significant IgG N-GPs for TWAS analyses (17). To investigate whether the significance of TWAS hits resulted from the regulation of gene expression or a genetically associated effect, we conducted joint and conditional analyses on each TWAS hit. We next explored the biological pathways of candidate genes from TWAS and accessed the network of corresponding gene sets by protein-protein interaction (PPI) analysis within IgG N-glycosylation-related tissues. At last, we retrieved the single nucleotide polymorphisms (SNPs) underlying the TWAS hits in phenome databases to discover the complex traits and diseases sharing genetic susceptibility with IgG N-glycosylation. A schematic analysis plan of our study can be found in **Figure 1**.

The study aimed to characterize the genetic predispositions of IgG N-glycosylation in their enriched tissues, thus extending our understanding of the genetic regulation of IgG N-glycosylation-related gene expression in the corresponding tissues and to determine their association with susceptibility genes for IgG N-glycosylation-related traits and diseases.

## Materials and Methods

### GWAS Dataset of IgG N-Glycosylation

The IgG N-glycosylation GWAS summary statistics used in LDSC-SEG and TWAS were acquired from the NHGRI-EBI GWAS Catalog (19) for study GCST009860, the most recent large-scale GWAS meta-analysis (8,090 participants of European ancestry) (11). The GWAS summary statistics were downloaded from https://datashare.is.ed.ac.uk/handle/10283/3238/ on May 27, 2020. Information about IgG N-glycosylation peaks (IGPs) is given in **Supplementary Table 1** by both Edinburgh code and Zagreb code. Additional details on the quantification of IGPs and genotyping can be found in previous studies (9, 10).

To be consistent with our previous studies, we used the Zagreb code (GP1–GP24) for naming each GP profiled by UPLC in the IgG N-glycome. Detailed naming and compositional information of GPs were given in a previous report (9, 20) and are listed in **Supplementary Table 2**. Meanwhile, being consistent with public data from GTEx, we used the same tissue names as those in GTEx. Of the total 24 GPs, significant GWAS summary statistics of 20 GPs were investigated by LDSC-SEG. Whereas the remaining four GPs (GP1, GP3, GP5, and GP21) were excluded due to a lack of GWAS statistical significance.

### Transcriptomic Dataset for Linkage Disequilibrium Score Regression of Specifically Expressed Genes in LDSC-SEG

Integrating GWAS with large-scale functional genomic data has been proposed as an effective approach to characterize the functional effects of associated genetic variants, especially for cross-tissue study (21). LDSC-SEG provides a solid bioinformatics tool for discerning which tissues or cell types are most relevant to a particular disease or health phenotype (16). Therefore, LDSC-SEG is able to perform the tissue enrichment analyses for a specific phenotype by integrating stratified linkage disequilibrium score regression from GWAS summary statistics with tissue-specifically expressed gene sets in a huge volume of gene expression data (22).

Data for LDSD-SEG analysis were prepared as described in a previous study (https://alkesgroup.broadinstitute.org/LDSCORE/) (16). A total of 53 multitissue samples from GTEx v7 were included in this LDSC-SEG analysis (https://github.com/bulik/ldsc) (18).

### Transcriptomic Panels for TWAS in FUSION

TWAS (23) combine genetically predicted gene expression levels with GWAS results on a specific phenotype, to discover genes whose *cis*-regulated expressions are associated with that phenotype (17, 24–27). The above approach has been successfully performed on pathogenesis studies of neurodegenerative disorder (Parkinson’s disease) (28), psychiatric disorders (schizophrenia, attention deficit hyperactivity, autism spectrum, and bipolar disorder) (29–31), and also cancer studies (pancreatic, breast, prostate, and ovarian cancers) (32–36).

Through TWAS analysis, the relationships between SNPs and gene expression levels were first obtained to build-up reference panels composed of predictive models. These models were then used to predict trait-associated gene expressions, *via* the statistically significant SNPs from the GWAS summary statistics of an interesting trait based on a large independent cohort (17, 25).

The FUSION method was performed to estimate heritability, build predictive models, and identify transcriptome-wide associations. By FUSION, the associations between the IgG N-GPs and the expression levels of candidate genes in corresponding tissues were identified *via* the coassociated genetic variants (as SNPs) which are identified as statistically significant in GWAS (as QTLs) and also in GTEx (as eQTLs).

Transcriptomic imputation (TI) in FUSION method was conducted using eQTL reference panels which were derived from tissue-specific gene expression coupled with genotypic data. In the current study, 27 tissues were identified as relevant with IgG N-glycosylation by LDSC-SEG, while three tissues were unavailable in FUSION. Hence, 24 tissue panels were used as TI reference panels, while the 1,000 Genomes v3 LD panel (http://ftp.1000genomes.ebi.ac.uk/vol1/ftp/release/20130502/) was hired as an LD reference. A Bonferroni-corrected study-wise threshold was calculated by *p* = 0.05/number of genes in each panel (**Supplementary Table 2**). As previous GWAS on IgG N-GPs reported that immune tissues are relevant to IgG N-glycosylation (10, 11), we employed three immune tissues, i.e., spleen, whole blood, and Epstein-Barr virus (EBV)-transformed lymphocytes as a complementary strategy of expression panel selection in FUSION, along with 20 GWAS-significant IgG N-GPs to conduct a parallel TWAS analysis.

### Identification of IgG N-Glycosylation Relevant Tissues by LDSC-SEG

The linkage disequilibrium score for the specific expression of genes (LDSC-SEG) is a computational approach to identify phenotype-relevant tissues using stratified LD score regression (https://alkesgroup.broadinstitute.org/LDSCORE/) (16). In a given tissue, if there is an enrichment of the highest specific expression in the regions surrounding the heritability of a disease/phenotype, it will support the likely correlation between this disease/phenotype and this tissue (16). By this approach, we investigated 53 tissues from the GTEx project (http://gtexportal.org/) (18), to designate the tissues relevant to IgG N-glycosylation features. All procedures follow the tutorial in Github (https://github.com/bulik/ldsc).

### Performing TWAS on IgG N-Glycosylation GWAS Dataset

TWAS has been proposed as a robust tool to integrate GWAS summary statistics, *cis*-SNP-expression effect sizes, and LD reference panels to evaluate the association between the *cis*-genetic element of expression and disease/phenotype (28, 30, 31). Thus, using the colocalized SNPs between GWAS statistics and eQTL data as linkers, TWAS is able to identify the candidate genes for the potential mechanism underlying the variant-disease/phenotype associations, which are challenging for GWAS approach.

In the present study, we conducted FUSION tool (http://gusevlab.org/projects/fusion/) (17) for each transcriptome reference panel designated by an LDSC-SEG approach. Briefly, to estimate the heritability of each gene expression, we first conducted a robust version of Genome-wide Complex Trait Analysis-Genome-based restricted maximum likelihood (GCTA-GREML). This step generated the heritability estimates of expression for each gene with the *p* of the likelihood ratio test.

FUSION has created five different models to calculate the predictive weights of expression or intron usage: best linear unbiased prediction (blup), Bayesian sparse linear mixed model (bslmm), LASSO regression (lasso), Elastic Net (enet), and top SNPs (topl). After weighting, the cross-validation for each of the desired models was performed. The model gaining the best cross-validation prediction accuracy was chosen and the corresponding predictive expression or intron usage was correlated to IgG N-glycosylation GWAS summary statistics to conduct TWAS and filtrate significant associations. The significance for heritability estimates of the genes or intron usage at a Bonferroni-corrected *p* < 0.05 were reported as TWAS hits. Accounting for more suggestive information on gene coexpression, we also applied an FDR of 5% within each expression reference panel to obtain a bigger risk gene set for pathway analysis.

### Joint and Conditional Testing GWAS Signal Analysis

Using the postprocess module in FUSION (http://gusevlab.org/projects/fusion/), joint and conditional testing methods were performed to determine the contribution of gene expression association in each significant TWAS hit. After the weight of gene expression was removed, the residual TWAS signal was recalculated and evaluated with genome-wide Bonferroni correction. The testing region was defined by the transcribed region of the genes. In each testing, every association of GWAS was conditioned upon the joint gene model by one SNP.

### Colocalization Analyses and Functional Annotation

Using *coloc* approach (37), the colocalization analyses were conducted to strengthen the detection of candidate genes at IgG N-glycosylation GWAS loci by hunting the evidence of shared causal variants between functional eQTL traits and GWAS traits. The colocalization test converts correlation statistics to effect size based on the sample size of the study for a given function, i.e., gene expression. Then, under the assumption that the standard error approximation is inversely proportional to the square root of the sample size, the approximate colocalization effect size is calculated. The statistics of posterior probability (PP) were presented for the five hypotheses (PP.H0: unrelated; PP.H1: only functionally relevant; PP.H2: only GWAS relevant; PP.H3: independent function/GWAS related; and PP.H4: colocalized function/GWAS related). The current study mainly concerned the PP.H4, which represents the posterior probability that GWAS significant signal and eQTL locus are the same locus, ranging from 0 to 1, where 0 means 0% probability and 1 means 100% probability. Colocalization is declared if the posterior PP.H4 for the model with a shared causal variant exceeded 0.750.

The online tool, *HaploReg* v4.1 (https://pubs.broadinstitute.org/mammals/haploreg/haploreg.php) (38) was used to annotate the potential functions of the best eQTLs which were identified by FUSION and *coloc*, for dbSNP function, promoter and enhancer activity regions, DNAse, protein-binding regions, and transcription factor-binding motifs.

### Gene-Set Analyses

Agnostic analyses were performed in STRING portal (http://string-db.org/) (39), according to Gene Ontology (GO), Kyoto Encyclopedia of Genes and Genomes (KEGG), and Reactome databases to identify pathways relevant to IgG N-glycosylation. Gene clustering was conducted using the GeneNetwork v2.0 (https://genenetwork.nl) (40), which was based on RNA sequencing database (*n* = 31,499).

### Phenome-Wide Association Studies and Genetic Correlation Investigation

To identify more diseases/phenotypes associated with the most significant eQTL of each TWAS gene, we performed a phenome-wide association study (pheWAS) on each leading SNP, by leveraging the public data in the GWASAtlas (https://atlas.ctglab.nl) (41). Based on *p-*values, the top 5 phenotypes (excluding IgG N-glycosylation and repeated phenotypes) were presented. The genetic correlation between the diseases/phenotypes identified by pheWAS was determined by LDSC, using available data in the GWASAtlas. We utilized the most recent GWAS data (i.e., until 2020) for analysis. A Bonferroni correction was utilized to adjust the significance threshold with the number of tested traits.

## Results

### Identification of IgG N-Glycosylation-Relevant Tissues by Heritability Enrichment of Expressed Genes

To better comprehend how peripheric tissues affect IgG N-GPs and to avoid tissue bias in following TWAS, we conducted a tissue enrichment analysis using the LDSC-SEG method to leverage the newest IgG N-glycosylation GWAS summary statistics (11) and eQTL data from the GTEx consortium (v7) (15, 18). The study comprised 48 tissue types (*n* = 80 to 491, **Supplementary Table 1**) with a 5% false discovery rate (FDR) threshold.

**Figure 2:**
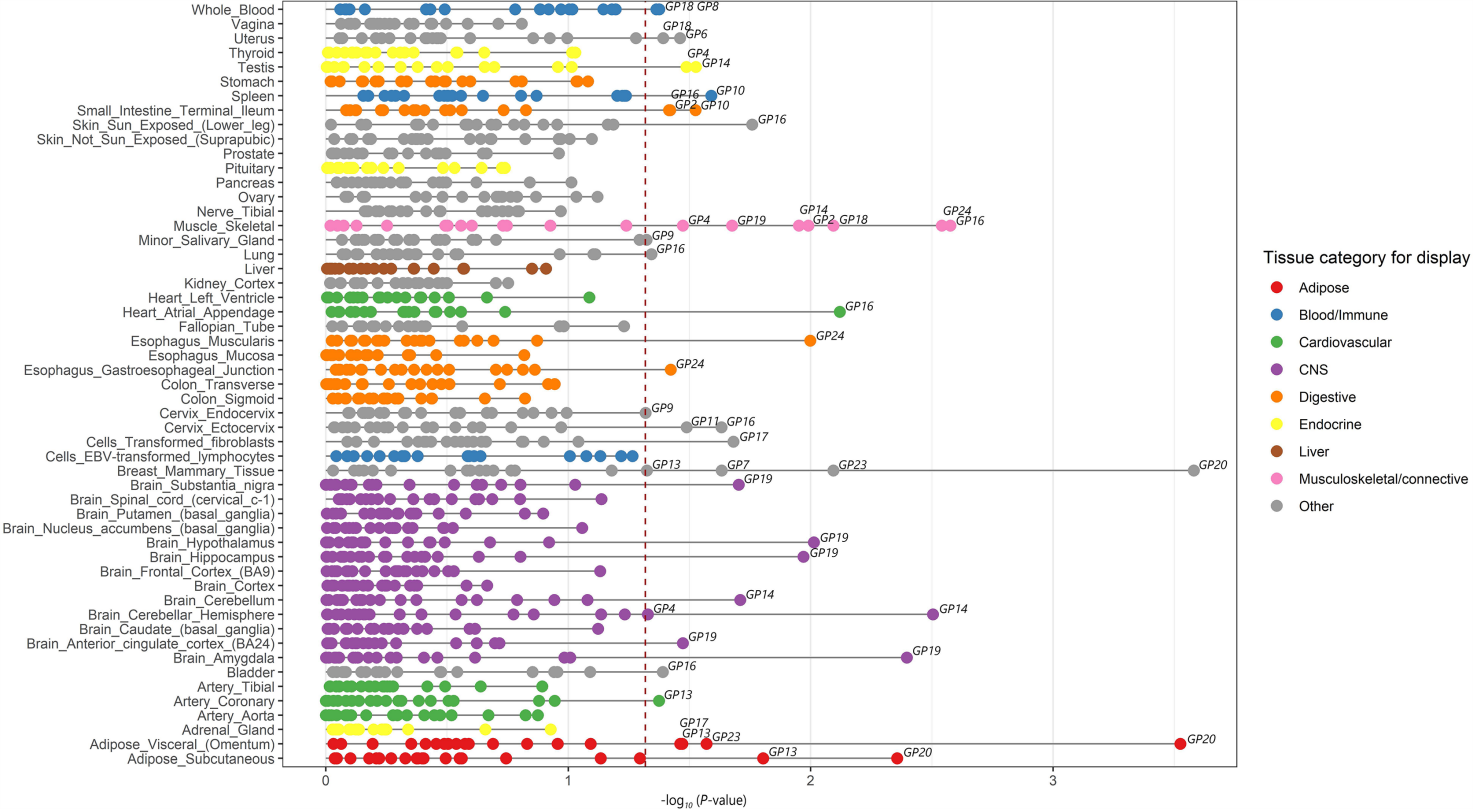
Enrichment of tissues in IgG N-glycosylation. Significantly enriched tissues of IgG N-glycosylation were identified by linkage disequilibrium score regression in a specifically expressed gene (LDSC-SEG) approach. A total of 53 types of tissue obtained from the Genotype-Tissue expression project (GTEx v7) are grouped into nine domains with different colors. Twenty IgG N-glycan peaks (GPs) with significant GWAS results are enriched in all 53 types of tissue. Twenty-seven tissues are highly enriched for IgG N-glycosylation GWAS signals among 53 types of tissues, with a FDR significant at 5% (red dotted line).

Seventeen GPs were enriched in 27 of the 53 types of tissue in the gene expression panels of GTEx v7 at a 5% FDR threshold (-log_10_ *p* > 1.32) (**Supplementary Table 3**, **Figure 2**; **Supplementary Figure 1**). These 17 GPs and 27 relevant tissues were therefore chosen as the primary strategy for gene expression panel selection in the following TWAS analysis.

For the three immune tissues selected as the complementary strategy, only three GPs including FA2[6]G1 glycan (GP8), FA2[6]BG1 glycan (GP10), and FA2G2S1 glycan (GP18) were enriched in spleen and whole blood, but no enrichment was found in EBV-transformed lymphocytes (**Supplementary Table 3**). These results indicated that genetic effects of IgG N-GPs on the regulation of gene expression are tissue selective, and more likely to be enriched not only in the tissues of immune organs but also broadly in multiple MALTs across peripheral organs, such as skin, lung, breast mammary tissue, small intestine, esophagus muscularis, testis, and uterus.

### Constructing a List of Tissue-Dependent Associations Between Genes and IgG N-Glycan Traits

**Figure 3:**
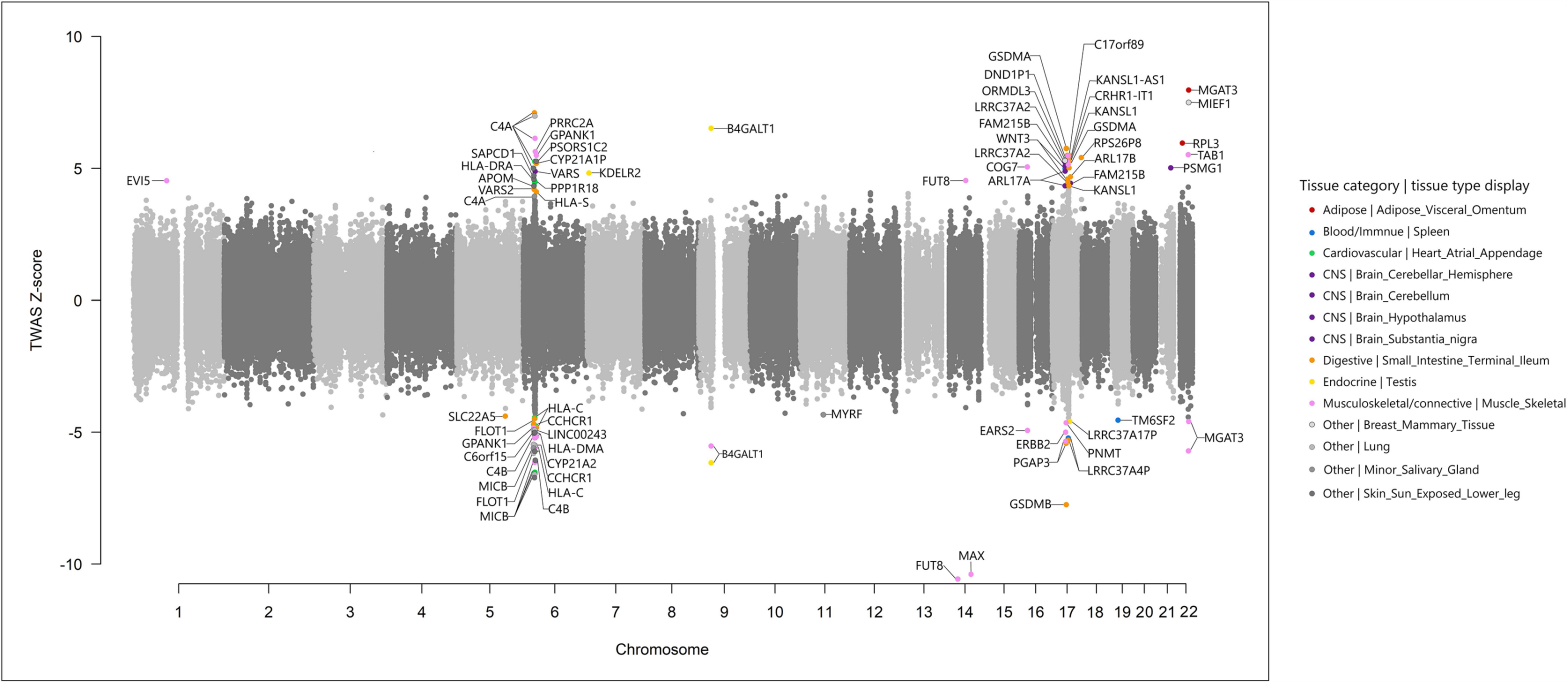
Miami plot of the transcriptome-wide association study for IgG N-glycosylation (*n* = 8,090) using gene expression models in 24 tissues. Each point refers to a single gene tested, with the physical position plotted on the *x*-axis, and a *Z*-score of association between gene expression and IgG N-glycan peaks plotted on the *y*-axis. Bonferroni-adjusted significant genes within corresponding transcriptome panels are labeled in multiple colors according to different tissue categories and tissue types.

To identify the *cis*-regulated genes associated with IgG N-GPs within their functionally relevant tissues, we conducted a TWAS analysis using the FUSION method (see URLs in the *Data Availability Statement*), in terms of the same IgG N-glycosylation GWAS summary statistics (11) and the reference transcriptome panels derived from GTEx v7 (15, 18). In total, 116,076 features of tissue-specific gene expression were tested (see details in **Supplementary Table 1**). Based on the primary strategy of expression panel selection by LDSC-SEG (24 tissue reference panels out of 27 tissue types enriched by LESC-SEG are available in GTEx v7), 90 sets of IgG N-GP/SNP/gene expression association reached the threshold of Bonferroni-corrected significance within each tissue reference panel. After gene annotation by querying in the Metascape portal (42) (see URLs in the *Data Availability Statement*), 55 genes were confirmed as TWAS results, being significantly associated with 11 IgG N-GPs across 14 tissues (**Figure 3** and **Table 1**).

**Table 1:** Significant TWAS genes for IgG N-glycosylation original.

Tissue types in GTEx	Cytogenetic band	TWAS identified gene	IgG N-glycosylation feature	Lead QTL	Best eQTL	TWAS *Z*-score	TWAS *p*-value	COLOC *PP.H4*
Muscle_Skeletal	1p22.1	*EVI5*	GP2	rs11800409	rs2893226^*^	4.53	5.93E−06	0.193
	6p21.33^a^	*C4A*	GP16	rs3130923	rs497309^†^	5.61	2.05E−08	0.075
	6p21.33^a^	*C4B*	GP16	rs1800629	rs3130484^†^	−5.21	1.90E−07	0.936
	6p21.33^a^	*CYP21A2*	GP16	rs1800629	rs3101018^†^	−5.15	2.59E−07	0.946
	6p21.33^a^	*GPANK1*	GP16	rs3130923	rs3130484^†^	5.50	3.87E−08	0.682
	6p21.33^a^	*GPANK1*	GP19	rs2523591	rs3130484^†^	−5.03	4.81E−07	0.645
	6p21.33^a^	*HLA-C^b^*	GP16	rs2516408	rs2523578^†^	−5.18	2.26E−07	0.000
	6p21.33^a^	*MICB*	GP16	rs3130923	rs2516408^†^	−6.13	9.04E−10	0.993
	6p21.33^a^	*PRRC2A*	GP16	rs3130923	rs2515919^*^	5.53	3.23E−08	0.230
	9p21.1^a^	*B4GALT1^b^*	GP18	rs10813951	rs1411609^*^	−5.53	3.23E−08	0.000
	14q23.3^a^	*FUT8^b^*	GP2	rs11847263	rs761830^§^	−11.70	1.34E−31	0.098
	14q23.3^a^	*FUT8^b^*	GP14	rs11158593	rs761830^§^	4.52	6.22E−06	0.908
	14q23.3^a^	*MAX*	GP2	rs11847263	rs1953230^*^	−11.34	8.37E−30	0.532
	16p12.2^a^	*COG7^b^*	GP18	rs250555	rs250583^*^	5.05	4.46E−07	0.721
	16p12.2	*EARS2*	GP18	rs250555	rs4967958^*^	−4.93	8.11E−07	0.447
	17q12	*ERBB2*	GP2	rs907091	rs2102928^*^	−5.00	5.76E−07	0.103
	17q12	*PGAP3*	GP2	rs907091	rs907089^*^	−5.36	8.14E−08	0.000
	17q12	*PNMT*	GP2	rs907091	rs2271308^*^	−4.62	3.90E−06	0.009
	17q21.1	*GSDMA*	GP2	rs907091	rs8065126^*^	5.76	8.62E−09	0.000
	17q21.31	*KANSL1*	GP14	rs169201	rs169201^†^	5.02	5.22E−07	0.987
	17q25.3^a^	*C17orf89^b^*	GP18	rs2659007	rs883884^*^	5.40	6.52E−08	0.050
	22q13.1^a^	*MGAT3^b^*	GP19	rs5757678	rs1005522^§^	−5.71	1.12E−08	0.988
	22q13.1^a^	*MGAT3^b^*	GP24	rs5750830	rs1005522^§^	−4.58	4.55E−06	0.981
	22q13.1^a^	*TAB1^b^*	GP19	rs738289	rs5757650^*^	4.95	7.35E−07	0.543
Small_Intestine_Terminal_Ileum	5q31.1	*SLC22A5*	GP2	rs11746555	rs2073643^*^	−4.41	1.03E−05	0.042
	6p21.33^a^	*C4A*	GP16	rs3130923	rs3101018^†^	7.01	2.46E−12	0.655
	6p21.33^a^	*CCHCR1*	GP16	rs3130923	rs1265087^†^	−4.91	9.04E−07	0.000
	6p21.33^a^	*CYP21A1P*	GP16	rs1800629	rs3101018^†^	5.19	2.08E−07	0.950
	6p21.33^a^	*FLOT1*	GP16	rs3130557	rs3094220^*^	−4.46	8.08E−06	0.731
	6p21.33^a^	*HLA-C^b^*	GP16	rs2516408	rs1265098^*^	−5.62	1.88E−08	0.120
	6p21.33^a^	*HLA-S*	GP16	rs3130923	rs2844623^†^	4.60	4.22E−06	0.068
	6p21.33^a^	*VARS2*	GP16	rs3130557	rs3130557^†^	4.49	7.21E−06	0.954
	17q12	*PGAP3*	GP2	rs907091	rs903502^*^	−5.41	6.38E−08	0.122
	17q21.1	*GSDMA*	GP2	rs907091	rs3859192^*^	4.89	9.88E−07	0.065
	17q21.1	*GSDMB^b^*	GP2	rs907091	rs9303281^*^	−7.76	8.21E−15	0.967
	17q21.31	*ARL17B*	GP2	rs415430	rs17698176^*^	4.59	4.53E−06	0.021
	17q21.31	*DND1P1*	GP10	rs17689471	rs17689471^†^	5.33	1.00E−07	0.990
	17q21.31	*KANSL1*	GP10	rs17689471	rs169201^†^	5.19	2.07E−07	0.922
	17q21.31	*KANSL1-AS1*	GP10	rs17689471	rs17689471^†^	5.29	1.21E−07	0.990
	17q21.31	*LRRC37A2*	GP10	rs7224296	rs169201^†^	5.05	4.48E−07	0.972
	17q21.31	*LRRC37A4P*	GP10	rs17689471	rs17689471^†^	−5.29	1.19E−07	0.972
	17q21.31	*RPS26P8*	GP10	rs17689471	rs17689471^†^	5.32	1.07E−07	0.822
	17q21.31-q21.32^a^	*WNT3^b^*	GP10	rs7224296	rs199438^*^	4.68	2.88E−06	0.373
Skin_Sun_Exposed_Lower_leg	6p21.33^a^	*APOM*	GP16	rs3130923	rs1150755^*^	4.65	3.28E−06	0.021
	6p21.33^a^	*C4A*	GP16	rs3130923	rs1150753^†^	4.99	5.99E−07	0.090
	6p21.33^a^	*C4B*	GP16	rs1800629	rs3101018^†^	−6.11	1.02E−09	0.940
	6p21.33^a^	*C6orf15*	GP16	rs3130923	rs1265093^*^	−5.11	3.25E−07	0.001
	6p21.33^a^	*CYP21A2*	GP16	rs1800629	rs1150753^†^	−5.01	5.33E−07	0.931
	6p21.33^a^	*HLA-C^b^*	GP16	rs2516408	rs2523578^†^	−5.73	9.93E−09	0.000
	6p21.32^a^	*HLA-DRA^b^*	GP16	rs1150752	rs2858867^*^	4.71	2.48E−06	0.146
	6p21.33^a^	*MICB*	GP16	rs3130923	rs2516408^†^	−6.69	2.17E−11	0.993
	6p21.33^a^	*PSORS1C2*	GP16	rs3130923	rs1265099^*^	5.24	1.65E−07	0.027
	6p21.33^a^	*SAPCD1*	GP16	rs3130923	rs1144709^*^	4.75	2.05E−06	0.791
Spleen	17q21.31	*CRHR1-IT1*	GP10	rs17689471	rs17689471^†^	5.20	2.04E−07	0.990
	17q21.31	*DND1P1*	GP10	rs17689471	rs17689918^†^	5.29	1.20E−07	0.989
	17q21.31	*KANSL1-AS1*	GP10	rs17689471	rs17689918^†^	5.31	1.11E−07	0.988
	17q21.31	*LRRC37A4P*	GP10	rs17689471	rs17689918^†^	−5.25	1.55E−07	0.989
	17q21.31-q21.32^a^	*WNT3^b^*	GP10	rs7224296	rs199520^*^	4.58	4.74E−06	0.858
	19p13.11	*TM6SF2*	GP10	rs7257072	rs2916074^*^	−4.55	5.37E−06	0.056
Heart_Atrial_Appendage	6p21.32	*HLA-DMA*	GP16	rs209473	rs2854275^*^	−4.86	1.19E−06	0.214
	6p21.33^a^	*C4A*	GP16	rs3130923	rs497309^†^	5.26	1.42E−07	0.174
	6p21.33^a^	*CCHCR1*	GP16	rs3130923	rs1265087^†^	−4.75	2.08E−06	0.000
	6p21.33^a^	*HLA-C^b^*	GP16	rs2516408	rs1265087^†^	−4.45	8.54E−06	0.098
	6p21.33^a^	*MICB*	GP16	rs3130923	rs2516412^†^	−6.52	7.05E−11	0.992
	6p21.33^a^	*PPP1R18*	GP16	rs3130557	rs3094663^*^	4.67	3.04E−06	0.740
Lung	6p21.33^a^	*C4A*	GP16	rs3130923	rs1150753^†^	6.13	8.59E−10	0.122
	6p21.33^a^	*CCHCR1*	GP16	rs3130923	rs1265087^†^	−5.54	2.95E−08	0.000
	6p21.33^a^	*FLOT1*	GP16	rs3130557	rs3130557^†^	−5.80	6.74E−09	1.000
	6p21.33^a^	*HLA-C^b^*	GP16	rs2516408	rs2844623^†^	−4.76	1.89E−06	0.000
	6p21.33^a^	*LINC00243*	GP16	rs3130557	rs3130557^†^	−5.04	4.57E−07	0.922
	6p21.33^a^	*MICB*	GP16	rs3130923	rs2516412^†^	−5.63	1.83E−08	0.992
Testis	7p22.1	*KDELR2*	GP4	rs17198191	rs17198191^*^	4.82	1.41E−06	0.999
	9p21.1^a^	*B4GALT1^b^*	GP4	rs10813951	rs17247766^§^	6.51	7.69E−11	0.006
	9p21.1^a^	*B4GALT1^b^*	GP14	rs10813951	rs17247766^§^	−6.16	7.48E−10	0.000
	17q21.32	*LRRC37A17P*	GP14	rs415430	rs169201^†^	−4.58	4.73E−06	0.980
Minor_Salivary_Gland	6p21.33^a^	*C4A*	GP9	rs2516408	rs3101018^†^	4.37	1.26E−05	0.231
	6p21.33^a^	*MICB*	GP9	rs2516408	rs2516412^†^	−6.59	4.46E−11	0.939
	11q12.2	*MYRF*	GP9	rs174576	rs449397^*^	−4.35	1.35E−05	0.097
Brain_Cerebellar_Hemisphere	17q21.31	*ARL17A*	GP14	rs415430	rs169201^†^	4.44	8.87E−06	0.982
	17q21.32	*FAM215B*	GP14	rs415430	rs199439^*^	4.49	7.17E−06	0.974
	17q21.31	*KANSL1*	GP14	rs169201	rs17692129^*^	4.48	7.59E−06	0.731
Brain_Cerebellum	17q21.31	*ARL17A*	GP14	rs415430	rs199443^*^	4.66	3.09E−06	0.977
	17q21.32	*FAM215B*	GP14	rs415430	rs169201^†^	4.78	1.74E−06	0.981
	17q21.31	*LRRC37A2*	GP14	rs415430	rs169201^†^	4.49	7.14E−06	0.982
Breast_Mammary_Tissue	17q21.1	*ORMDL3^b^*	GP7	rs4795400	rs25645^*^	5.12	3.02E−07	0.028
	22q13.1^a^	*MIEF1*	GP23	rs909674	rs738288^*^	7.50	6.37E−14	0.501
Adipose_Visceral_Omentum	22q13.1^a^	*MGAT3^b^*	GP23	rs909674	rs5750830^*^	7.96	1.74E−15	0.729
	22q13.1^a^	*RPL3*	GP23	rs1005522	rs139393^*^	5.52	3.43E−08	0.098
Brain_Hypothalamus	21q22.2	*PSMG1*	GP19	rs7282582	rs2297256^*^	5.02	5.12E−07	0.799
Brain_Substantia_nigra	6p21.33^a^	*VARS*	GP19	rs2523591	rs2523500^*^	4.86	1.17E−06	0.097

Detailed naming and compositional information of GPs was given in previous reports and in **Supplementary Table 1**.

GP, IgG N-glycan peak; GP1-24, Zagreb code for the names of GPs in the qualification and quantification of enzymatically released IgG N-glycans by ultra-performance liquid chromatography (UPLC); Lead QTL, SNP with the strongest association in the locus in GWAS analysis; Best eQTL, SNP with the strongest association in the locus in TWAS analysis; COLOC PP.H4, the posterior probability of hypothesis 4 in COLOC approach.

a Regions have been implicated in previous IgG N-glycosylation GWAS.

b Genes have been implicated in previous IgG N-glycosylation GWAS.

^*^The tissue-specific SNP.

^†^The coassociated SNPs.

^§^The tissue-sharing SNPs.

According to the three immune tissues selected by the complementary strategy, 22 statistically significant TWAS hits were obtained with the threshold of Bonferroni correction, nine of which were also identified in the 55 TWAS hits based on the primary strategy. The LDSC-SEG strategy obtained more information from candidate genes in the more functionally relevant tissues and covered the majority result from the three immune tissue strategy (OR is not estimable, *p* < 1.0E−05, Fisher’s exact test). Furthermore, in terms of hiring the most mechanistically related tissue reference panels in TWAS analysis that could reduce the reference bias (21), we therefore chose the results from a primary strategy based on tissue enrichment as the final TWAS result for further analyses. Of the 55 candidate genes identified by TWAS, 12 genes in eight regions (*B4GALT1*: 9p21.1; *COG7*: 16p12.2; *FUT8*: 14q23.3; *GPANK1*: 6p21.33; *GSDMB*: 17q21.1; *HLA-C*: 6p21.33; *HLA-DRA*: 6p21.32; *KDELR2*: 7p22.1; *MGAT3*: 22q13.1; *ORMDL3*: 17q21.1; *RPL3*: 22q13.1; and *TAB1*: 22q13.1) are in known IgG N-glycosylation GWAS susceptibility loci (9–11). Forty-three genes at 10 novel regions and six known regions were for the first time associated with IgG N-glycosylation (**Table 1**).

**Figure 4:**
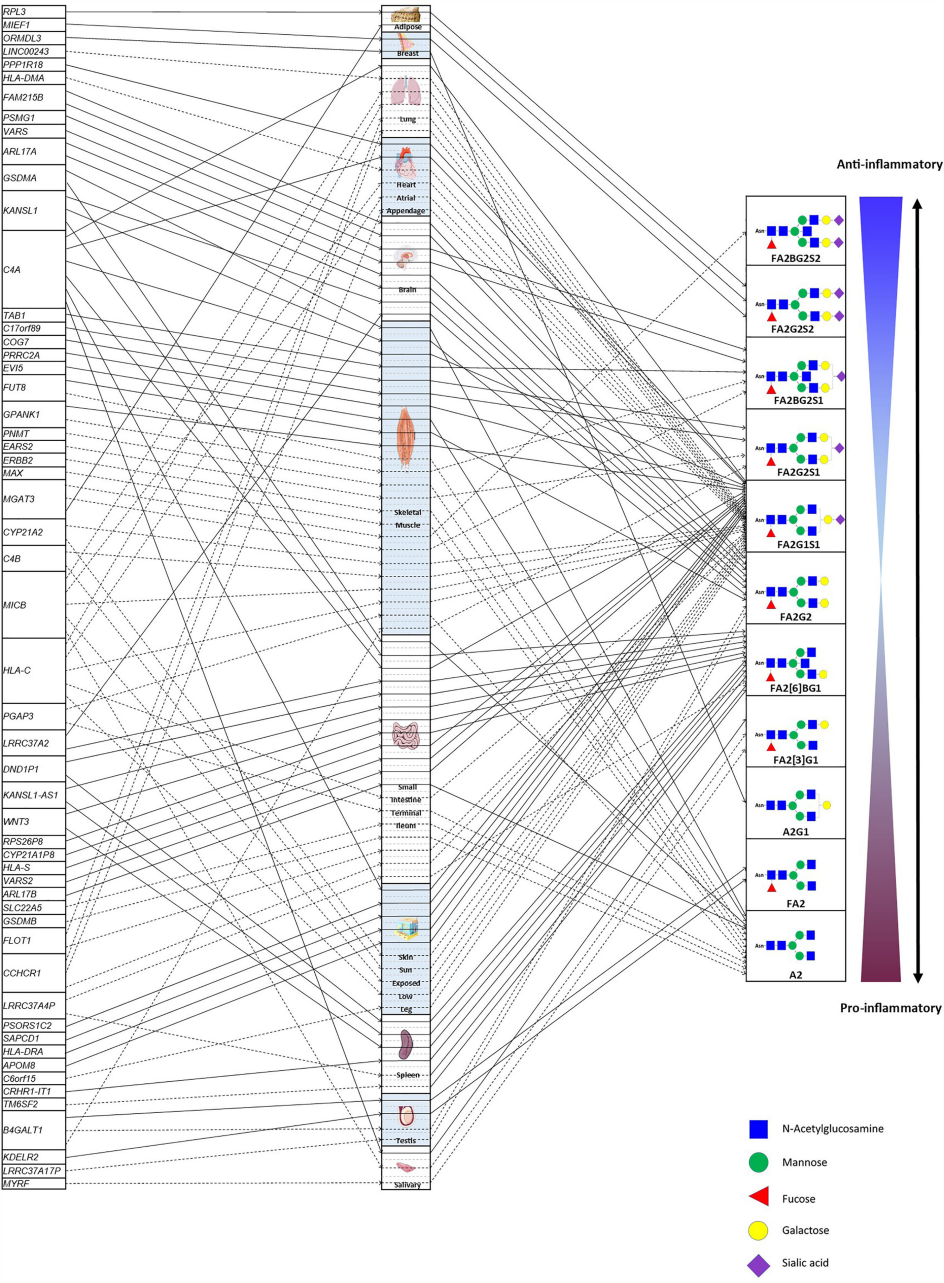
The atlas of TWAS statistically significant genes regulating IgG N-glycosylation within specific tissues. The left column lists the candidate genes of IgG N-glycosylation. The middle column comprises the tissues enriched to the candidate genes of IgG N-glycosylation. The various types of brain tissue from the GETx consortium (v7) were allocated to the “brain” tissue group to reduce FDR. The right column indicates the IgG N-glycosylation traits associated with candidate genes within relevant tissues. F, core (if the first letter) or antennary fucose; A2, biantennary; B, bisecting N-acetylglucosamine; Gx, galactose; Sx, sialic acid; x, number of galactoses or sialic acids in a glycan structure. Detailed naming and compositional information of GPs was given in previous reports (9, 20) and is listed in **Supplementary Table 1**. The solid lines with arrows show the positive effects of the expression of candidate genes on the specific IgG N-glycosylation trait, whereas the dotted lines with arrows show negative effects.

Based on the results from 27 IgG N-GP-enriched tissue types and 17 corresponding GPs, TWAS eventually identified 14 tissues significantly specific to 11 IgG N-GPs, and meanwhile the expressions of 55 genes were linked to their corresponding IgG N-GPs (**Figure 4**). Checking for the IgG N-glycan traits based on the chemical and structural properties of glycans (detailed chemical and structural information of GPs was given in previous reports (9, 20) and is presented in **Supplementary Table 2**), all 14 types of specific tissue and 45 genes contributed to galactosylation, including eight tissues and 31 genes contributing to monogalactosylation, and 8 tissues and 17 genes contributing to digalactosylation; 13 of 14 tissues and 39 genes were identified as involved in fucosylation; 9 tissues and 30 genes affected sialylation, including 7 tissues and 28 genes to monosialylation, and 3 tissues and 3 genes to disialylation. Only 5 tissues and 14 genes were significantly associated with bisecting GlcNAc (**Table 1** and **Supplementary Table 2**).

From the confirmed 90 sets of “GP-SNP-gene expression” associations, three scenarios of genetic variants affecting IgG N-glycosylation traits were observed: (1) A single SNP is within or associated with a single gene and a single GP within one corresponding tissue, showing the tissue-specific effect of the genetic variant for a certain GP. In this scenario, 40 associations were observed (**Table 1** SNPs with asterisks). (2) A single SNP is within or associated with a single gene or multiple genes and simultaneously associated with a single or multiple GPs within multiple corresponding tissues. This suggests pleiotropic effects of the genetic variants for different GPs or genes among multiple tissues (accounting for 44 observed associations, **Table 1** SNPs with daggers). (3) A single SNP is associated or located in a single gene but associated with multiple GPs within only one tissue, indicating that the genetic variant is coassociated with these two GPs while they were highly correlated with each other *via* coassociation with the same gene (accounting for six observed associations, **Table 1** SNPs with section symbols). For example, rs761830 is correlated with A2 glycan (GP2) (*Z* = −11.70, *p* = 1.34E−31) and FA2G2 glycan (GP14) (*Z* = 4.52, *p* = 6.22E−06), linked with *FUT8* gene in skeletal muscle in two reverse directions. In such a case, the corresponding SNP is likely to regulate its target genes in two opposite directions to influence these two GPs (**Table 1**).

### IgG N-Glycosylation TWAS Hits Are Driven by Genetic Regulation on Gene Expression

As multiple TWAS hits overlapped with the significant results of previous IgG N-glycosylation GWAS, we conducted joint and conditional analyses to address how much GWAS signal remains after the association of the functional annotation is removed. The postprocess module in FUSION was performed to report the statistics for the jointly significant genes.

**Figure 5:**
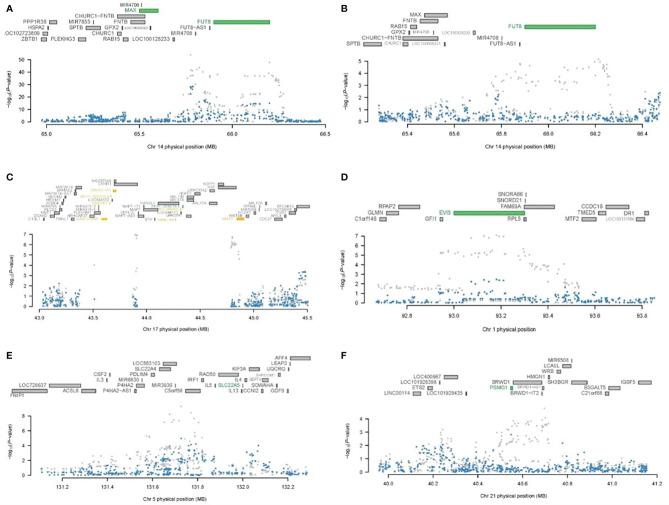
Regional association of TWAS hits. **(A)** Chromosome 14 regional association plot for GP3 in skeletal muscle. **(B)** Chromosome 14 regional association plot for GP14 skeletal muscle. **(C)** Chromosome 17 regional association plot for GP10 in the small intestine terminal ileum. **(D)** Chromosome 1 regional association plot for GP3 in skeletal muscle. **(E)** Chromosome 5 regional association plot for GP3 in the small intestine terminal ileum. **(F)** Chromosome 21 regional association plot for GP19 in brain hypothalamus. The top panel in each plot highlights all genes in this 1-Mb window. The marginally significant genes identified by TWAS are colored in orange, and the jointly significant genes are highlighted in green. The bottom panel shows a Manhattan plot of the GWAS data before (grey) and after (blue) conditioning on the predicted expression of the green genes.

Our results demonstrated that all known IgG N-glycosylation GWAS susceptibility loci could be explained entirely or mostly by the expression of corresponding genes identified in TWAS, supporting the concept that these TWAS hits were mainly driven by the genetic regulation of gene expression in these loci (**Supplementary Table 4** and **Supplementary Figure 2**). For instance, association analysis conditioning on the expression of *FUT8*, which depended on the associations between expression SNPs (eSNPs) and A2 glycan (GP2) in skeletal muscle, showed expression-driven signals in a previously implicated GWAS locus and explained 59.1% of the variance (rs761830 lead SNP_GWAS_ *p* = 1.32E−54, conditional lead SNP_GWAS_ *p* = 2.49E−23) (**Figure 5A**). By the associations of eSNPs with FA2G2 glycan (GP14) in skeletal muscle, the expression level of *FUT8* explained 94.7% of the variances at this locus (rs761830 lead SNP_GWAS_ *p* = 6.83E−06, conditional lead SNP_GWAS_ *p* = 3.00E−01) was observed (**Figure 5B**). The joint and conditional analyses for *WNT3* completely explained the variance of the locus by 100% on chromosome 17 through the associations of eSNPs with FA2[6]BG1 glycan (GP10) in the small intestine terminal ileum (rs199438 lead SNP_GWAS_ *p* = 1.3E−07, conditional lead SNP_GWAS_ *p* = 1) (**Figure 5C**).

Similarly, we performed joint and conditional analyses on the remaining 43 novel IgG N-glycosylation TWAS hits with the expression of corresponding genes. The result showed that the majority of these TWAS hits were also mostly driven by the regulatory effect of genetic variants on the expression levels of targeted genes (**Supplementary Figure 3** and **Supplementary Table 5**). For example, association analysis conditioning on the expression of *EVI5* which depended on the associations between eQTLs and A2 glycan (GP2) in the skeletal muscle panel, demonstrated that expression-driven signals in this novel IgG N-glycosylation locus explained 49.9% of the variance (rs169201 lead SNP_GWAS_ *p* = 5.17E−10, conditional lead SNP_GWAS_ *p* = 1.09E10-05) (**Figure 5D**). Association analysis conditioning on the expression of *SLC22A5* which depended on the associations between eQTLs and A2 glycan (GP2) in the small intestine terminal ileum panel, showed expression-driven signals in this novel locus that explained 55.7% of the variance (rs738288 lead SNP_GWAS_ *p* = 2.16E−22, conditional lead SNP_GWAS_ *p* = 9.19–11) (**Figure 5E**). For a novel locus of IgG N-glycosylation identified by TWAS in 21q22.2, conditioning on PSMG1 explained 95.1% of the variance (rs8065126 lead SNP_GWAS_ *p* = 1.63E−05, conditional lead SNP_GWAS_ *p* = 3.42E−01) (**Figure 5F**).

### Colocalization of TWAS Signals Provides Evidence of Causality

To strengthen the detection of candidate genes as potential causal genes, the colocalization analyses were conducted between total 90 TWAS hits and all corresponding GTEx v7 eQTLs (15), using “*coloc*” package (37).

The colocalization results for this current study, the posterior probability for the fifth hypothesis (colocalized function/GWAS related), are given in the last column of **Table 1**. Using 0.750 as the cutoff value here, 38 out of a total 90 TWAS hits obtained significant possibilities for the colocalization shared between GWAS association and eQTL association (**Table 1**). It is particularly important for the TWAS hits in the extended patterns of LD regions, such as HLA region on chromosome 6. Only 14 out of 40 TWAS hit in HLA region had the significant possibilities for colocalization, hinting that the extended patterns of LD regions are still challenging for TWAS approaches. Half of TWAS hits in nonextended patterns of LD regions were declared as shared causal variants with supportive posterior possibilities.

### Functional Annotation

We used *HaploReg* v4.1 to perform the functional analysis of a total of 18 lead eQTLs showing the best colocalized associations with IGPs in both TWAS and GWAS results. Among them, rs2297256 was in the 3′-UTR, while rs2516412, rs2534680, rs3101018, rs761830, and rs1005522 were located in the upstream transcript regions, and the remaining 12 lay in the intronic regions (**Supplementary Table 6**).

According to the data from the Encyclopedia of ENA Elements (ENCODE) project (43), 16 out of the total 18 lead eQTLs (except rs169201 and rs199520) were identified in strong promoter or/and enhancer activity regions; 15 of the total 18 (except rs1144709, rs169201, and rs199520) in DNAse hypersensitivity site regions; rs2516421, rs2534680, rs3101018, and rs17198191 in transcription factor-binding regions; 14 of the total 18 (except rs3130484, rs761830, rs9303281, and rs17689471) in the regulatory motifs (**Supplementary Table 6**).

### Gene Set Enrichment and PPI Network Analysis

To investigate how many biological pathways may be potentially relevant with the genes identified by TWAS, enrichment analyses for GO, KEGG pathways, and Reactome were performed using the STRING database (see URLs in the *Data Availability Statement*). A total of 55 candidate genes identified by TWAS were significantly enriched in 42 gene sets focusing on three functional processes: glycosylation (10 gene sets), immune response (23 gene sets), and protein translation (9 gene sets) (**Table 2**).

**Table 2:** Significant pathways of TWAS genes identified through gene network analysis.

GO	Category	Description	*p-*value	Hits
hsa_M00075	KEGG pathway	N-Glycan biosynthesis, complex type	4.59E−06	FUT8|B4GALT1|MGAT3
R-HSA-975576	Reactome gene sets	N-Glycan antennae elongation in the medial/trans-Golgi	2.10E−05	FUT8|B4GALT1|MGAT3
R-HSA-948021	Reactome gene sets	Transport to the Golgi and subsequent modification	4.00E−05	FUT8|B4GALT1|MGAT3|KDELR2|COG7
hsa00510	KEGG pathway	N-Glycan biosynthesis	1.44E−04	FUT8|B4GALT1|MGAT3
R-HSA-446203	Reactome gene sets	Asparagine N-linked glycosylation	4.07E−04	FUT8|B4GALT1|MGAT3|KDELR2|COG7
GO:0006487	GO biological processes	Protein N-linked glycosylation	5.69E−04	FUT8|B4GALT1|MGAT3
GO:0043413	GO biological processes	Macromolecule glycosylation	2.08E−03	FUT8|B4GALT1|MGAT3|COG7
GO:0006486	GO biological processes	Protein glycosylation	2.08E−03	FUT8|B4GALT1|MGAT3|COG7
GO:0070085	GO biological processes	Glycosylation	2.41E−03	FUT8|B4GALT1|MGAT3|COG7
GO:0009101	GO biological processes	Glycoprotein biosynthetic process	5.97E−03	FUT8|B4GALT1|MGAT3|COG7
hsa05150	KEGG pathway	*Staphylococcus aureus* infection	5.51E−06	C4A|C4B|HLA-DMA|HLA-DRA
hsa05330	KEGG pathway	Allograft rejection	6.69E−05	HLA-C|HLA-DMA|HLA-DRA
hsa05332	KEGG pathway	Graft-versus-host disease	8.42E−05	HLA-C|HLA-DMA|HLA-DRA
hsa04940	KEGG pathway	Type I diabetes mellitus	9.72E−05	HLA-C|HLA-DMA|HLA-DRA
hsa05322	KEGG pathway	Systemic lupus erythematosus	1.66E−04	C4A|C4B|HLA-DMA|HLA-DRA
hsa05320	KEGG pathway	Autoimmune thyroid disease	1.82E−04	HLA-C|HLA-DMA|HLA-DRA
hsa05416	KEGG pathway	Viral myocarditis	2.50E−04	HLA-C|HLA-DMA|HLA-DRA
hsa05140	KEGG pathway	Leishmaniasis	4.69E−04	HLA-DMA|HLA-DRA|TAB1
hsa04612	KEGG pathway	Antigen processing and presentation	5.48E−04	HLA-C|HLA-DMA|HLA-DRA
hsa05168	KEGG pathway	Herpes simplex infection	5.83E−04	HLA-C|HLA-DMA|HLA-DRA|TAB1
hsa05145	KEGG pathway	Toxoplasmosis	1.66E−03	HLA-DMA|HLA-DRA|TAB1
hsa05166	KEGG pathway	HTLV-I infection	1.94E−03	HLA-C|HLA-DMA|HLA-DRA|WNT3
GO:0002250	GO biological processes	Adaptive immune response	2.68E−03	C4A|C4B|HLA-C|HLA-DMA|HLA-DRA|MICB
hsa04514	KEGG pathway	Cell adhesion molecules (CAMs)	3.31E−03	HLA-C|HLA-DMA|HLA-DRA
GO:0002253	GO biological processes	Activation of immune response	3.75E−03	C4A|C4B|HLA-DRA|MICB|FLOT1|TAB1
hsa04145	KEGG pathway	Phagosome	4.00E−03	HLA-C|HLA-DMA|HLA-DRA
GO:0045807	GO biological processes	Positive regulation of endocytosis	4.00E−03	C4A|C4B|FLOT1
GO:0002478	GO biological processes	Antigen processing and presentation of exogenous peptide antigen	5.71E−03	HLA-C|HLA-DMA|HLA-DRA
GO:0002449	GO biological processes	Lymphocyte-mediated immunity	6.08E−03	C4A|C4B|HLA-C|MICB
GO:0019884	GO biological processes	Antigen processing and presentation of exogenous antigen	6.36E−03	HLA-C|HLA-DMA|HLA-DRA
GO:0002460	GO biological processes	Adaptive immune response based on somatic recombination of immune receptors built from immunoglobulin	6.71E−03	C4A|C4B|HLA-C|MICB
GO:0048002	GO biological processes	Antigen processing and presentation of peptide antigen	7.16E−03	HLA-C|HLA-DMA|HLA-DRA
hsa05169	KEGG pathway	Epstein-Barr virus infection	8.35E−03	HLA-C|HLA-DRA|TAB1
R-HSA-379724	Reactome gene sets	tRNA aminoacylation	9.05E−05	VARS|VARS2|EARS2
GO:0006418	GO biological processes	tRNA aminoacylation for protein translation	1.11E−04	VARS|VARS2|EARS2
GO:0043039	GO biological processes	tRNA aminoacylation	1.35E−04	VARS|VARS2|EARS2
GO:0043038	GO biological processes	amino acid activation	1.44E−04	VARS|VARS2|EARS2
hsa00970	KEGG pathway	Aminoacyl-tRNA biosynthesis	3.48E−04	VARS|VARS2|EARS2
R-HSA-72766	Reactome gene sets	Translation	3.07E−03	RPL3|VARS|VARS2|EARS2
GO:0006399	GO biological processes	tRNA metabolic process	7.47E−03	VARS|VARS2|EARS2
GO:0055088	GO biological processes	Lipid homeostasis	3.79E−03	TM6SF2|APOM|ORMDL3
GO:0006612	GO biological processes	Protein targeting to membrane	8.46E−03	ERBB2|RPL3|MIEF1

The ten significantly enriched glycosylation-relevant gene sets were mainly involved in the pathways of N-glycan biosynthesis, e.g., N-glycan biosynthesis, complex type (Mann-Whitney *U* test, *p* = 4.59E−06), transport to the Golgi and subsequent modification (*p* = 4.00E−05), N-glycan antennae elongation in the medial/trans-Golgi (*p* = 2.10E−05), and asparagine N-linked glycosylation (*p* = 4.07E−04). Twenty-one out of the 24 immune response-related gene sets were mainly enriched with infections, such as *Staphylococcus aureus* infection (*p* = 1.40E−05), allograft rejection (*p* = 5.60E−04), graft-versus-host disease (*p* = 5.60E−04), and several specific immunological diseases, e.g., systemic lupus erythematosus (SLE) (*p* = 1.66E−04), autoimmune thyroid disease (AHD) (*p* = 1.82E−04), viral myocarditis (*p* = 2.50E−04), and leishmaniasis (*p* = 5.50E−04). At last, nine gene sets were related to protein translation, e.g., tRNA aminoacylation (*p* = 9.05E−05), amino acid activation (*p* = 1.44E−04), and tRNA metabolic process (*p* = 7.47E−03) (**Table 2**).

GO enrichment on TWAS hits strengthened several pathways which are biologically relevant to IgG N-glycosylation. The known glycosyltransferase genes (8) in our TWAS hits, including *FUT8*, *B4GALT1*, *MGAT3*, *KDELR2*, and *COG7* were enriched in the processes of asparagine N-linked glycan biosynthesis, transport to the Golgi and subsequent modification, and N-glycan antennae elongation in the medial/trans-Golgi glycosylation. These processes have been implicated in the physioregulation of IgG N-glycosylation (44, 45). By KEGG pathway analysis, six genes, i.e., *C4A*, *C4B*, *FCGR2A*, *HLA-DMA*, *HLA-DRA*, and *HLA-DRB1*, were enriched as the top 2 significant results in the pathways of *Staphylococcus aureus* infection (KEGG term hsa05322, FDR = 1.42E−05) and systemic lupus erythematosus (KEGG term hsa05150, FDR = 2.00E−04). Three of these genes (*HLA-DMA*, *HLA-DRA*, and *HLA-DRB1*) were enriched in the pathways of several autoimmune and inflammatory diseases, i.e., autoimmune thyroid disease, IBD, RA, and asthma (KEGG terms in **Table 2**), demonstrating their core functions in the pathways.

Through these core genes, these pathways are highly associated with cytokine-cytokine receptor interaction (hsa04060), antigen processing and presentation (hsa04612), T-cell receptor signaling pathway (hsa04660), B-cell receptor signaling pathway (hsa04662), and leukocyte trans-endothelial migration (hsa04670). These pathways play crucial roles in the appropriate functioning of all immunoglobulins, especially for the most abundant type in plasma, i.e., IgG. From Reactome (see URLs in the *Data Availability Statement*) enrichment, three gene sets are enriched into three N-glycosylation pathways, i.e., asparagine N-linked glycosylation (HSA-446203), transport to the Golgi and subsequent modification (HSA-948021), and N-glycan antennae elongation in the medial/trans-Golgi (HSA-975576).

To validate whether these TWAS-identified candidate genes associated with IgG N-glycosylation were inclined to be coexpressed within corresponding tissues, we measured the protein-protein interaction (PPI) network for the connectivity of the genes by GeneNetwork v2.0 (see URLs in the *Data Availability Statement*). The genes were clustered based on their coexpression of public RNA-seq data (*n* = 31,499). Most genes identified by TWAS within each specific tissue reference panel demonstrated coexpression (**Supplementary Figure S4**). For example, skeletal muscle contributed the most numerous significant TWAS hits across all tissues by 24 candidate genes, while 20 genes were clustered into one network with *ERBB2*, *PGAP3*, and *PNMT* demonstrating strong coexpression (**Supplementary Figure 4**). In small intestine terminal ileum, all 18 genes identified by TWAS show coexpression, in which *PGAP3*, *GSDMB*, *HLA-S*, *CCHCR1*, *VARS2*, and *KANSL1* are intensively coexpressed (**Supplementary Figure 4**). Therefore, the coexpressed gene sets within corresponding tissues could support the result of IgG N-glycosylation TWAS analyses based on tissue enrichment by LDSC-SEG.

### Phenome-Wide Association Study and Genetic Correlation Analysis

To identify other phenotypes which are likely to be associated or comorbid with IgG N-glycosylation, we conducted a pheWAS for each IgG N-glycosylation eQTL in the GWAS database based on a European population (41). Since all eQTLs are associated with IgG N-glycosylation, we chose to exclude and remove the duplicated phenotypes related to the same eQTL from the result list, in order to emphasize the remaining top 5 phenotypes ranked by *p* for each eQTL. In total, nearly 100 phenotypes were identified as significantly associated with these eQTLs, including anthropometric health measurements (weight, height, blood pressure, and blood cell count), immune and metabolic diseases (SLE, inflammatory bowel disease (IBD), rheumatoid arthritis (RA), primary sclerosing cholangitis (PSC), and type 2 diabetes (T2D)), and neurological and psychiatric disorders (Parkinson’s disease (PD), schizophrenia, and bipolar disorder) (**Supplementary Table 7**).

**Figure 6:**
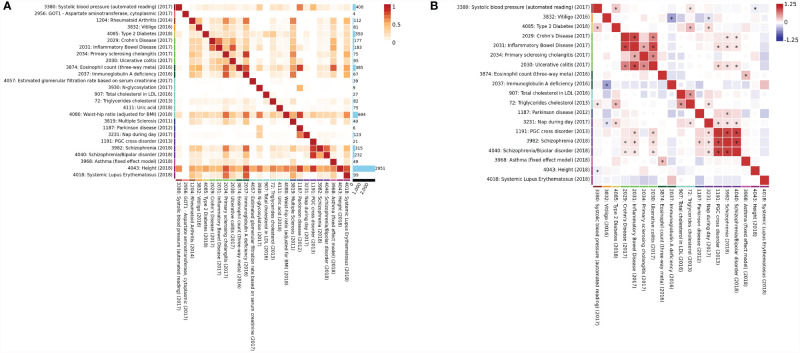
Multimarker analysis and genetic correlation. **(A)** Multi-marker Analysis of GenoMic Annotation (MAGMA) genes overlap. Each cell represents the proportion of overlapped significant genes (*p*-value <2.5E−06) between the two GWAS on the number of significant genes in both GWAS on the rows and columns and divided by the number of significant genes in each GWAS. Rectangles next to the trait labels are colored based on the domain of the trait. **(B)** Genetic correlation. An asterisk in the box indicates the correlation passes the Bonferroni significance threshold (*p* < 0.05). Rectangles next to the trait labels are colored based on the domain of the trait. Phenotypes are clustered into 24 domains and derived from public genome-wide association study summary statistics.

To reconfirm the pheWAS results, we investigated the genetic correlations between these phenotypes using the most recent GWAS data from the UK Biobanks by Multi-marker Analysis of GenoMic Annotation (MAGMA), and SNP heritability, and genetic correlation with LDSC (41). Most of these disease-related phenotypes were implicated in previous GWAS studies (46–51). By analyzing the genes assigned to each significant SNP within a 1-kb window from both sides with default parameters (SNP-wise mean model) (52) and the gene set defined by MSigDB v.6.1 (53), the MAGMA results showed strong correlations between height, waist-hip ratio (WHR), systolic blood pressure (SBP), PD, and IBD with IgG N-glycosylation (*p* < 2.5E−06) (**Figure 6A**). Through the calculation of the SNP heritability and pairwise genetic correlations by LDSC (22), genetic correlations were calculated between the GWAS of disease-related phenotypes. The results showed strong positive correlations between IgG N-glycosylation, SBP, triglyceride cholesterol (TC), T2D, IBD, PSC, Crohn’s disease (CD), and ulcerative colitis (UC), consistent with the above pheWAS results (**Figure 6B**).

## Discussion

In this study, we conducted a systematic transcriptome-wide association study, combining the analysis of LDSC-SEG and TWAS to gain insight into the tissue specificity and tissue-dependent genetic effect of IgG N-glycosylation, based on summary statistics of the most recent IgG N-glycosylation GWAS on 8,090 individuals of European ancestry. In so doing, we addressed fundamental questions regarding the heritable tissue enrichment of IgG N-glycosylation and constructed an atlas of tissue-dependent associations between genes and IgG N-glycan traits.

By the enrichment of 17 IgG N-GPs in 27 biologically relevant tissues in our study, we have obtained strong genetic evidence that most MALTs, e.g., skin, lung, breast mammary tissue, small intestine, esophagus muscularis, testis, and uterus, are enriched to specific IgG N-glycan traits (**Figure 2**). It has been established that the IgG secreting cells (i.e., plasma cells) mature from B lymphocytes and are translocated from primary lymphoid tissue (bone marrow) to secondary lymphoid tissue which consists of the lymph nodes, spleen and MALT for secreting IgG and initiating adaptive immune responses (14). However, previous studies measured the N-glycosylation of IgG in humans mainly isolated from plasma, obtaining the average profile of whole IgG N-glycome, and therefore a lack of information about the functionally relevant tissues. A wet experiment on laboratory animals provided evidence to support the assumption that IgG against commensal gut bacteria can be synthesized and deposited locally within MALTs in organ-cultured pig small intestinal mucosal explants (54).

Using IgG as a model glycoprotein, our study demonstrates the evidence of genetically regulated gene expression for the tissue selectivity of protein N-glycosylation in eukaryotes. Eukaryotic N-glycosylation in the ER and Golgi apparatus is highly complicated, due to a variety of exoglycosidase and glycosyltransferase reactions. The tissue-selective manifestation of protein N-glycosylation has been observed in recent studies, e.g., between paired tumorigenic and adjacent nontumorigenic colon tissues in humans (55), and between sites within the same proteins from liver and brain tissues in the mouse (56). As a simple glycoprotein, IgG usually contains only one N-glycosylation site in the constant heavy chain region and the N-glycan moieties of IgG have no more than two antennae. But, in fact, hundreds of forms of glycans have been observed at this single N-glycosylation site. Due to the limitation of sampling from diverse human health tissues for N-glycosylation profiling, there is still a lack of evidence from the N-glycome level for the tissue specificity of IgG N-glycosylation. The results of tissue enrichment in our study have bridged the gap and exhibited tissue-selective manifestation for IgG N-glycosylation, leading to the next hypothesis that certain N-glycans of IgG may specifically be modified within some tissues and be affected by tissue-specific environments. Consequently, the current study advances the knowledge of tissue specificity on human IgG N-glycosylation and in turn increases the statistical power for the following TWAS analysis of candidate gene prioritization (21).

In this current study, we demonstrated that the TWAS approach is able to discover more IgG N-glycosylation-related genes without any prior information. We identified 12 candidate genes in eight regions are in known susceptibility loci reported by previous IgG N-glycosylation GWAS (9–11). Furthermore, we discovered 43 genes at 10 novel regions and six known regions for the first time to be associated with IgG N-glycosylation. For the known genome loci, TWAS discovered more candidate genes whose expressions in specific tissue are likely related to IgG N-glycosylation by integrating GWAS signals with eQTL knowledge, for example, *C4A*, *C4B*, *CYP21A2*, and *GPANK1* in 6p21.33 for FA2G1S1 glycan (GP16) in skeletal muscle. In addition, TWAS explored candidate genes in novel genomic loci, e.g., *EVI5* in 1p22.1 and *PGAP3*, *ERBB2*, *PNMT*, and *GSDMA* in 17q12 for A2 glycan (GP2) in skeletal muscle. These results provided more genomic context, including candidate genes, regulatory variants, and relevant tissues for future functional studies of IgG N-glycosylation.

A gene expressed specifically in a tissue type or cell type usually reflects the biological processes in which the gene is involved and its biological functions (57). In the current study, we demonstrate that associations along IgG N-GP/SNP/gene expression in a tissue-specific SNP scenario appear to be dependent on using expression data derived from N-GP-enriched tissue. For instance, FA2G1S1 glycan (GP16) is positively associated with the expression of PSORS1C2 in sun-exposed lower leg skin *via* a single eSNP rs1265099 (*Z* = 5.24, *p* = 1.65E−07) but is not associated with any other genes or tissues. *PSORS1C2* encodes a keratinocyte cornification-associated protein, which is specifically expressed in two skin tissue types evaluated by GTEX v7 (**Supplementary Figure 5**). The protein product of this gene plays a primary role in the terminal differentiation of keratinocytes (58). Recent studies have shown that *PSORS1C2* is strongly upregulated in peeling skin disease (59) and is also associated with autoimmune skin diseases including vitiligo (60) and psoriasis (61) by GWAS analyses. Also, aberrant IgG FA2G1S1 glycan (GP16) has been observed to be associated with SLE (62) and colorectal cancer (63). Our finding that this IgG FA2G1S1 glycan (GP16) genetic predisposition (rs1265099) may have tissue-specific effects on the expression of *PSORS1C2* within skin tissue thus supports the hypothesis that skin tissue can contribute to the regulation of IgG FA2G1S1 glycan biosynthesis and/or have the potential to serve as a proxy for several skin-related autoimmune diseases. In total, four genes (*PSORS1C2*, *HLA-DRA*, *APOM*, and *SAPCD1*) were significant in sun-exposed lower leg skin TWAS models. Broadly, half of the significant genes show tissue specificity on IgG N-glycosylation in other TWAS tissue models. These tissue-specific candidate gene sets are a promising source for further investigations into genetic effects underlying the interactions between highly diverse IgG N-GPs and tissue-specific genetic expression within corresponding tissues.

In contrast, genetic variants that affect gene expression levels in multiple tissues are more likely to affect multiple complex traits (64). The pleiotropic gene findings in this study can confirm that IgG N-glycosylation TWAS genes expressed in multiple tissues are more likely to have a wide range of downstream phenotypic consequences (i.e., diverse N-glycosylation modification). As an example in the present study, 19 genes are identified as IgG N-glycosylation candidate genes in the small intestine terminal ileum with 10 pleiotropic and 9 tissue-specific genes. Within the ten pleiotropic genes, *C4A*, *FLOT1*, *HLA-C*, and *WNT3* perform important roles in N-glycosylation-related biological processes, while the remaining nine tissue-specific genes are associated with intestine-related functions, e.g., *FLOT1* is responsible for encoding flotillin 1 and is ubiquitously expressed across all tissue types evaluated by GTEx v7 (**Supplementary Figure 6**). Flotillin 1 localizes to the caveolae and plays a role in several super pathways, e.g., the angiopoietin-like protein 8 regulatory pathway, cytoskeletal signaling, regulation of lipid metabolism and beta-adrenergic signaling. As a tissue-specific gene, GSDMB was reported to be expressed exclusively in the epithelium of the gastrointestinal tract in a highly tissue-specific manner (65). *SLC22A5* was reported as being responsible for carnitine transport across apical membranes of intestinal epithelial cells (66). Pleiotropic genes and eSNPs account for almost half of the other significant TWAS hits. Most of these hits have previously been reported in the three core N-glycosylation-related biological processes, including N-glycosylation, immune response, and protein translation, while tissue-specific genes are mainly annotated as being involved in the physiological activities of corresponding tissues. This result partially explains why plasma IgG N-glycosylation demonstrates a relatively stable holistic pattern on each monosaccharide glycan although based on such complicated branching patterns. The pleiotropic candidate genes maintain the primary patterns of N-glycosylation in most related tissues for a house-keeping N-glycosylated level, whereas the tissue-dependent candidate genes regulate tissue-specific IgG N-glycan patterns to adopt local inflammation, maintaining homeostasis during physiology and pathophysiology processes.

By the evidence of the coassociations among eSNP and IgG N-GPs, our results shed considerable light on the regulatory mechanism of the equilibrium between some pairs of IgG N-glycosylation. For example, in terms of *Z*-score in TWAS results, rs761830 simultaneously correlated with A2 glycan (GP2) (*Z* = −11.70, *p* = 1.34E−31) and FA2G2 glycan (GP14) (*Z* = 4.52, *p* = 6.22E−06) and linked them with *FUT8* gene in skeletal muscle in two reverse directions (**Supplementary Table 2** and **Figure 2**). This linked association provides two layers of meaning. Firstly, in IgG N-glycosylation GWAS statistics, the effect allele (rs761830-A) at the *FUT8* locus is associated with a decreased level of GP2 (rs761830, *p* = 4.08E−38) but an increased level of GP14 (rs761830, *p* = 6.83E−06). The finding indicates that these two GPs share identical heritability, being associated antagonistically in the IgG N-glycome. Conversely, increasing A2 glycan (GP2) and decreasing FA2G2 glycan (GP14) are consistent with the changing of certain IgG N-GPs which have been observed in association studies on chronic diseases (**Supplementary Table 8**), such as dyslipidemia (DL) (67), SLE (62), RA (68), and chronic kidney disease (CKD) (69). Secondly, eQTL statistics of GTEx v7 shows the effect allele rs761830-A is responsible for increased changes in gene expression of *FUT8* in skeletal muscle (rs761830, *p* = 2.60E−06). Hence, the genetic predisposition tagged by SNP rs761830 whose allelic regulation on the expression of *FUT8* gene in skeletal muscle may contribute to the equilibrium of IgG N-GP regulations, such as the above-mentioned two types of N-GPs.

By GO pathway enrichment and pheWAS investigation, our study provides additional evidence for the involvement of IgG N-glycosylation in several IgG N-GP-related diseases. Since the establishment of UPLC-based high-throughput IgG N-glycomic profiling (70), certain specific IgG N-GPs differed significantly from the average patterns and were indicative of clinical conditions, such as the level of monosialylated glycans which was most strongly correlated with MetS-related risk factors, especially with systolic blood pressure (SBP) (71). Further research in other independent cohorts confirmed the existence of a similar relationship between selective IgG N-glycan patterns and signs of autoimmunity, e.g., IBD (49, 72), SLE (62), and RA (73), neurological disorders, e.g., Parkinson’s disease (4) and dementia (74), and vascular diseases, e.g., ischemic stroke (75) and cancers (63), as previously mentioned (**Supplementary Table S7**). The consistency of candidate genes, combined with the coexpression of IgG N-glycosylation-related genes within multiple tissues, GO enrichment gene sets, and the pheWAS investigation on TWAS eSNPs implies that IgG N-glycosylation may be the mediated phenotype sharing genetic correlations with its related diseases. The allelic regulatory genetic variants of IgG N-glycosylation within certain tissues may be involved in the pathophysiological processes of its related diseases *via* regulation of their target genes in tissue-specific modes.

Efforts to generate ever-larger sets of tissue-specific genetic effects data will facilitate data mining opportunities for investigating the regulatory mechanisms underlying trait and gene associations (76). Although the current study has been restricted to the use of eQTL data and GWAS data generated from independent subpopulations of European ancestry with limited sample sizes, future functional genomic endeavors of tissue-specific regulation on IgG N-glycosylation will benefit from a larger replication cohort simultaneously having IgG N-glycosylation features, genotype data, and gene expression profiles from different tissues. A recent study showed that targeted sialic glycan degradation reinforces the anticancer immune response in an animal experiment as evidenced by the sialylation effect on the surface of cancer cells (77). The construction of *in vitro* and *in vivo* models for IgG N-glycosylation are therefore urgently needed for providing more solid experiment-based evidence to confirm the hypothesis generated from the current study. We also note that our study utilized eQTLs as genetic predictors of gene expression. Nevertheless, pleiotropic effects on both gene expression and phenotype could not be ruled out without further analyses. Probabilistic fine-mapping approaches and transcription factor enrichment analyses (78–80) can positively exclude spurious results. Furthermore, a gene may have regulatory characteristics other than *cis*-regulation, e.g., *trans*-regulation, or that does not pass through eQTLs, but still could have downstream effects on expression (81). In theory, beyond the current knowledge of regulatory models, there could be other novel regulatory elements and underlining mechanisms. However, based on allelic *cis*-regulation, we have been able to successfully prioritize a potential causal gene set of 55 genes for IgG N-glycosylation and its related complex traits and diseases.

In summary, we have performed the first comprehensive analysis so far to identify the most relevant tissues for IgG N-glycosylation and to detect a large number of genetic variants which may regulate their target genes and further contribute to IgG N-glycosylation within corresponding tissues. This knowledge provides a starting point for further mechanistic work on IgG N-glycosylation, which would advance our understanding of IgG N-glycosylation biology and guide the design of future functional studies to explore the specific variants and the heritable regulation of IgG N-glycosylation. More importantly, our approach of combining LDSC-SEG and TWAS is widely applicable to complex traits for which GWAS summary statistics are available and helps our understanding of the molecular basis of the trait at multiple omics levels.

## Data Availability Statement

IgG N-glycosylation GWAS summary statistics are available at: https://datashare.is.ed.ac.uk/handle/10283/3238/. Hapmap3 SNPs from 1000 Genomes data are available at: ftp://ftp.1000genomes.ebi.ac.uk/vol1/ftp/release/20130502/. LDSC-SEG software and reference are available at: https://alkesgroup.broadinstitute.org/LDSCORE/. FUSION software and reference LD are available at: http://gusevlab.org/projects/fusion/. For LDSC-SEG, see https://github.com/bulik/ldsc. For FUSION, see http://gusevlab.org/projects/fusion/. For GTEx portal, see http://gtexportal.org/. For coloc, see https://cran.r-project.org/web/packages/coloc/index.html. For HaploReg v4.1, see https://pubs.broadinstitute.org/mammals/haploreg/haploreg.php. For STRING, see http://string-db.org/For GeneNetwork v2.0 see https://genenetwork.nl. For GWASAtlas, see https://atlas.ctglab.nl.

## Author Contributions

WW conceived the design of this study. XL, HW, and YZ performed the statistical and computational analyses. WC, HH, and MS assisted with data management. WW and XL wrote the manuscript. YW, ML, XG, XT, and JH supervised the project and oversaw the manuscript. All authors contributed to the article and approved the submitted version.

## Funding

This work was supported by an Australia-China International Collaborative grant (NHMRC APP1112767-NSFC 81561128020), National Natural Science Foundation of China grant (NFSC 81773527 & 81573215), China Scholarship Council grant (CSC-201708110200), and Bioyong Industry Engagement Scholarship (G1002655) and School of Medical and Health Sciences Seed Grant Scheme 2021 (27223) from Edith Cowan University.

## Conflict of Interest

YZ was employed by the company Beijing Lucidus Bioinformation Technology Co., Ltd.

The remaining authors declare that the research was conducted in the absence of any commercial or financial relationships that could be construed as a potential conflict of interest.

## Publisher’s Note

All claims expressed in this article are solely those of the authors and do not necessarily represent those of their affiliated organizations, or those of the publisher, the editors and the reviewers. Any product that may be evaluated in this article, or claim that may be made by its manufacturer, is not guaranteed or endorsed by the publisher.
