# Supplementary material for: Heritability Enrichment of Immunoglobulin G N-Glycosylation in Specific Tissues
Source: Front Immunol. 2021 Nov 3;12:741705. doi: 10.3389/fimmu.2021.741705 (PMC8595136; doi:10.3389/fimmu.2021.741705)
Supplement: Supplementary file 1 [file DataSheet_1.docx]

**SUPPLEMENTARY FIGURES**

**Supplementary Figure 1 |** Results of tissue enrichment for IgG N-GPs.

**Supplementary Figure 2 |** Regional association of TWAS hits reported by previous GWAS. **Supplementary Figure 3 |** Regional association of novel TWAS hits.

**Supplementary Figure 4 |** The co-expression of IgG N-glycosylation related genes in enriched tissues.

**Supplementary Figure 5 |** *PSORS1C2* gene exp-plot in GTEx v7.

**Supplementary Figure 6 |** *FLOT1* gene exp-plot in GTEx v7.


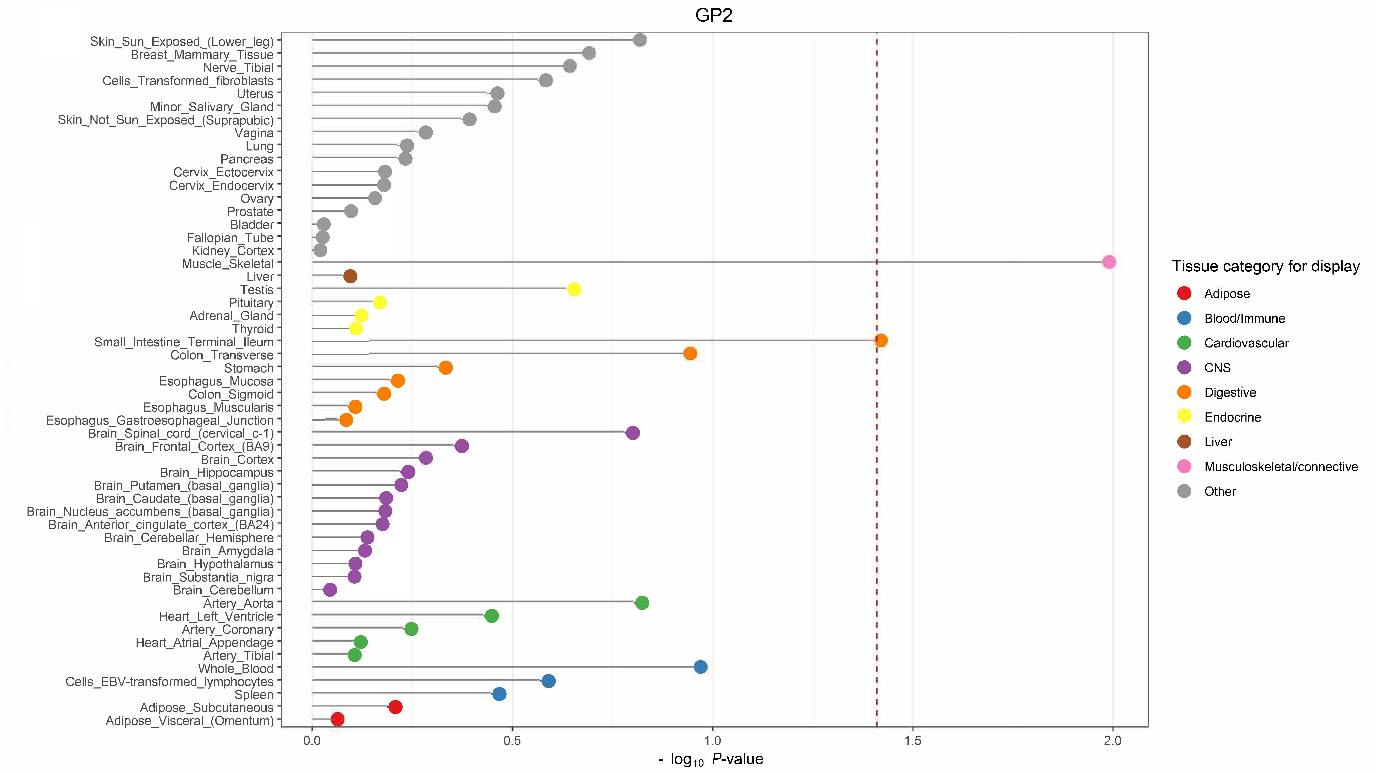


**Supplementary Figure 1.1 |** Results of tissue enrichment for IgG N-GP2. Significantly enriched tissues of IgG N-glycosylation were identified by linkage disequilibrium score regression in a specifically expressed genes (LDSC-SEG) approach. A total of 53 types of tissue obtained from the Genotype-Tissue expression project (GTEx v7) are grouped into nine domains with different colours. Twenty IgG N-glycan peaks (GPs) with significant GWAS results are enriched in all 53 types of tissue. Tissues which are highly enriched for each IgG N-GPs with significant GWAS results across all tissue types in GTEx v7 are shown with a false discovery rate (FDR) significant at 5% (red dotted line).


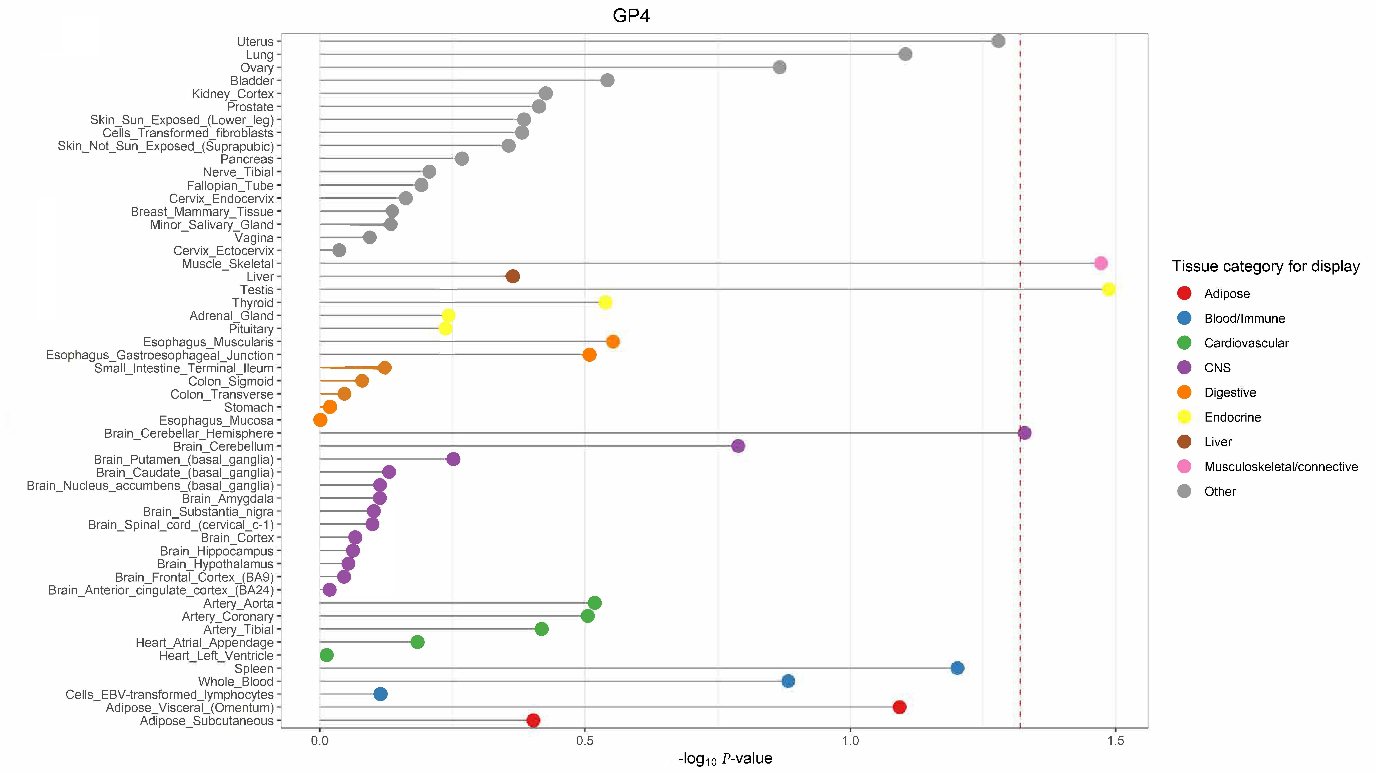


**Supplementary Figure 1.2 |** Results of tissue enrichment for IgG N-GP4. Significantly enriched tissues of IgG N-glycosylation were identified by linkage disequilibrium score regression in a specifically expressed genes (LDSC-SEG) approach. A total of 53 types of tissue obtained from the Genotype-Tissue expression project (GTEx v7) are grouped into nine domains with different colours. Twenty IgG N-glycan peaks (GPs) with significant GWAS results are enriched in all 53 types of tissue. Tissues which are highly enriched for each IgG N-GPs with significant GWAS results across all tissue types in GTEx v7 are shown with a false discovery rate (FDR) significant at 5% (red dotted line).


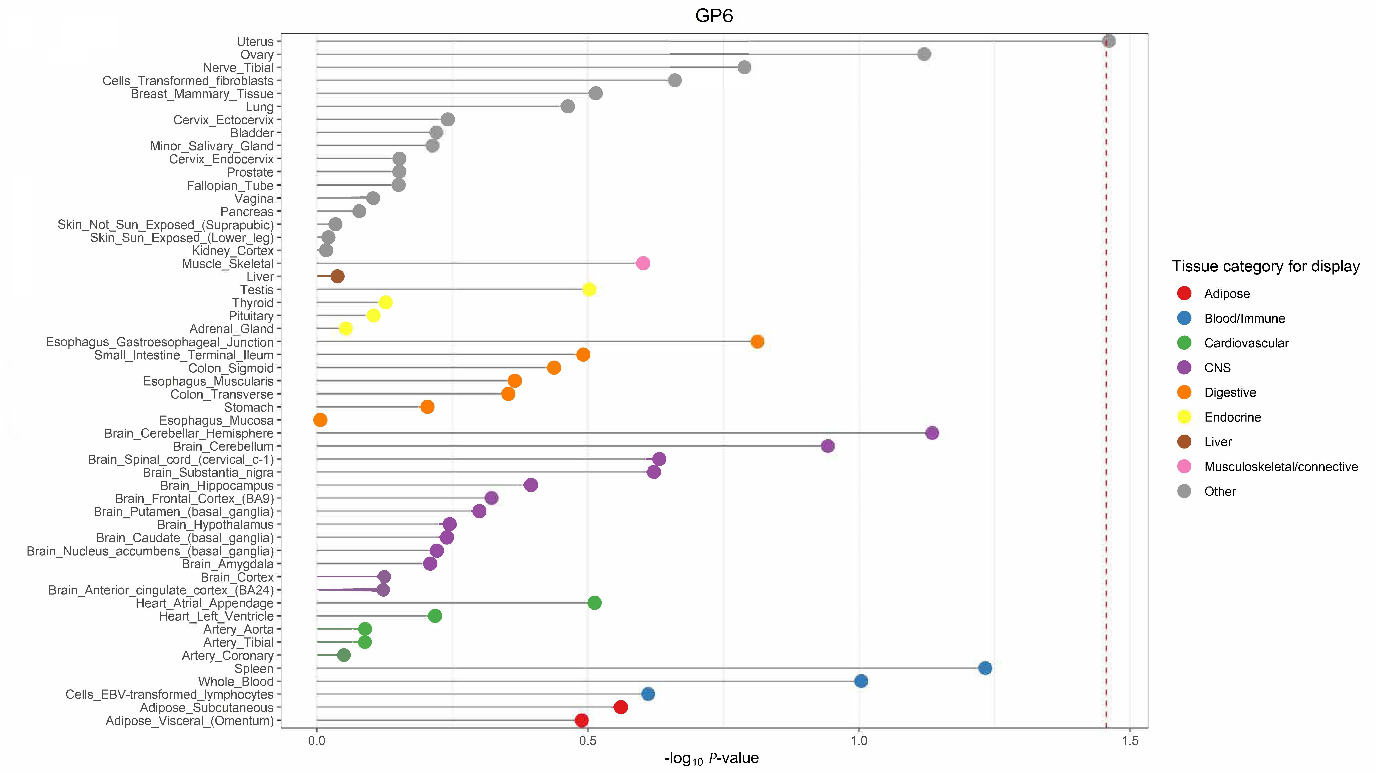


**Supplementary Figure 1.3 |** Results of tissue enrichment for IgG N-GP6. Significantly enriched tissues of IgG N-glycosylation were identified by linkage disequilibrium score regression in a specifically expressed genes (LDSC-SEG) approach. A total of 53 types of tissue obtained from the Genotype-Tissue expression project (GTEx v7) are grouped into nine domains with different colours. Twenty IgG N-glycan peaks (GPs) with significant GWAS results are enriched in all 53 types of tissue. Tissues which are highly enriched for each IgG N-GPs with significant GWAS results across all tissue types in GTEx v7 are shown with a false discovery rate (FDR) significant at 5% (red dotted line).


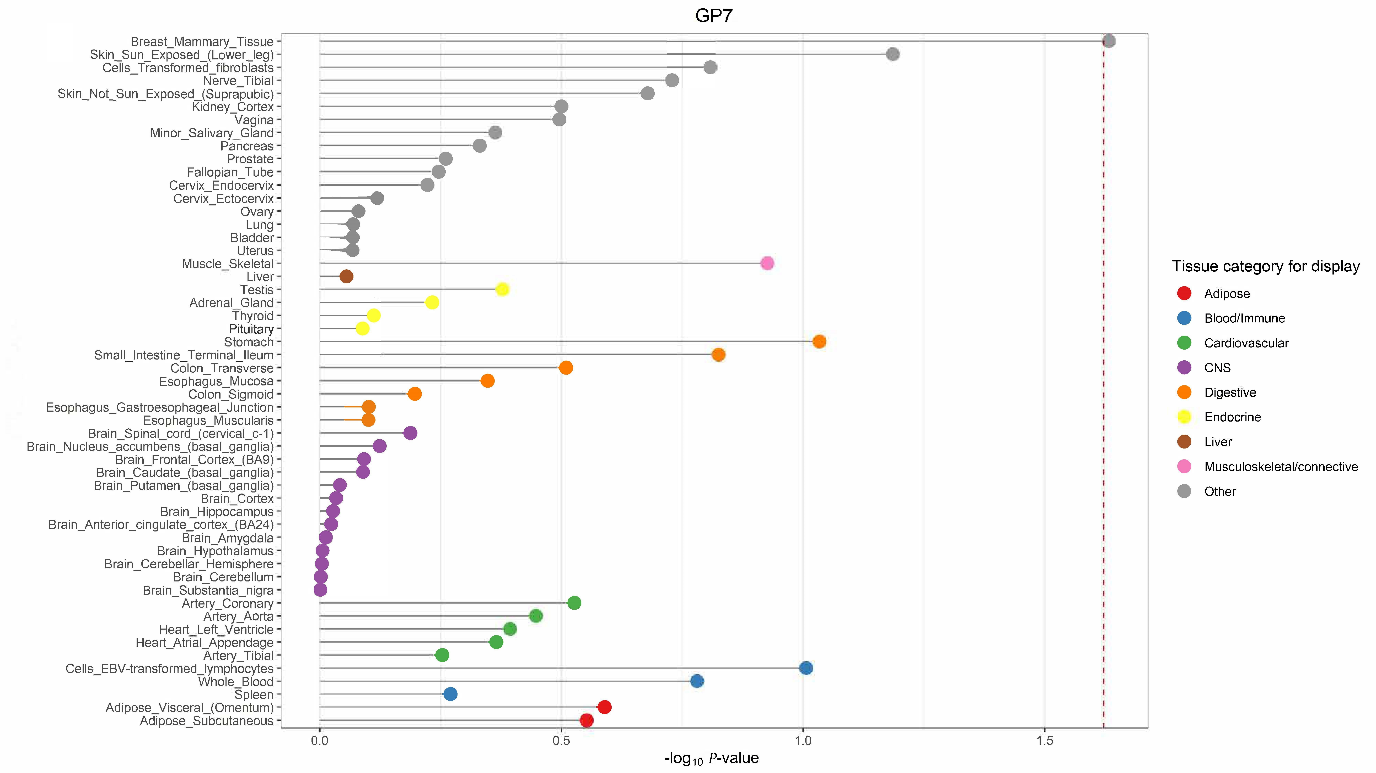


**Supplementary Figure 1.4 |** Results of tissue enrichment for IgG N-GP7. Significantly enriched tissues of IgG N-glycosylation were identified by linkage disequilibrium score regression in a specifically expressed genes (LDSC-SEG) approach. A total of 53 types of tissue obtained from the Genotype-Tissue expression project (GTEx v7) are grouped into nine domains with different colours. Twenty IgG N-glycan peaks (GPs) with significant GWAS results are enriched in all 53 types of tissue. Tissues which are highly enriched for each IgG N-GPs with significant GWAS results across all tissue types in GTEx v7 are shown with a false discovery rate (FDR) significant at 5% (red dotted line).


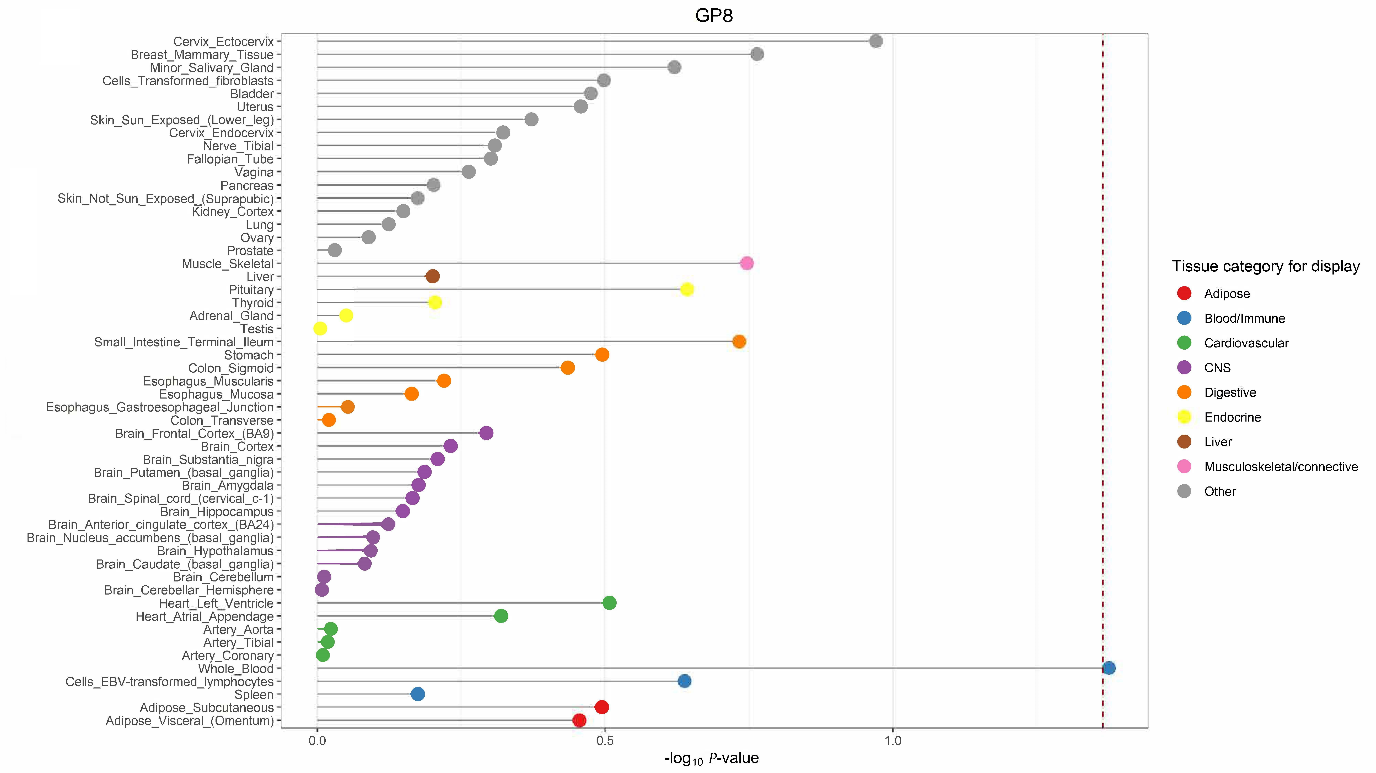


**Supplementary Figure 1.5 |** Results of tissue enrichment for IgG N-GP8. Significantly enriched tissues of IgG N-glycosylation were identified by linkage disequilibrium score regression in a specifically expressed genes (LDSC-SEG) approach. A total of 53 types of tissue obtained from the Genotype-Tissue expression project (GTEx v7) are grouped into nine domains with different colours. Twenty IgG N-glycan peaks (GPs) with significant GWAS results are enriched in all 53 types of tissue. Tissues which are highly enriched for each IgG N-GPs with significant GWAS results across all tissue types in GTEx v7 are shown with a false discovery rate (FDR) significant at 5% (red dotted line).


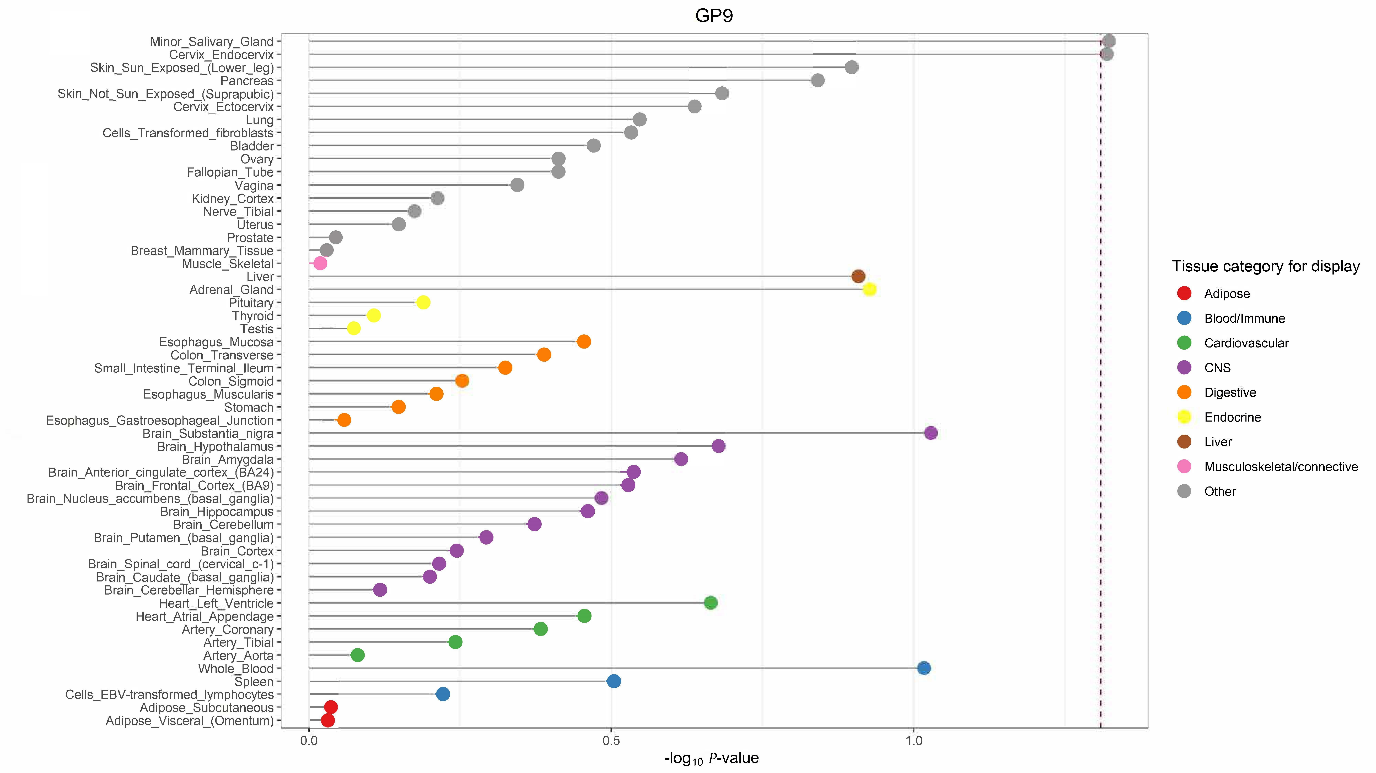


**Supplementary Figure 1.6 |** Results of tissue enrichment for IgG N-GP9. Significantly enriched tissues of IgG N-glycosylation were identified by linkage disequilibrium score regression in a specifically expressed genes (LDSC-SEG) approach. A total of 53 types of tissue obtained from the Genotype-Tissue expression project (GTEx v7) are grouped into nine domains with different colours. Twenty IgG N-glycan peaks (GPs) with significant GWAS results are enriched in all 53 types of tissue. Tissues which are highly enriched for each IgG N-GPs with significant GWAS results across all tissue types in GTEx v7 are shown with a false discovery rate (FDR) significant at 5% (red dotted line).


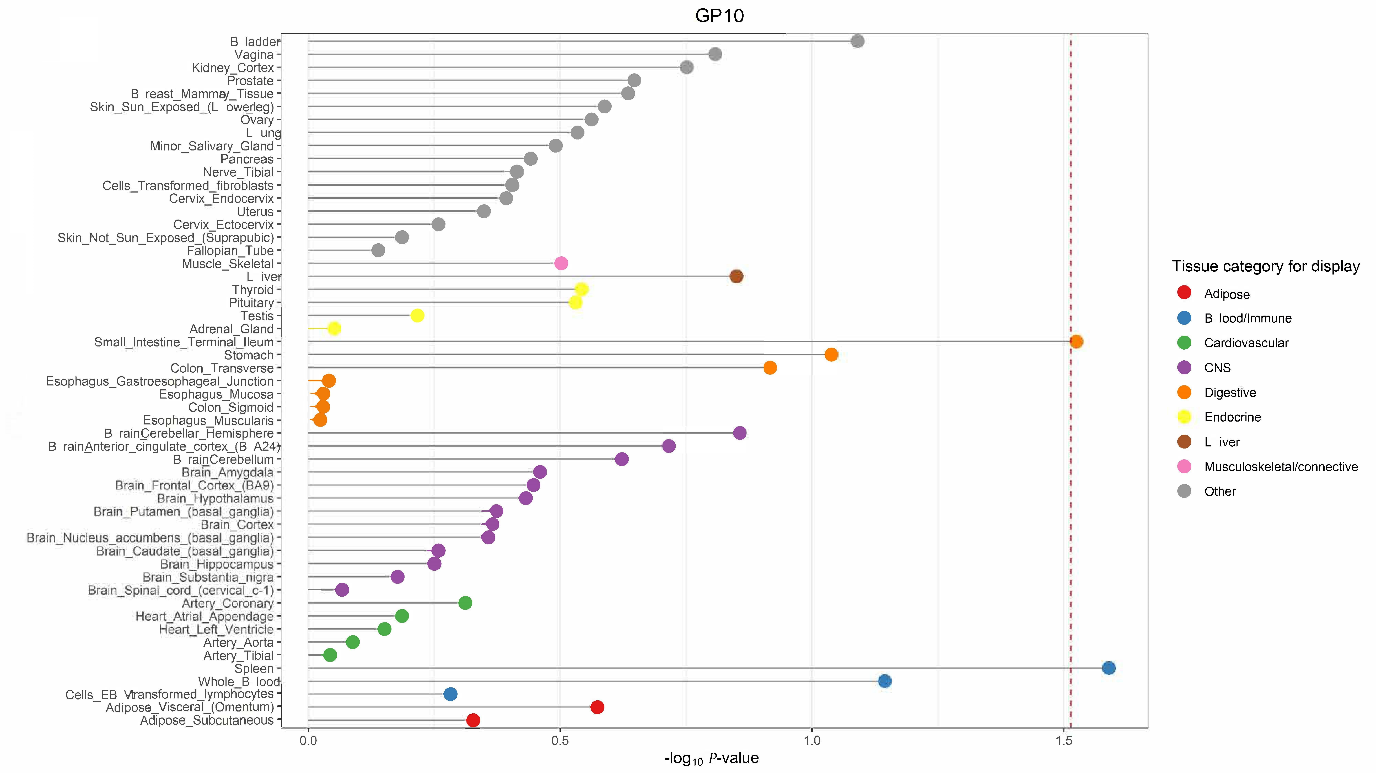


**Supplementary Figure 1.7 |** Results of tissue enrichment for IgG N-GP10. Significantly enriched tissues of IgG N-glycosylation were identified by linkage disequilibrium score regression in a specifically expressed genes (LDSC-SEG) approach. A total of 53 types of tissue obtained from the Genotype-Tissue expression project (GTEx v7) are grouped into nine domains with different colours. Twenty IgG N-glycan peaks (GPs) with significant GWAS results are enriched in all 53 types of tissue. Tissues which are highly enriched for each IgG N-GPs with significant GWAS results across all tissue types in GTEx v7 are shown with a false discovery rate (FDR) significant at 5% (red dotted line).


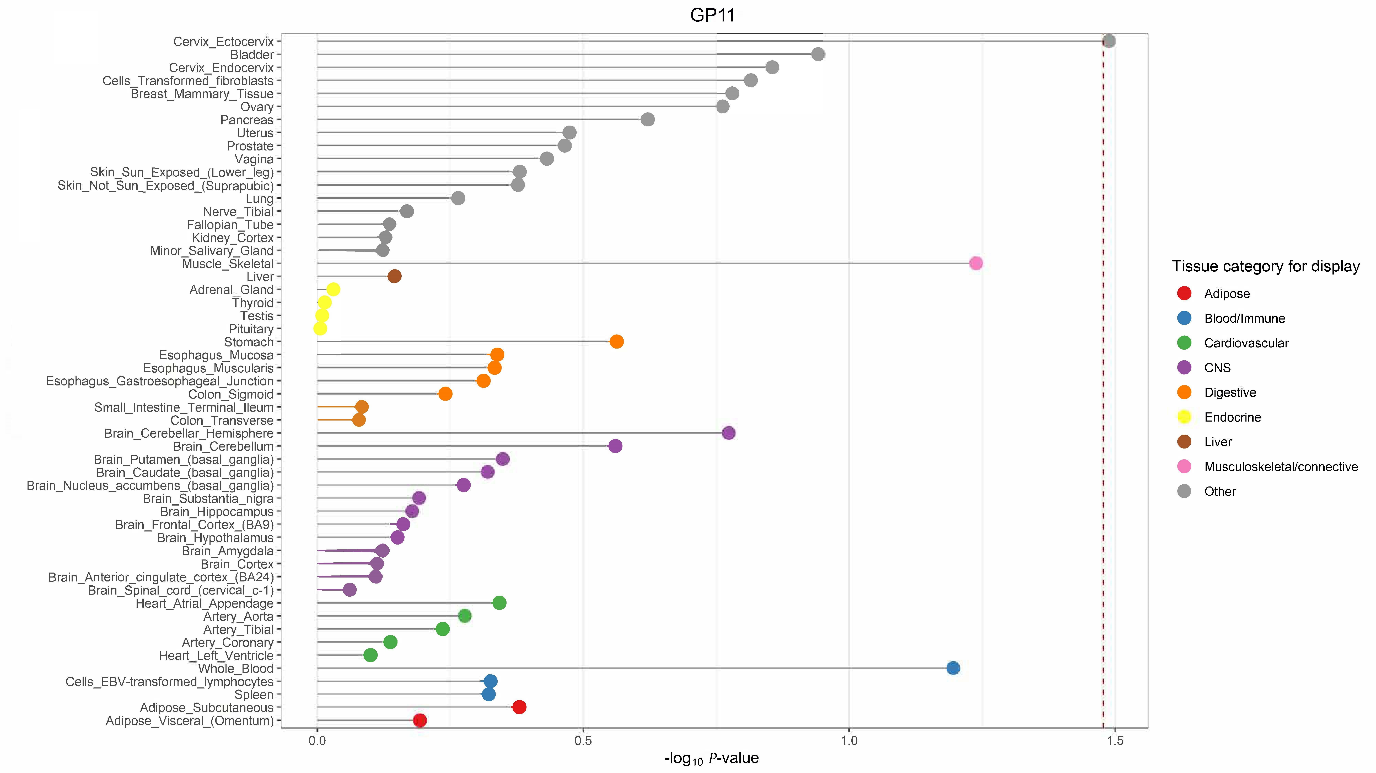


**Supplementary Figure 1.8 |** Results of tissue enrichment for IgG N-GP11. Significantly enriched tissues of IgG N-glycosylation were identified by linkage disequilibrium score regression in a specifically expressed genes (LDSC-SEG) approach. A total of 53 types of tissue obtained from the Genotype-Tissue expression project (GTEx v7) are grouped into nine domains with different colours. Twenty IgG N-glycan peaks (GPs) with significant GWAS results are enriched in all 53 types of tissue. Tissues which are highly enriched for each IgG N-GPs with significant GWAS results across all tissue types in GTEx v7 are shown with a false discovery rate (FDR) significant at 5% (red dotted line).


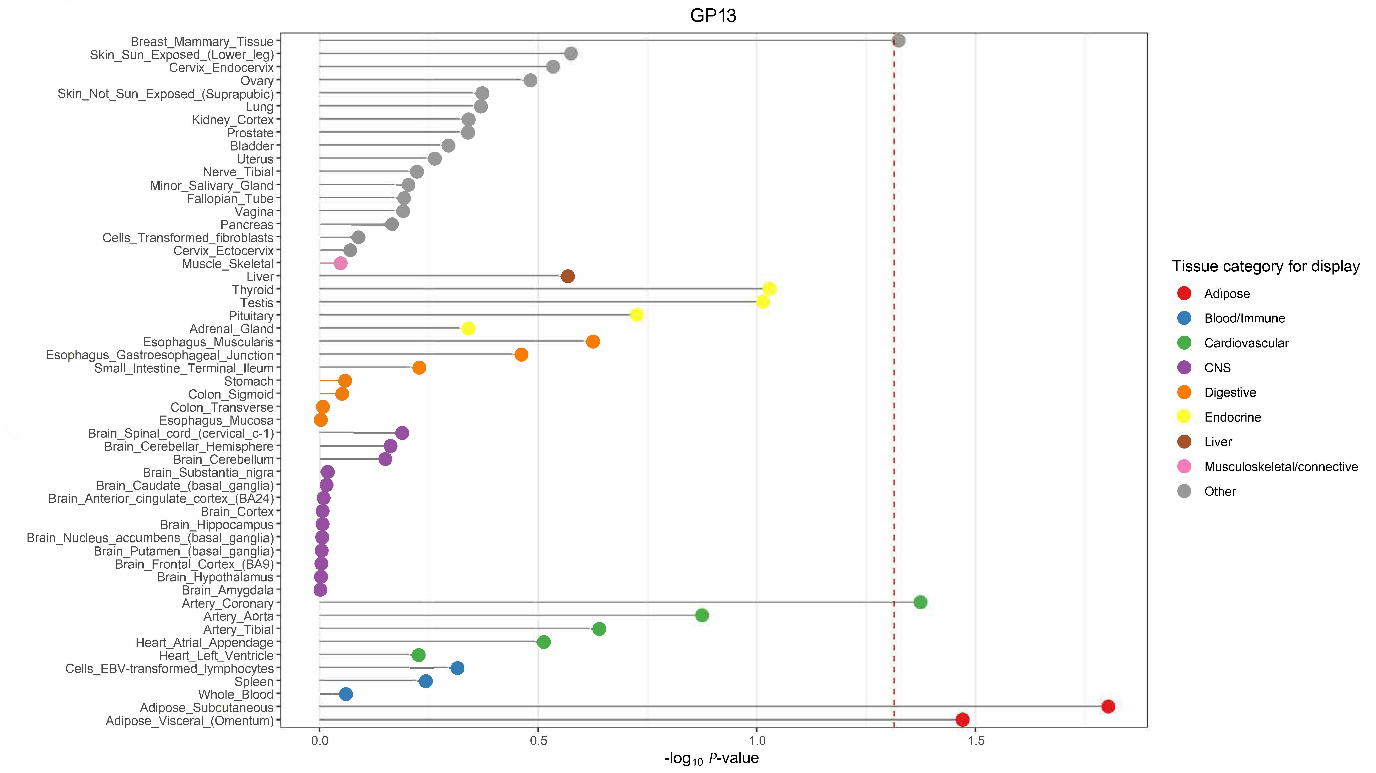


**Supplementary Figure 1.9 |** Results of tissue enrichment for IgG N-GP13. Significantly enriched tissues of IgG N-glycosylation were identified by linkage disequilibrium score regression in a specifically expressed genes (LDSC-SEG) approach. A total of 53 types of tissue obtained from the Genotype-Tissue expression project (GTEx v7) are grouped into nine domains with different colours. Twenty IgG N-glycan peaks (GPs) with significant GWAS results are enriched in all 53 types of tissue. Tissues which are highly enriched for each IgG N-GPs with significant GWAS results across all tissue types in GTEx v7 are shown with a false discovery rate (FDR) significant at 5% (red dotted line).


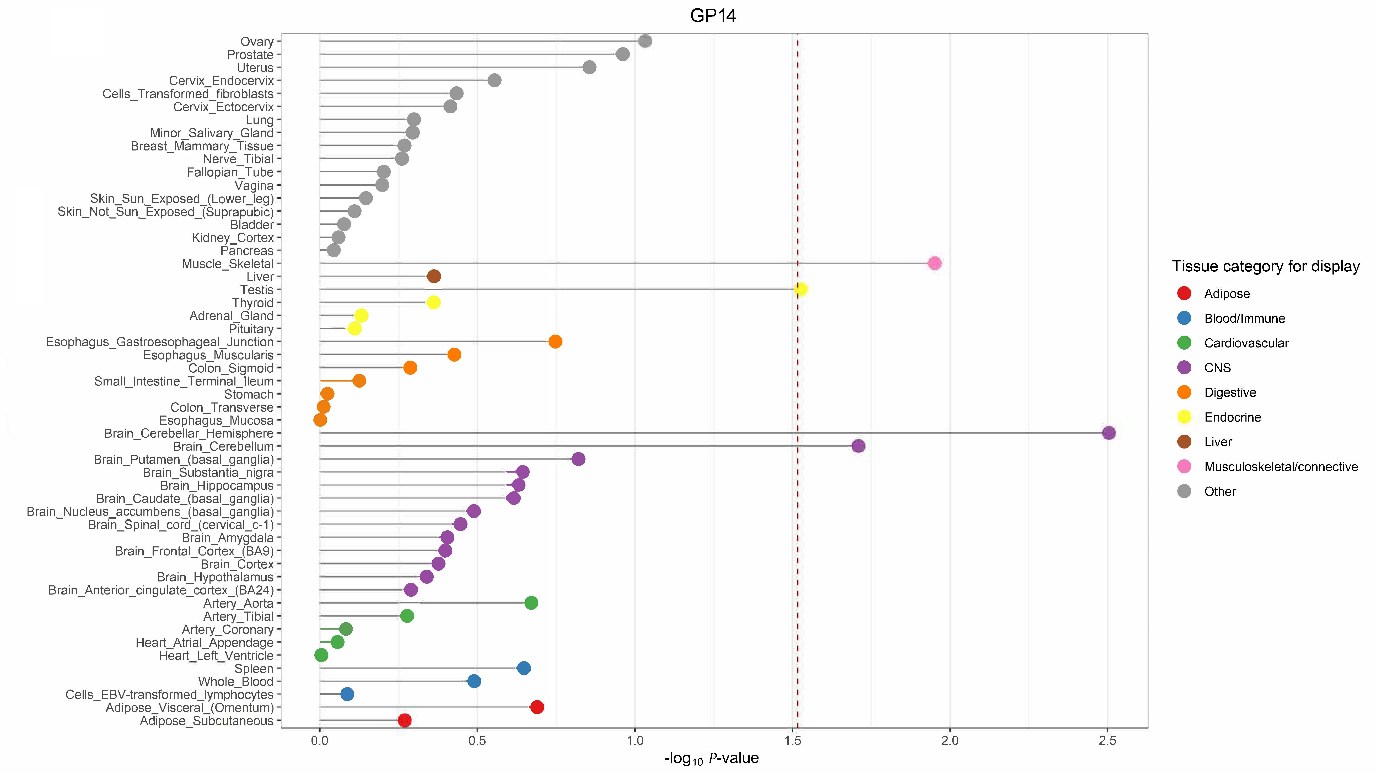


**Supplementary Figure 1.10 |** Results of tissue enrichment for IgG N-GP14. Significantly enriched tissues of IgG N-glycosylation were identified by linkage disequilibrium score regression in a specifically expressed genes (LDSC-SEG) approach. A total of 53 types of tissue obtained from the Genotype-Tissue expression project (GTEx v7) are grouped into nine domains with different colours. Twenty IgG N-glycan peaks (GPs) with significant GWAS results are enriched in all 53 types of tissue. Tissues which are highly enriched for each IgG N-GPs with significant GWAS results across all tissue types in GTEx v7 are shown with a false discovery rate (FDR) significant at 5% (red dotted line).


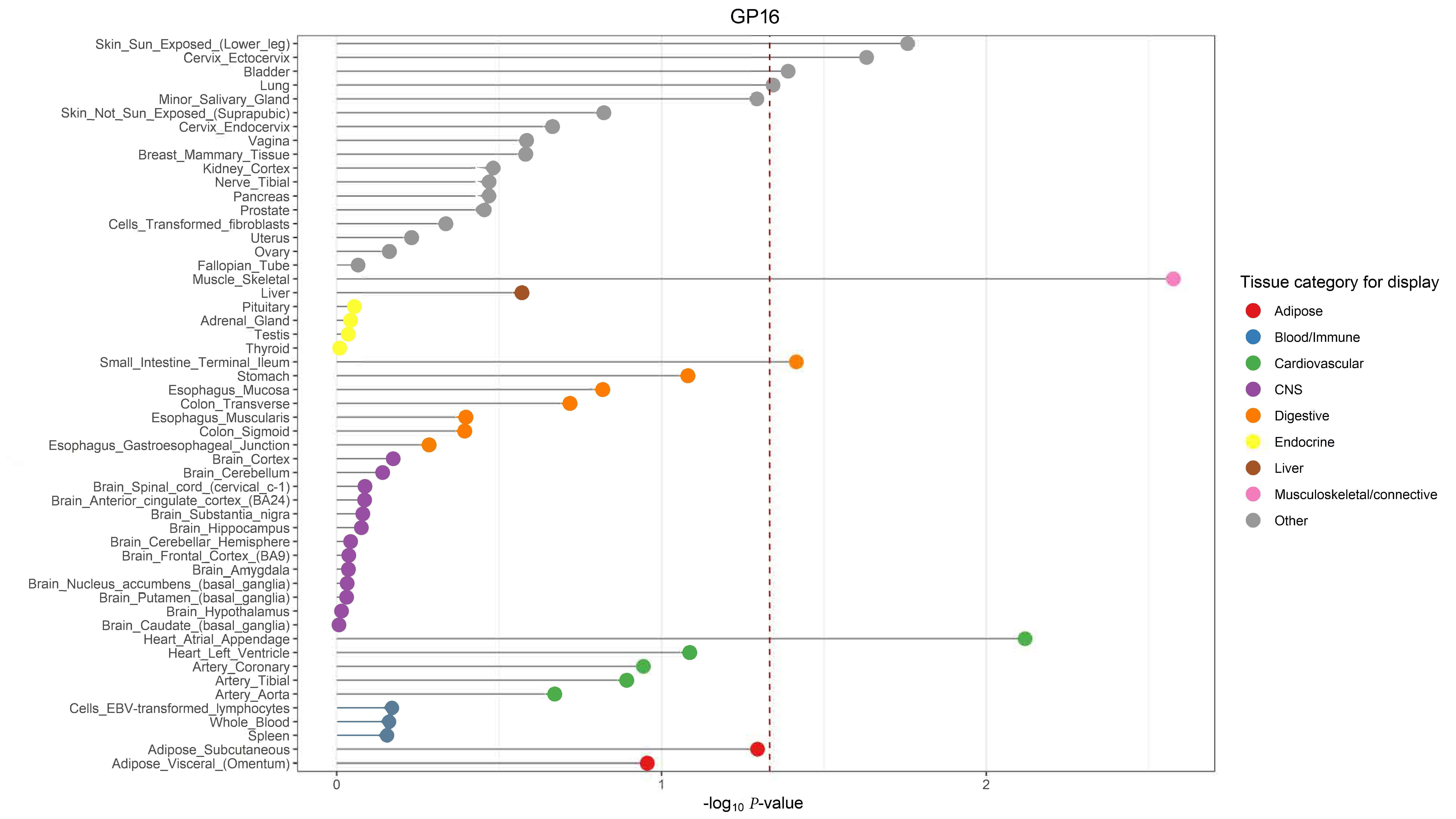


**Supplementary Figure 1.11 |** Results of tissue enrichment for IgG N-GP16. Significantly enriched tissues of IgG N-glycosylation were identified by linkage disequilibrium score regression in a specifically expressed genes (LDSC-SEG) approach. A total of 53 types of tissue obtained from the Genotype-Tissue expression project (GTEx v7) are grouped into nine domains with different colours. Twenty IgG N-glycan peaks (GPs) with significant GWAS results are enriched in all 53 types of tissue. Tissues which are highly enriched for each IgG N-GPs with significant GWAS results across all tissue types in GTEx v7 are shown with a false discovery rate (FDR) significant at 5% (red dotted line).


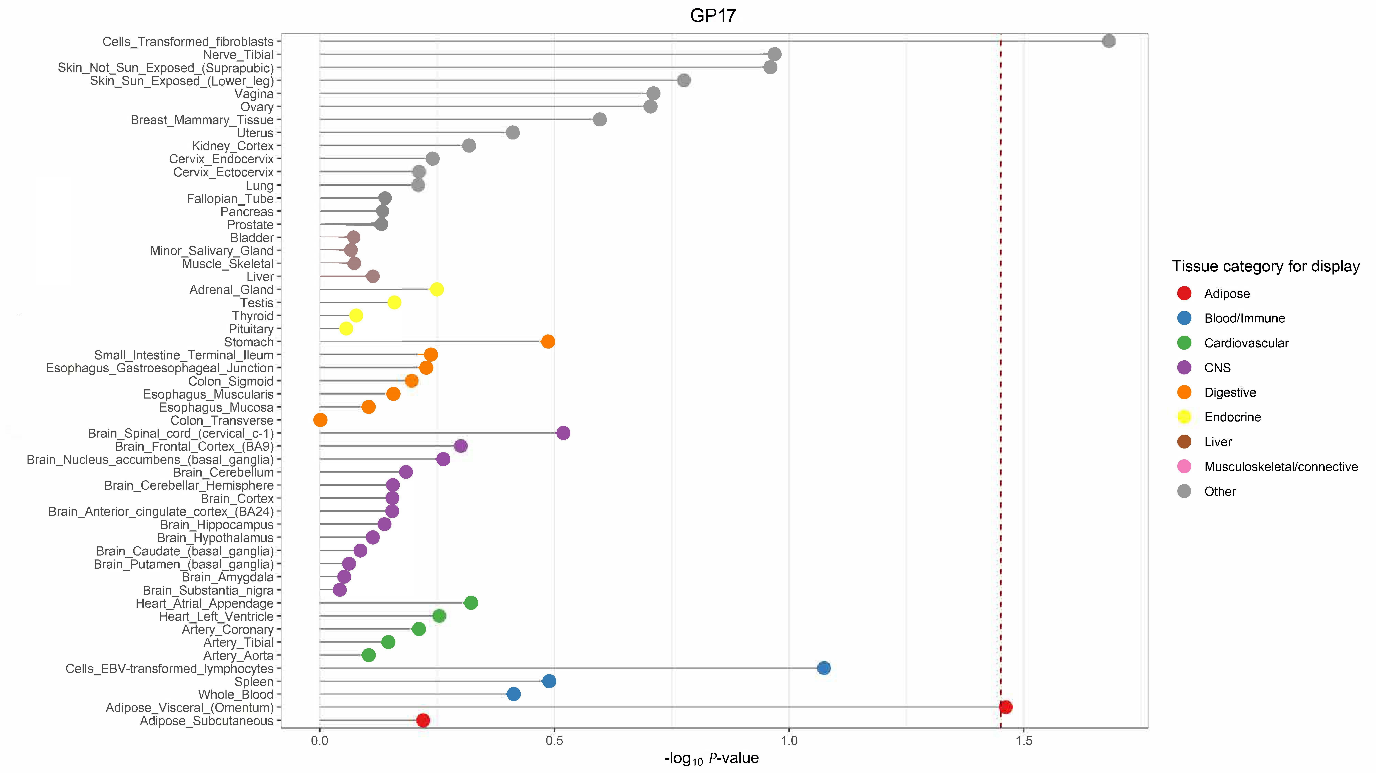


**Supplementary Figure 1.12 |** Results of tissue enrichment for IgG N-GP17. Significantly enriched tissues of IgG N-glycosylation were identified by linkage disequilibrium score regression in a specifically expressed genes (LDSC-SEG) approach. A total of 53 types of tissue obtained from the Genotype-Tissue expression project (GTEx v7) are grouped into nine domains with different colours. Twenty IgG N-glycan peaks (GPs) with significant GWAS results are enriched in all 53 types of tissue. Tissues which are highly enriched for each IgG N-GPs with significant GWAS results across all tissue types in GTEx v7 are shown with a false discovery rate (FDR) significant at 5% (red dotted line).


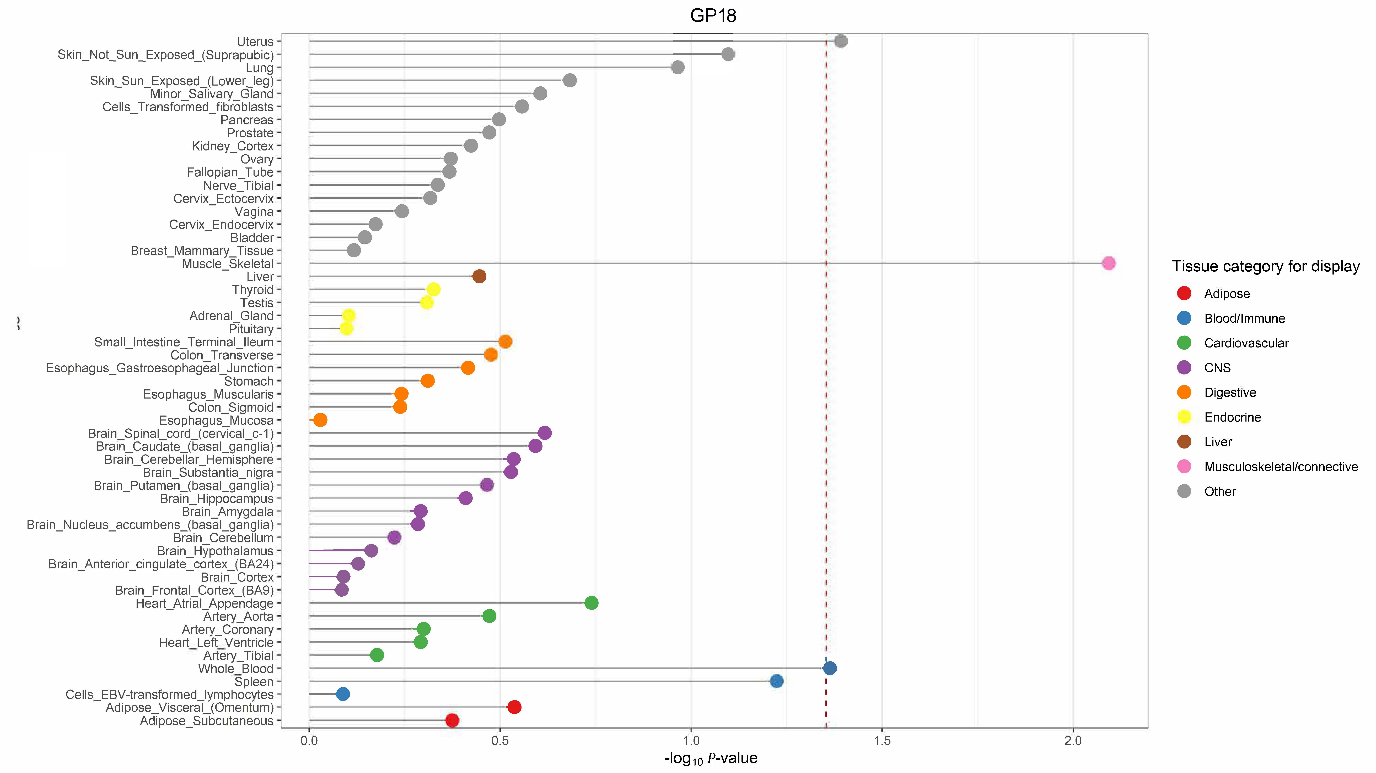


**Supplementary Figure 1.13 |** Results of tissue enrichment for IgG N-GP18. Significantly enriched tissues of IgG N-glycosylation were identified by linkage disequilibrium score regression in a specifically expressed genes (LDSC-SEG) approach. A total of 53 types of tissue obtained from the Genotype-Tissue expression project (GTEx v7) are grouped into nine domains with different colours. Twenty IgG N-glycan peaks (GPs) with significant GWAS results are enriched in all 53 types of tissue. Tissues which are highly enriched for each IgG N-GPs with significant GWAS results across all tissue types in GTEx v7 are shown with a false discovery rate (FDR) significant at 5% (red dotted line).


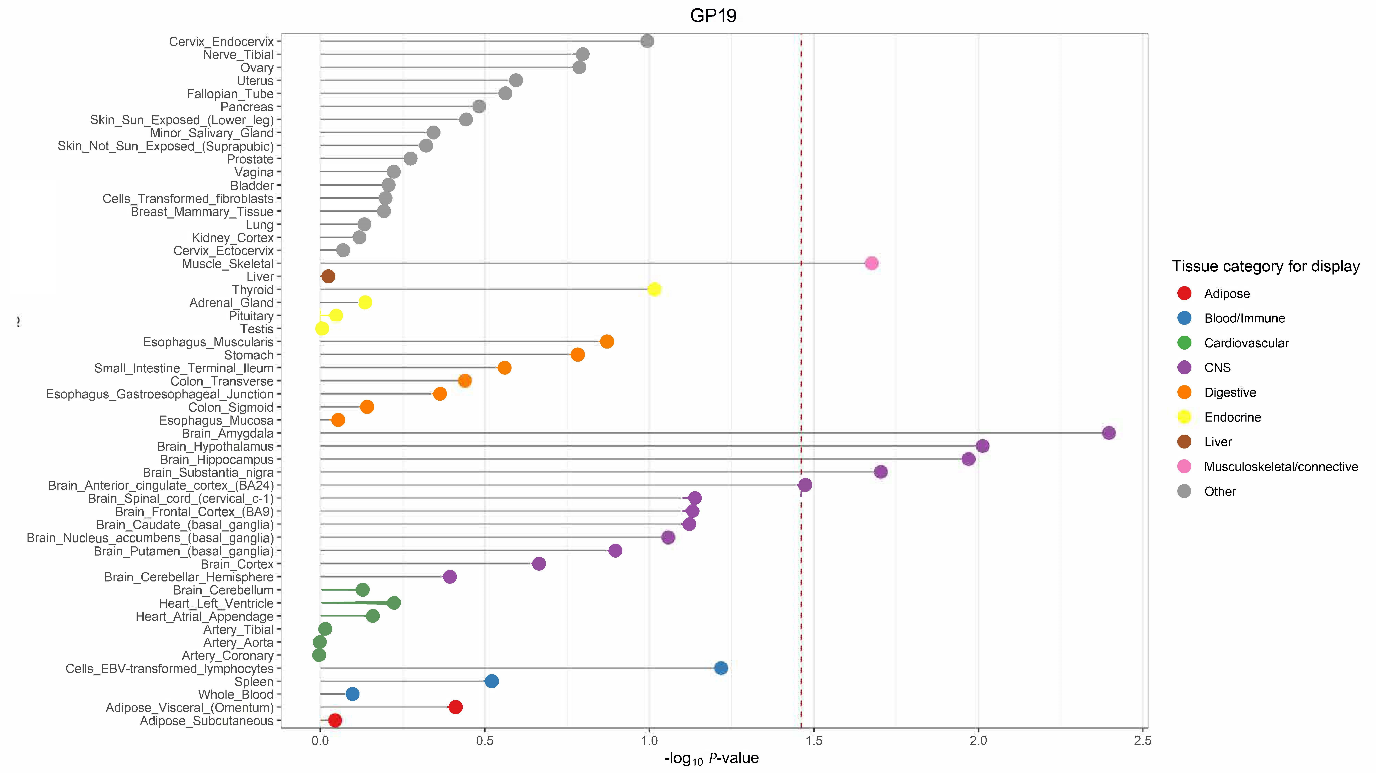


**Supplementary Figure 1.14 |** Results of tissue enrichment for IgG N-GP19. Significantly enriched tissues of IgG N-glycosylation were identified by linkage disequilibrium score regression in a specifically expressed genes (LDSC-SEG) approach. A total of 53 types of tissue obtained from the Genotype-Tissue expression project (GTEx v7) are grouped into nine domains with different colours. Twenty IgG N-glycan peaks (GPs) with significant GWAS results are enriched in all 53 types of tissue. Tissues which are highly enriched for each IgG N-GPs with significant GWAS results across all tissue types in GTEx v7 are shown with a false discovery rate (FDR) significant at 5% (red dotted line).


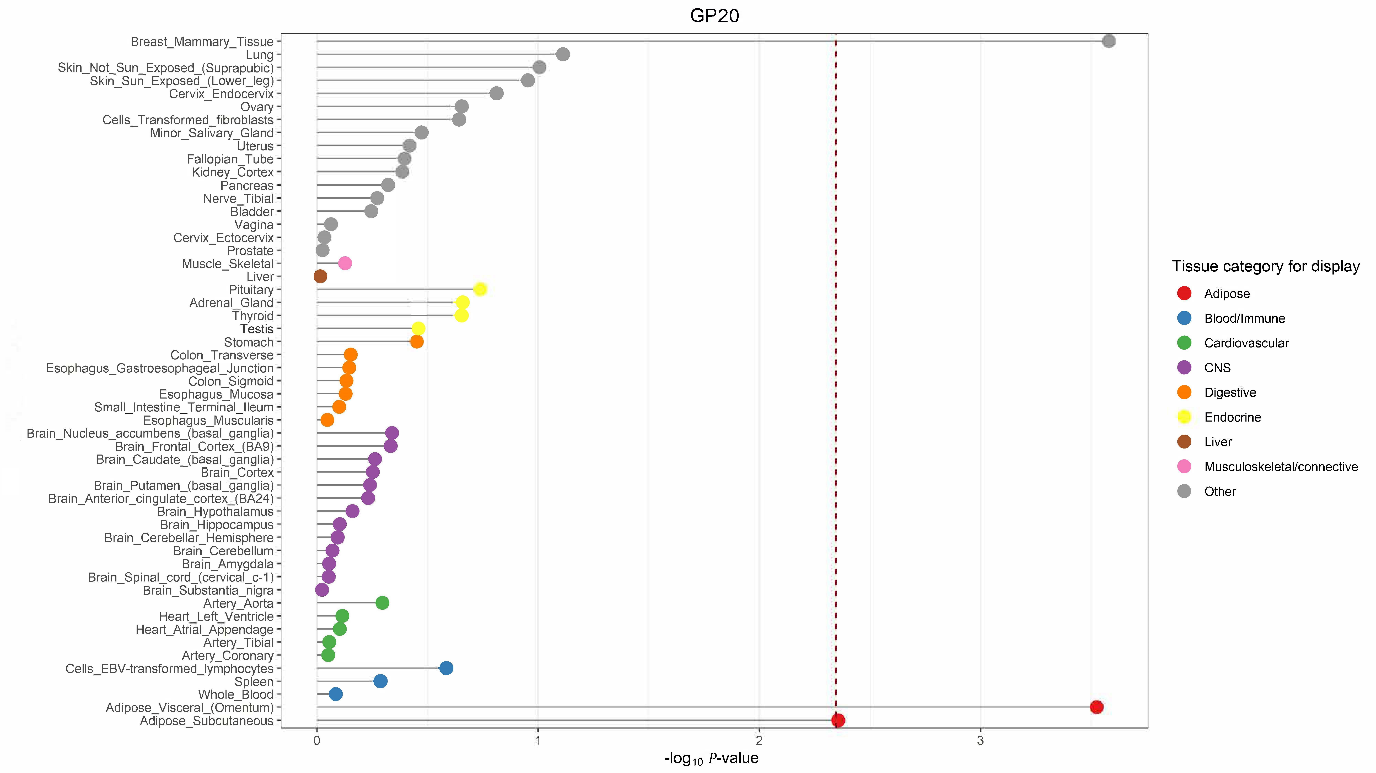


**Supplementary Figure 1.15 |** Results of tissue enrichment for IgG N-GP20. Significantly enriched tissues of IgG N-glycosylation were identified by linkage disequilibrium score regression in a specifically expressed genes (LDSC-SEG) approach. A total of 53 types of tissue obtained from the Genotype-Tissue expression project (GTEx v7) are grouped into nine domains with different colours. Twenty IgG N-glycan peaks (GPs) with significant GWAS results are enriched in all 53 types of tissue. Tissues which are highly enriched for each IgG N-GPs with significant GWAS results across all tissue types in GTEx v7 are shown with a false discovery rate (FDR) significant at 5% (red dotted line).


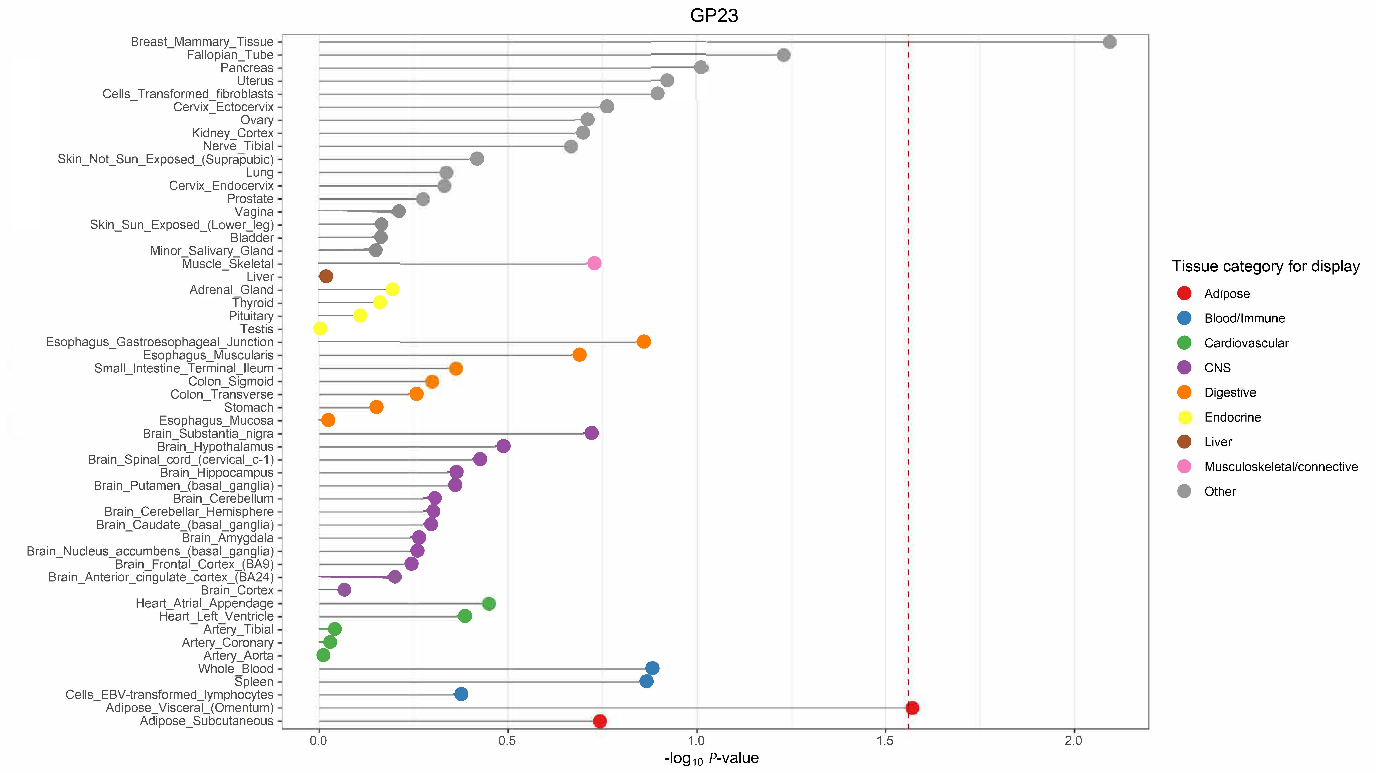


**Supplementary Figure 1.16 |** Results of tissue enrichment for IgG N-GP23. Significantly enriched tissues of IgG N-glycosylation were identified by linkage disequilibrium score regression in a specifically expressed genes (LDSC-SEG) approach. A total of 53 types of tissue obtained from the Genotype-Tissue expression project (GTEx v7) are grouped into nine domains with different colours. Twenty IgG N-glycan peaks (GPs) with significant GWAS results are enriched in all 53 types of tissue. Tissues which are highly enriched for each IgG N-GPs with significant GWAS results across all tissue types in GTEx v7 are shown with a false discovery rate (FDR) significant at 5% (red dotted line).


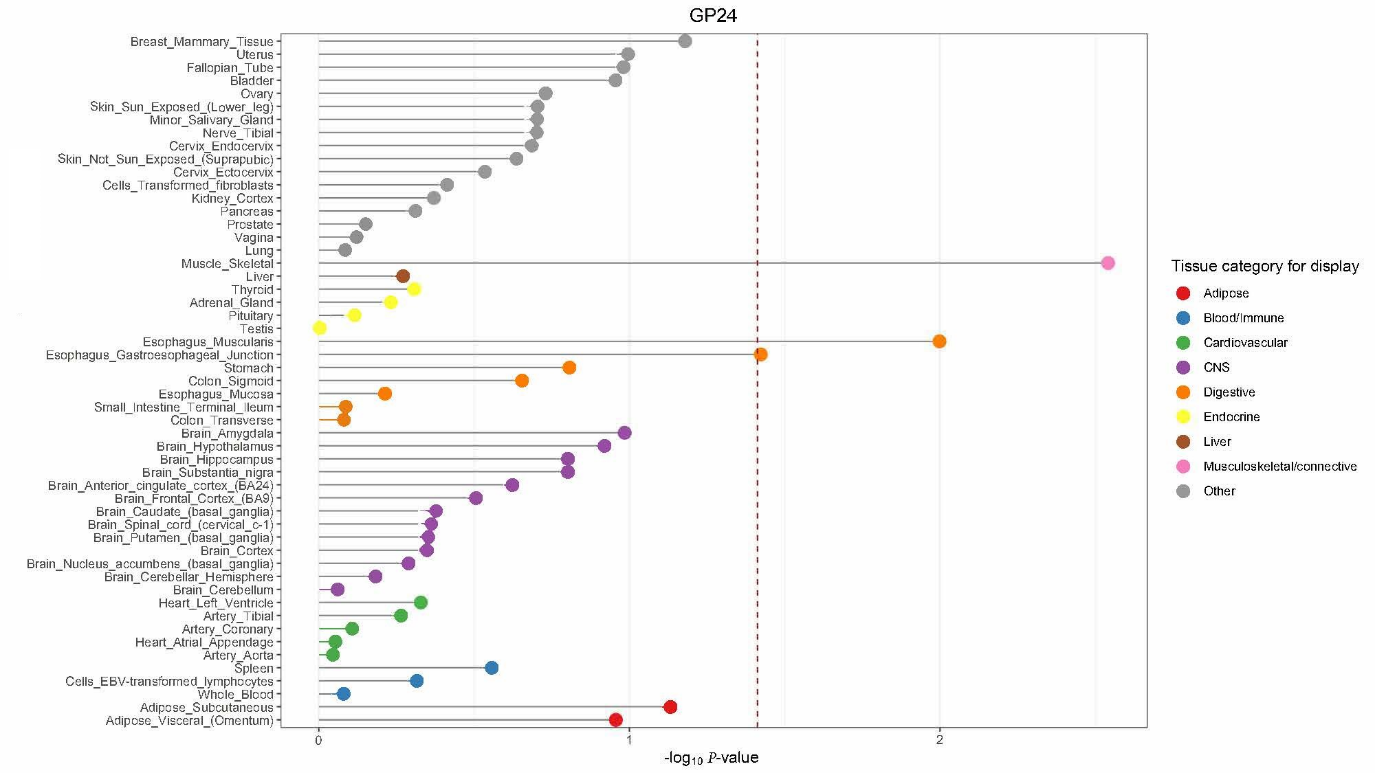


**Supplementary Figure 1.17 |** Results of tissue enrichment for IgG N-GP24. Significantly enriched tissues of IgG N-glycosylation were identified by linkage disequilibrium score regression in a specifically expressed genes (LDSC-SEG) approach. A total of 53 types of tissue obtained from the Genotype-Tissue expression project (GTEx v7) are grouped into nine domains with different colours. Twenty IgG N-glycan peaks (GPs) with significant GWAS results are enriched in all 53 types of tissue. Tissues which are highly enriched for each IgG N-GPs with significant GWAS results across all tissue types in GTEx v7 are shown with a false discovery rate (FDR) significant at 5% (red dotted line).


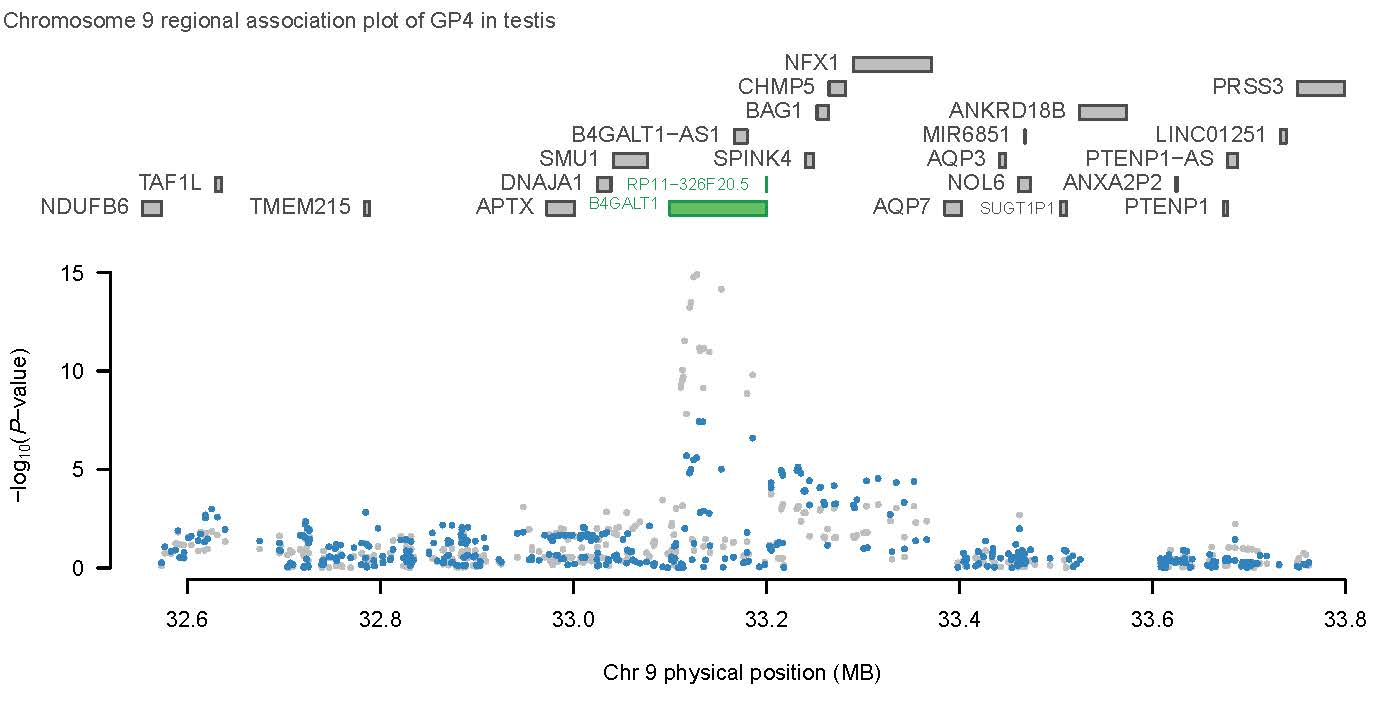


**Supplementary Figure 2.1 |** Regional association of TWAS hits reported by previous GWAS. The top panel in each plot highlights all genes in this 1 Mb window. The marginally significant genes identified by TWAS are coloured in orange, and the jointly significant genes are highlighted in green. The bottom panel shows a Manhattan plot of the GWAS data before (grey) and after (blue) conditioning on the predicted expression of the green genes.


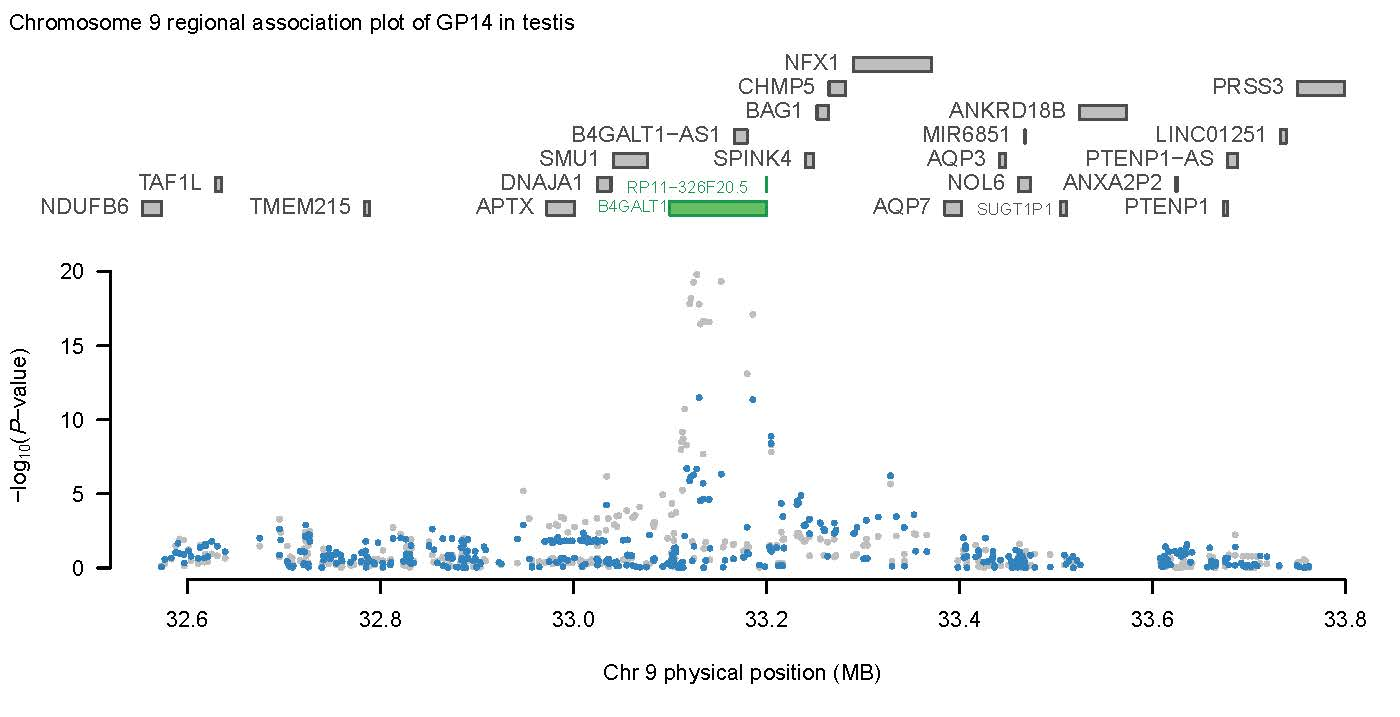


**Supplementary Figure 2.2 |** Regional association of TWAS hits reported by previous GWAS. The top panel in each plot highlights all genes in this 1 Mb window. The marginally significant genes identified by TWAS are coloured in orange, and the jointly significant genes are highlighted in green. The bottom panel shows a Manhattan plot of the GWAS data before (grey) and after (blue) conditioning on the predicted expression of the green genes.


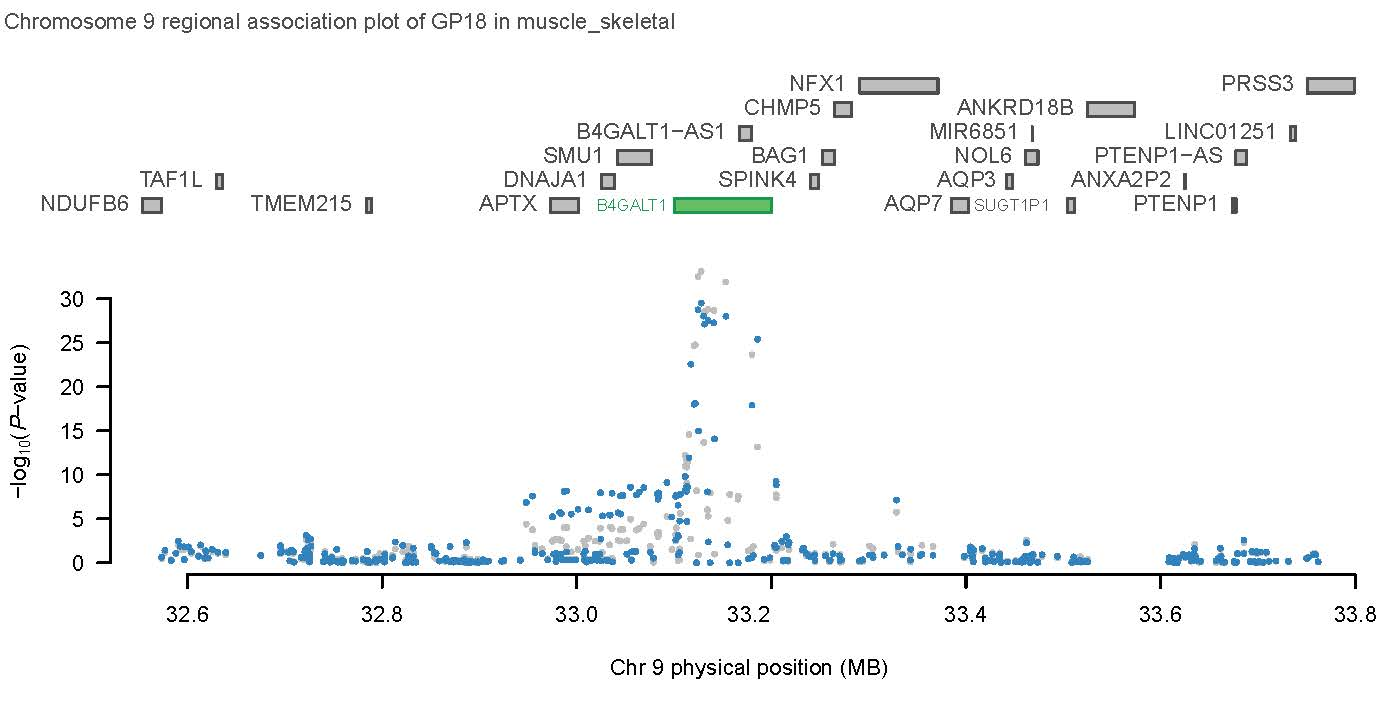


**Supplementary Figure 2.3 |** Regional association of TWAS hits reported by previous GWAS. The top panel in each plot highlights all genes in this 1 Mb window. The marginally significant genes identified by TWAS are coloured in orange, and the jointly significant genes are highlighted in green. The bottom panel shows a Manhattan plot of the GWAS data before (grey) and after (blue) conditioning on the predicted expression of the green genes.


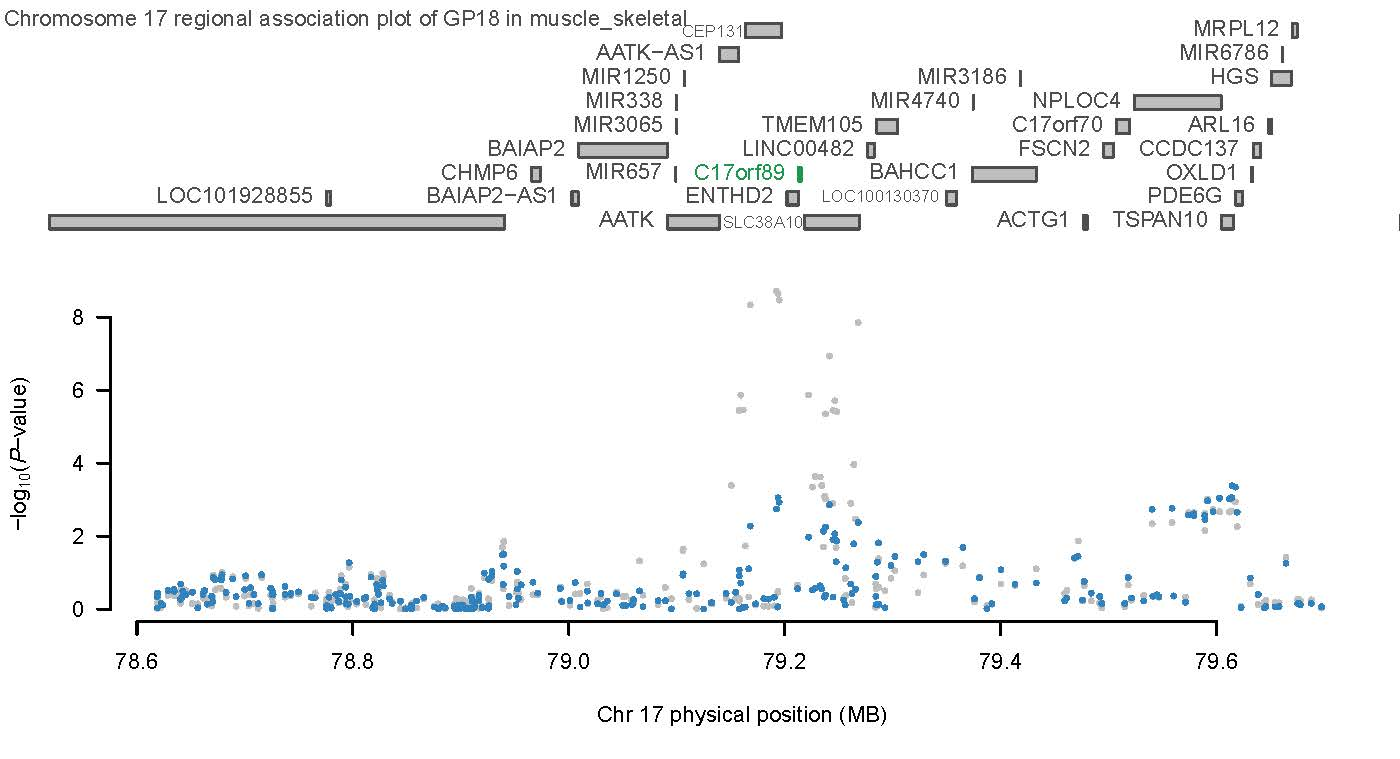


**Supplementary Figure 2.4 |** Regional association of TWAS hits reported by previous GWAS. The top panel in each plot highlights all genes in this 1 Mb window. The marginally significant genes identified by TWAS are coloured in orange, and the jointly significant genes are highlighted in green. The bottom panel shows a Manhattan plot of the GWAS data before (grey) and after (blue) conditioning on the predicted expression of the green genes.


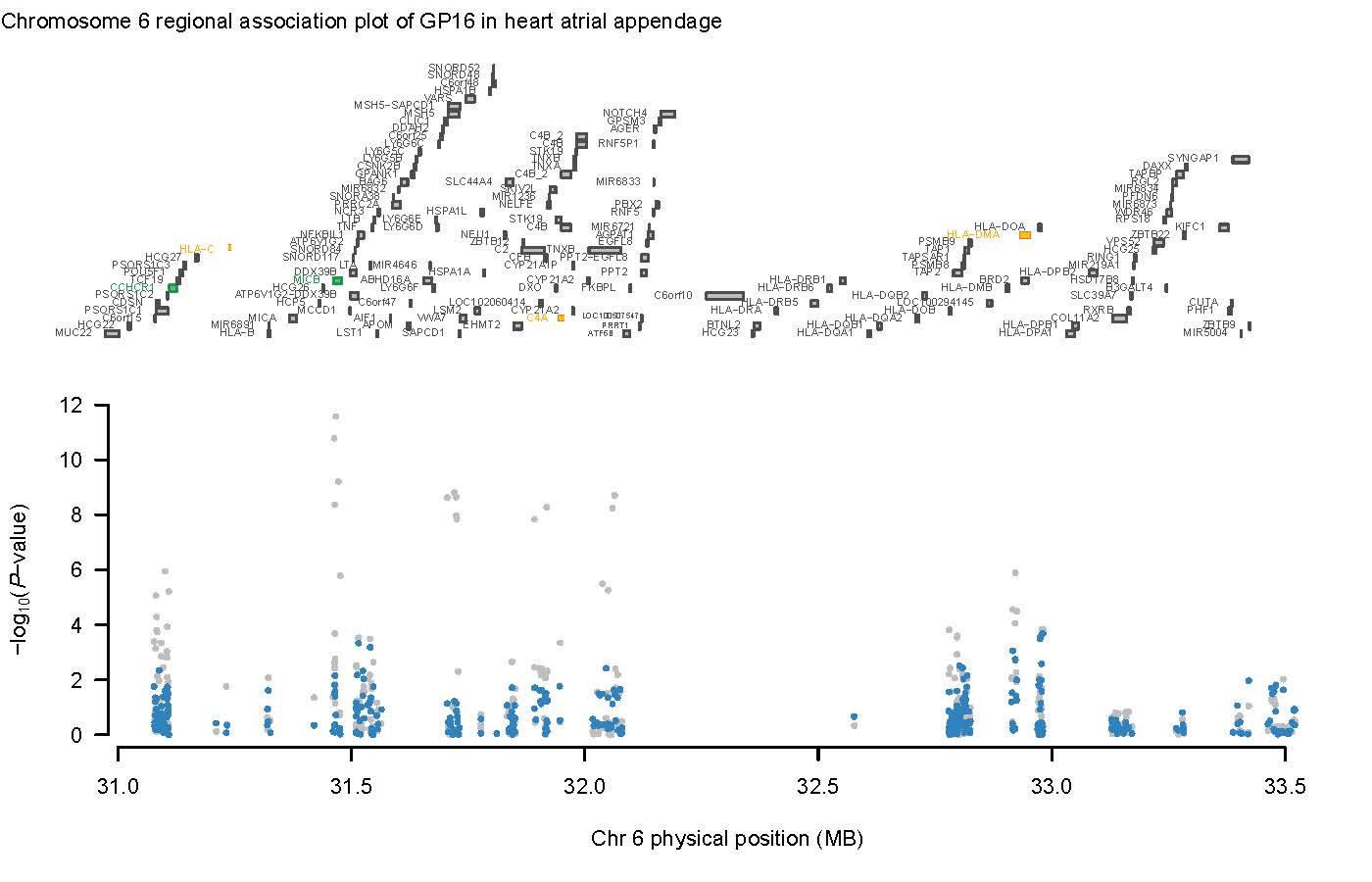


**Supplementary Figure 2.5 |** Regional association of TWAS hits reported by previous GWAS. The top panel in each plot highlights all genes in this 1 Mb window. The marginally significant genes identified by TWAS are coloured in orange, and the jointly significant genes are highlighted in green. The bottom panel shows a Manhattan plot of the GWAS data before (grey) and after (blue) conditioning on the predicted expression of the green genes.


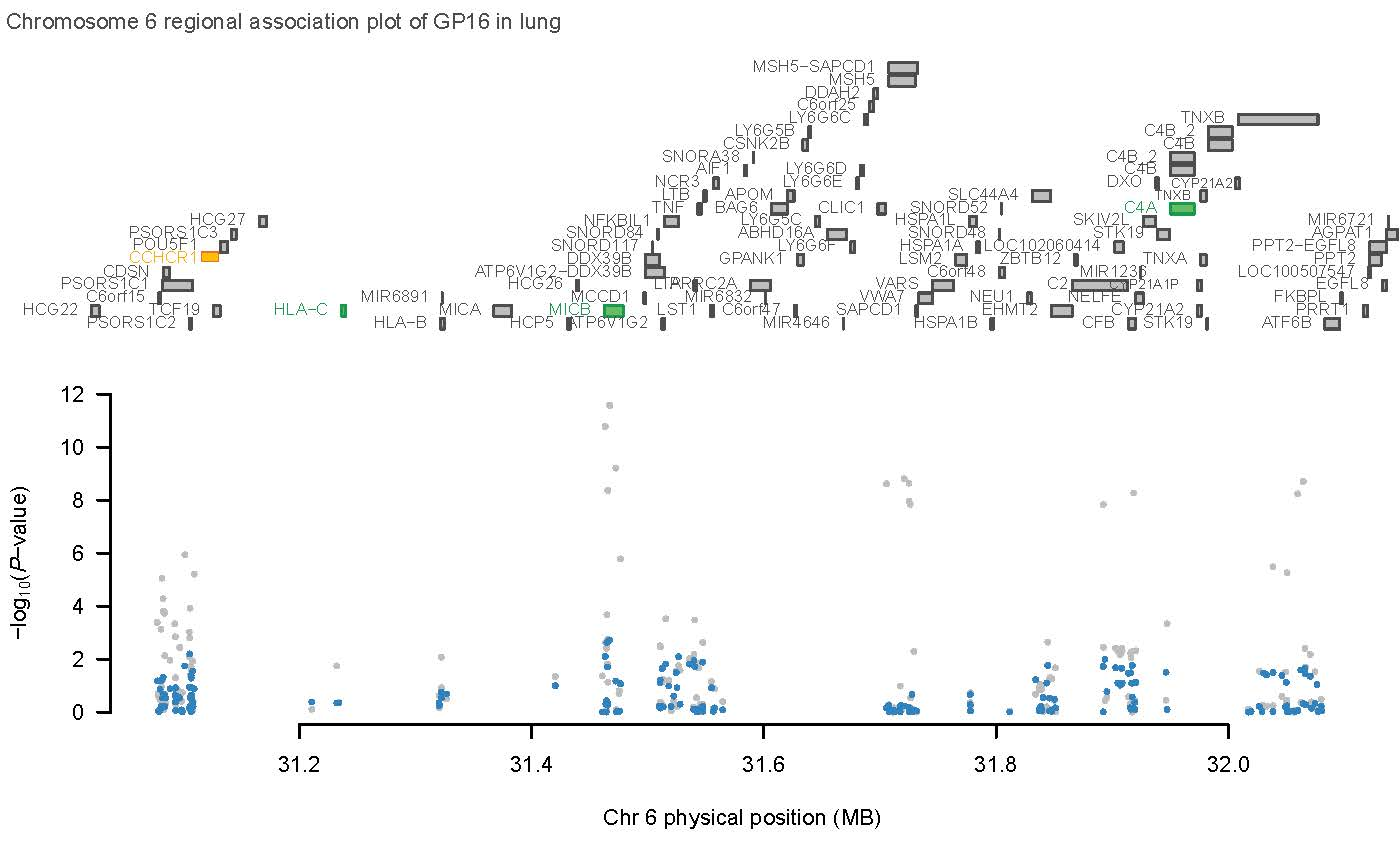


**Supplementary Figure 2.6 |** Regional association of TWAS hits reported by previous GWAS. The top panel in each plot highlights all genes in this 1 Mb window. The marginally significant genes identified by TWAS are coloured in orange, and the jointly significant genes are highlighted in green. The bottom panel shows a Manhattan plot of the GWAS data before (grey) and after (blue) conditioning on the predicted expression of the green genes.


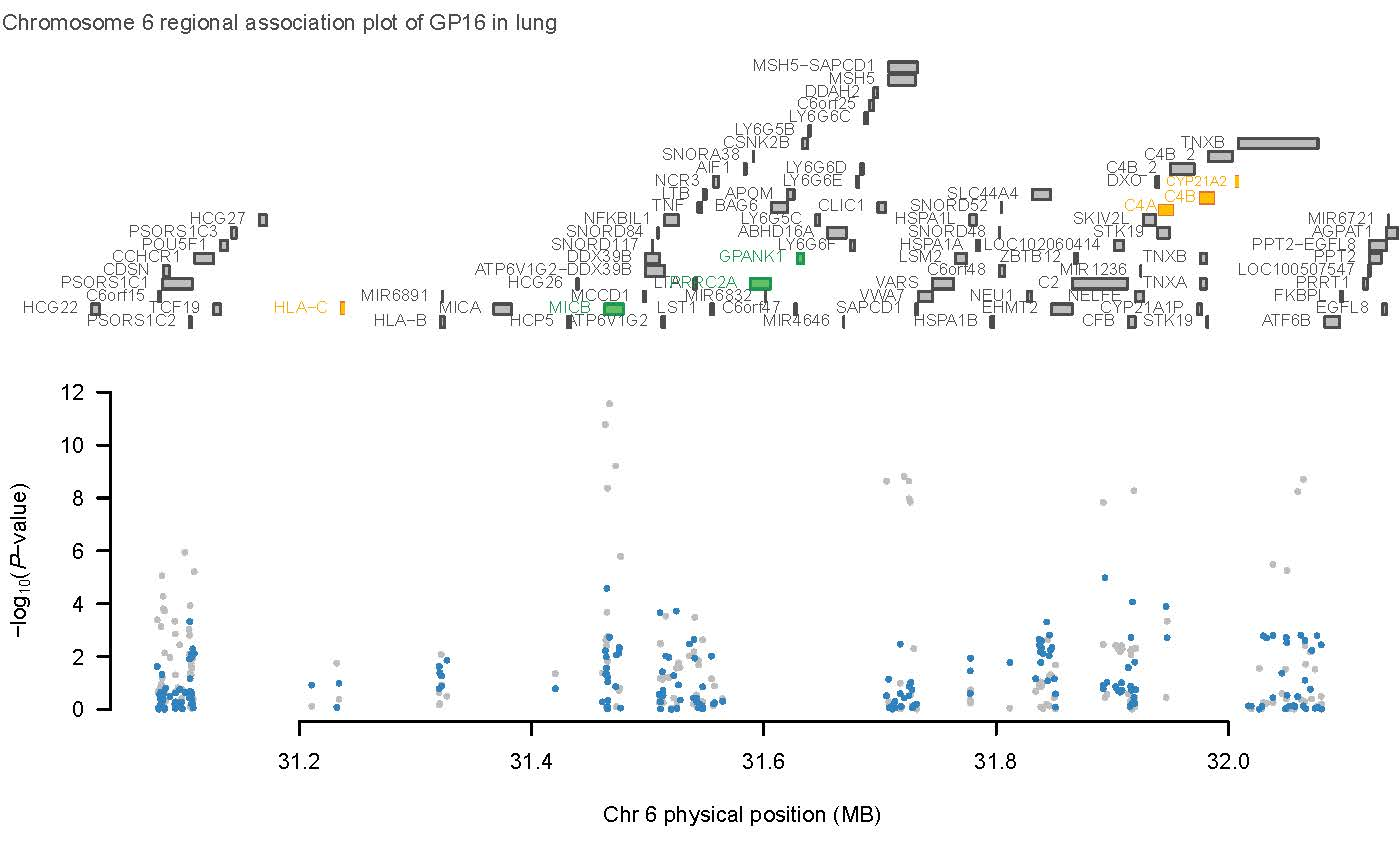


**Supplementary Figure 2.7 |** Regional association of TWAS hits reported by previous GWAS. The top panel in each plot highlights all genes in this 1 Mb window. The marginally significant genes identified by TWAS are coloured in orange, and the jointly significant genes are highlighted in green. The bottom panel shows a Manhattan plot of the GWAS data before (grey) and after (blue) conditioning on the predicted expression of the green genes.


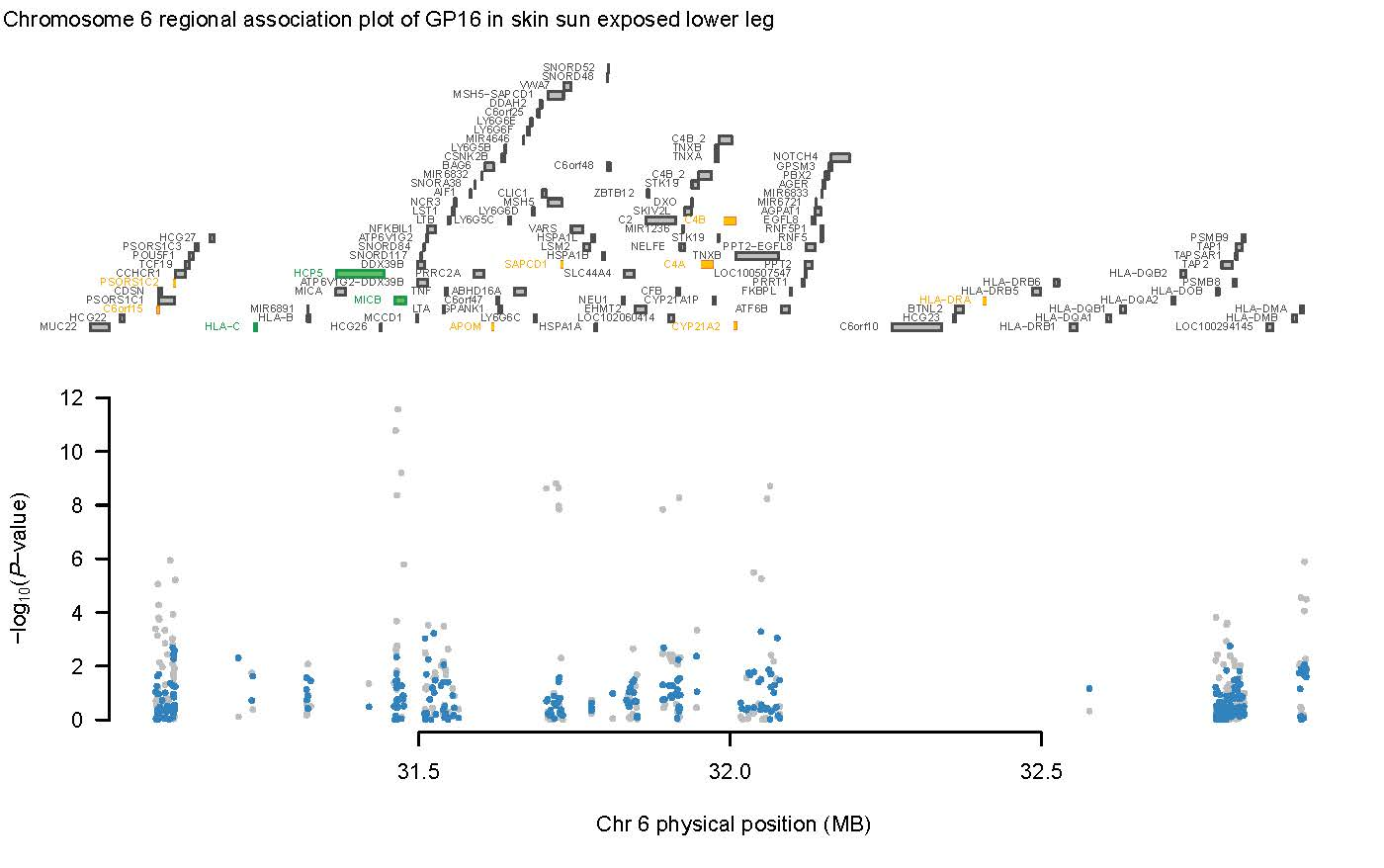


**Supplementary Figure 2.8 |** Regional association of TWAS hits reported by previous GWAS. The top panel in each plot highlights all genes in this 1 Mb window. The marginally significant genes identified by TWAS are coloured in orange, and the jointly significant genes are highlighted in green. The bottom panel shows a Manhattan plot of the GWAS data before (grey) and after (blue) conditioning on the predicted expression of the green genes.


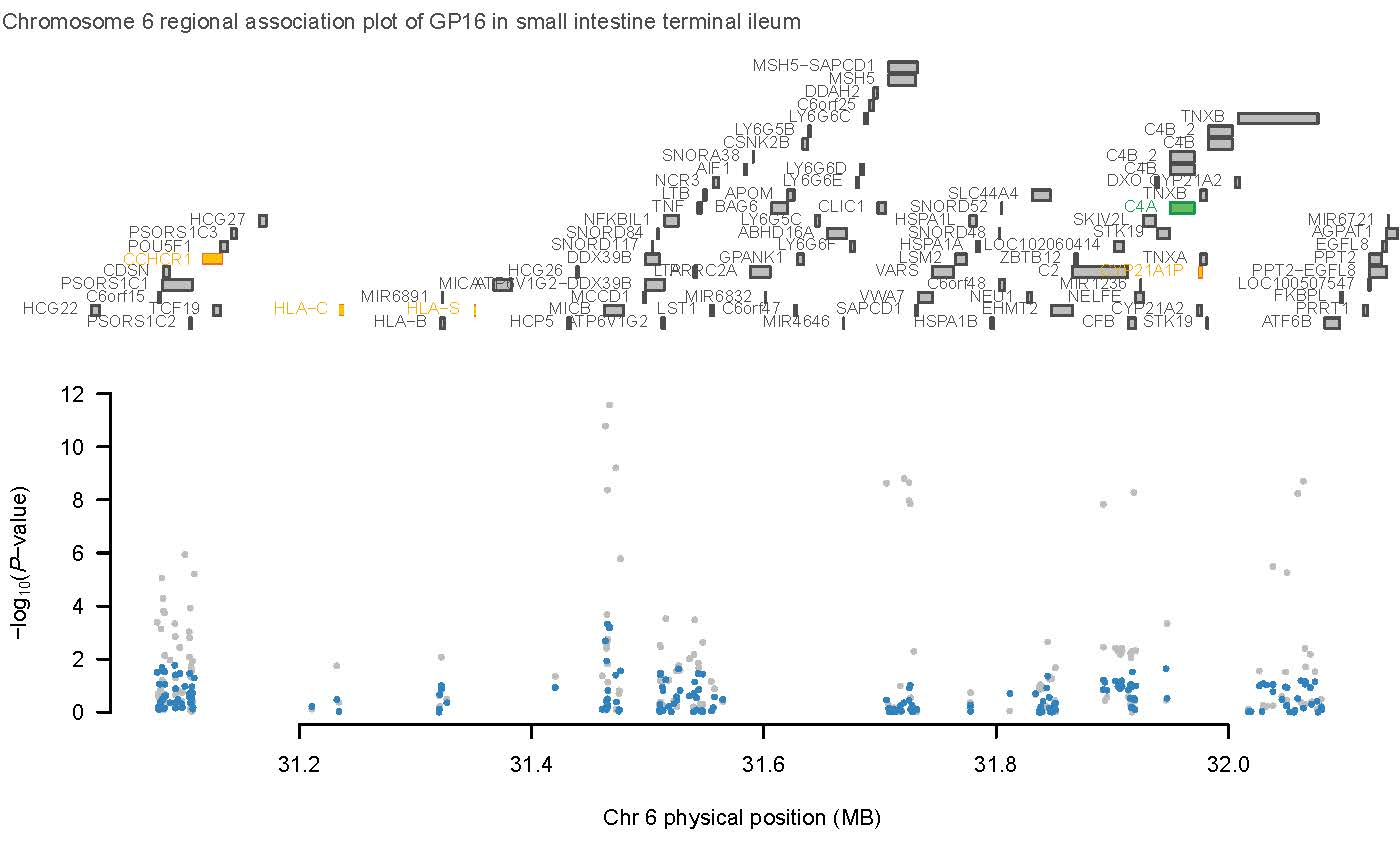


**Supplementary Figure 2.9 |** Regional association of TWAS hits reported by previous GWAS. The top panel in each plot highlights all genes in this 1 Mb window. The marginally significant genes identified by TWAS are coloured in orange, and the jointly significant genes are highlighted in green. The bottom panel shows a Manhattan plot of the GWAS data before (grey) and after (blue) conditioning on the predicted expression of the green genes.


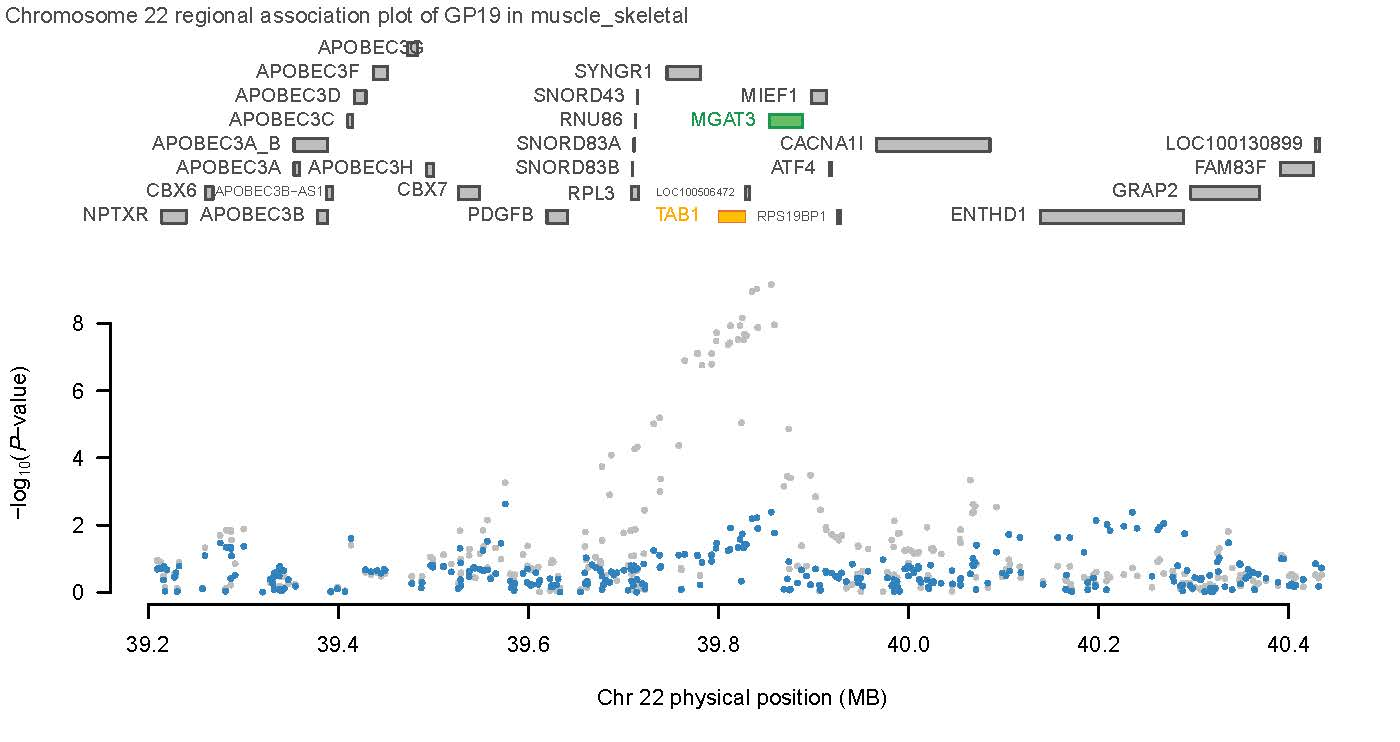


**Supplementary Figure 2.10 |** Regional association of TWAS hits reported by previous GWAS. The top panel in each plot highlights all genes in this 1 Mb window. The marginally significant genes identified by TWAS are coloured in orange, and the jointly significant genes are highlighted in green. The bottom panel shows a Manhattan plot of the GWAS data before (grey) and after (blue) conditioning on the predicted expression of the green genes.


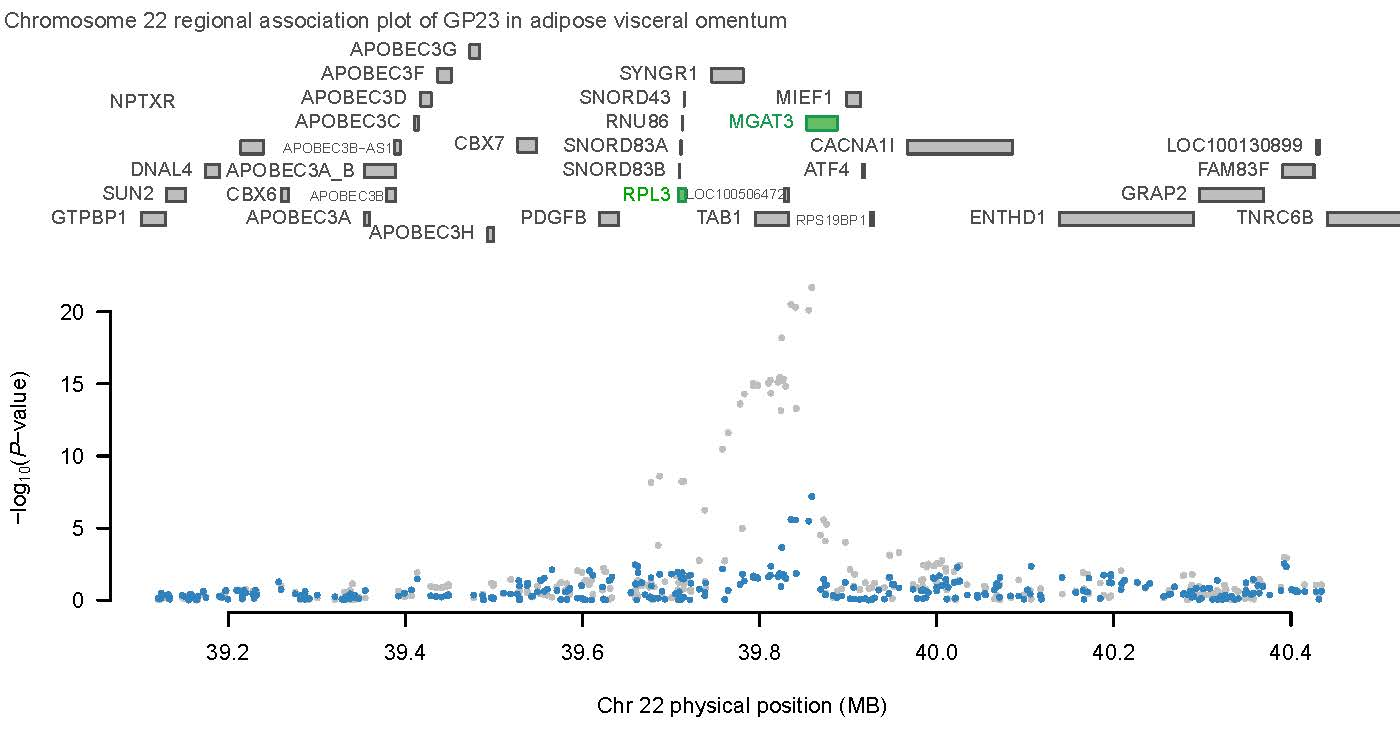


**Supplementary Figure 2.11 |** Regional association of TWAS hits reported by previous GWAS. The top panel in each plot highlights all genes in this 1 Mb window. The marginally significant genes identified by TWAS are coloured in orange, and the jointly significant genes are highlighted in green. The bottom panel shows a Manhattan plot of the GWAS data before (grey) and after (blue) conditioning on the predicted expression of the green genes.


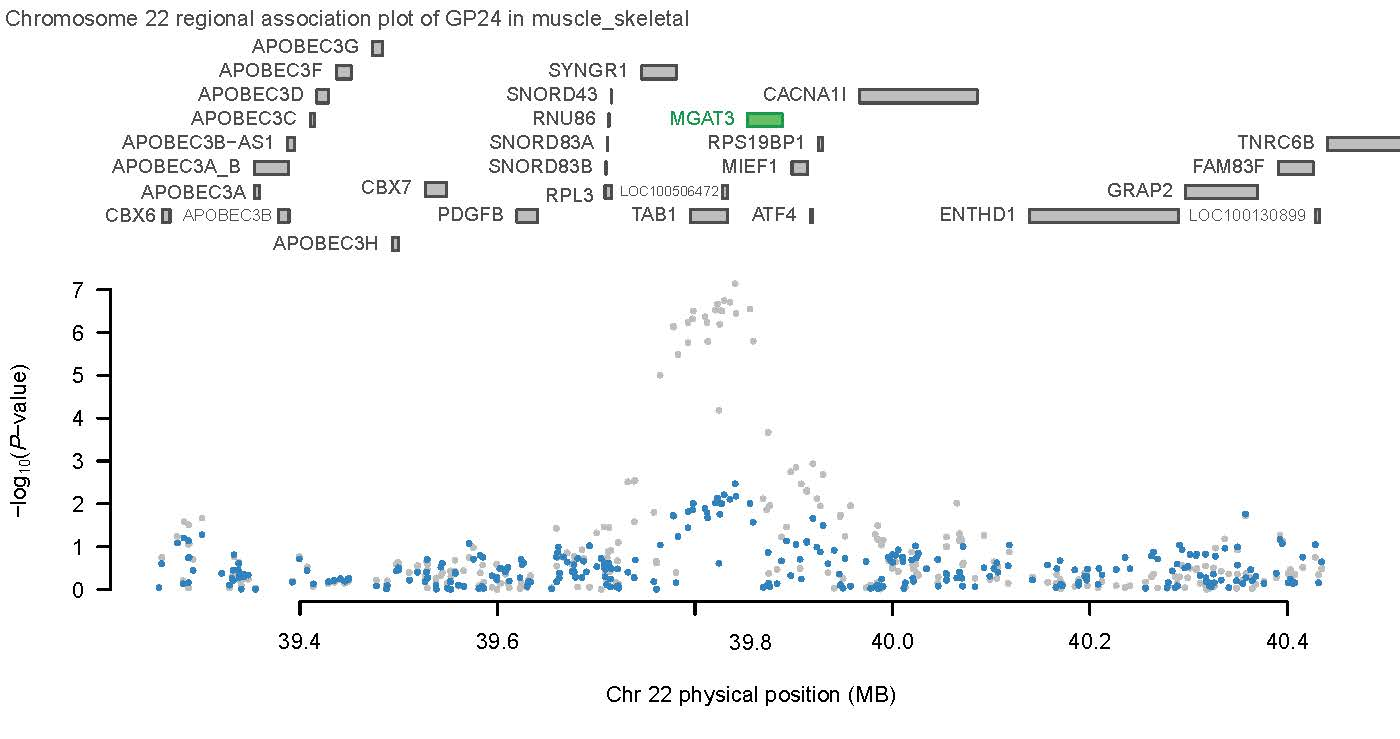


**Supplementary Figure 2.12 |** Regional association of TWAS hits reported by previous GWAS. The top panel in each plot highlights all genes in this 1 Mb window. The marginally significant genes identified by TWAS are coloured in orange, and the jointly significant genes are highlighted in green. The bottom panel shows a Manhattan plot of the GWAS data before (grey) and after (blue) conditioning on the predicted expression of the green genes.


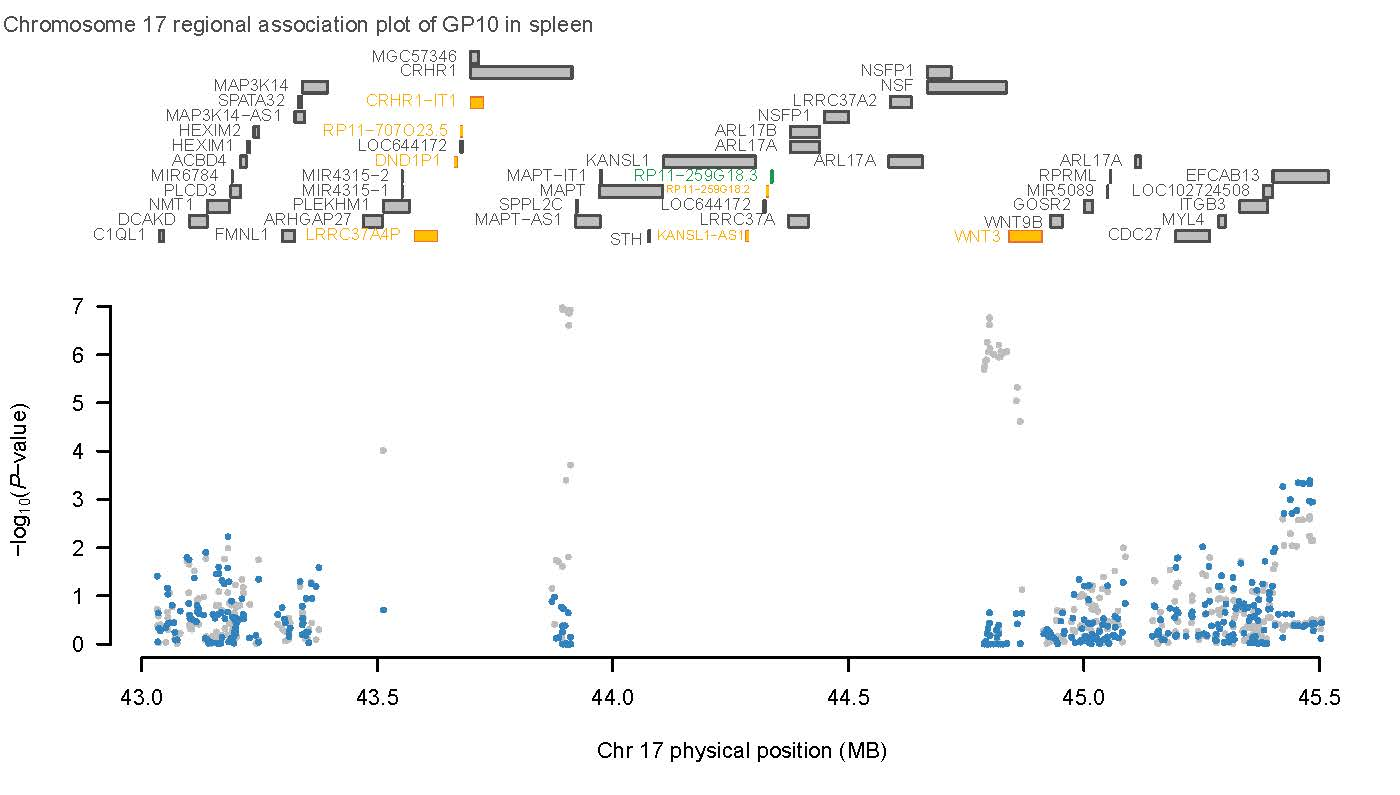


**Supplementary Figure 2.13 |** Regional association of TWAS hits reported by previous GWAS. The top panel in each plot highlights all genes in this 1 Mb window. The marginally significant genes identified by TWAS are coloured in orange, and the jointly significant genes are highlighted in green. The bottom panel shows a Manhattan plot of the GWAS data before (grey) and after (blue) conditioning on the predicted expression of the green genes.


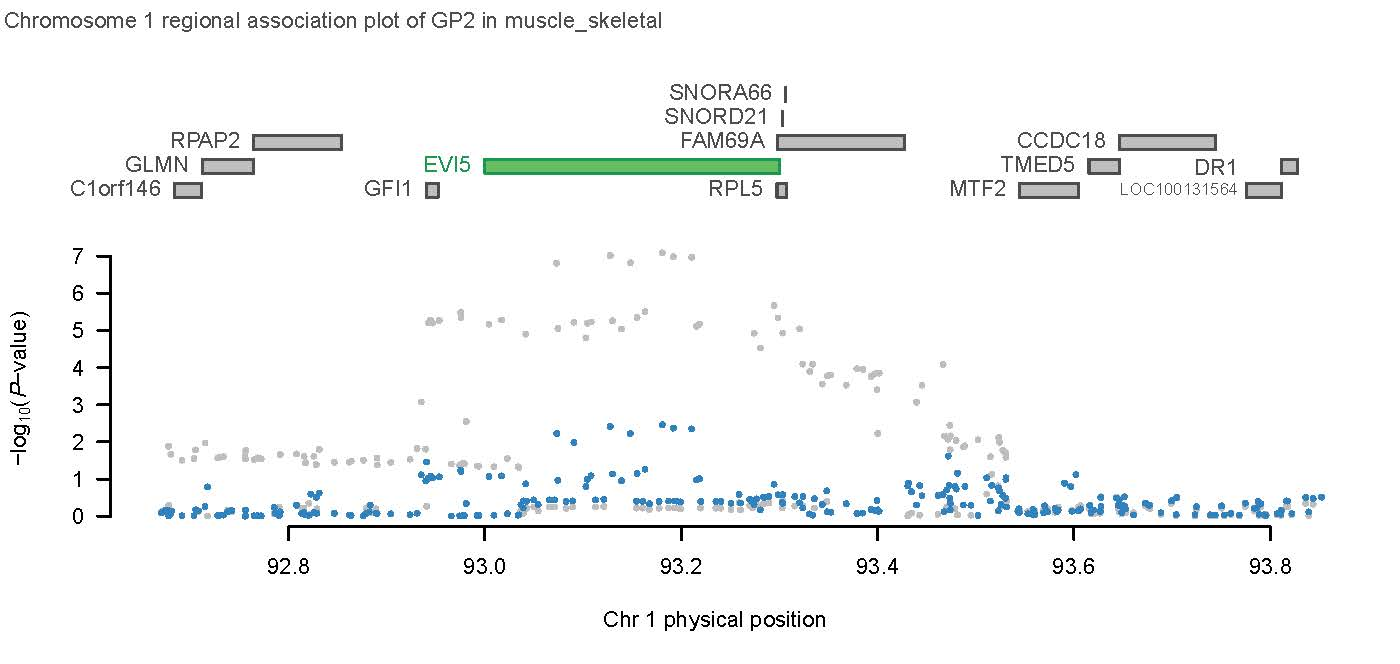


**Supplementary Figure 3.1 |** Regional association of novel TWAS hits. The top panel in each plot highlights all genes in this 1 Mb window. The marginally significant genes identified by TWAS are coloured in orange, and the jointly significant genes are highlighted in green. The bottom panel shows a Manhattan plot of the GWAS data before (grey) and after (blue) conditioning on the predicted expression of the green genes.


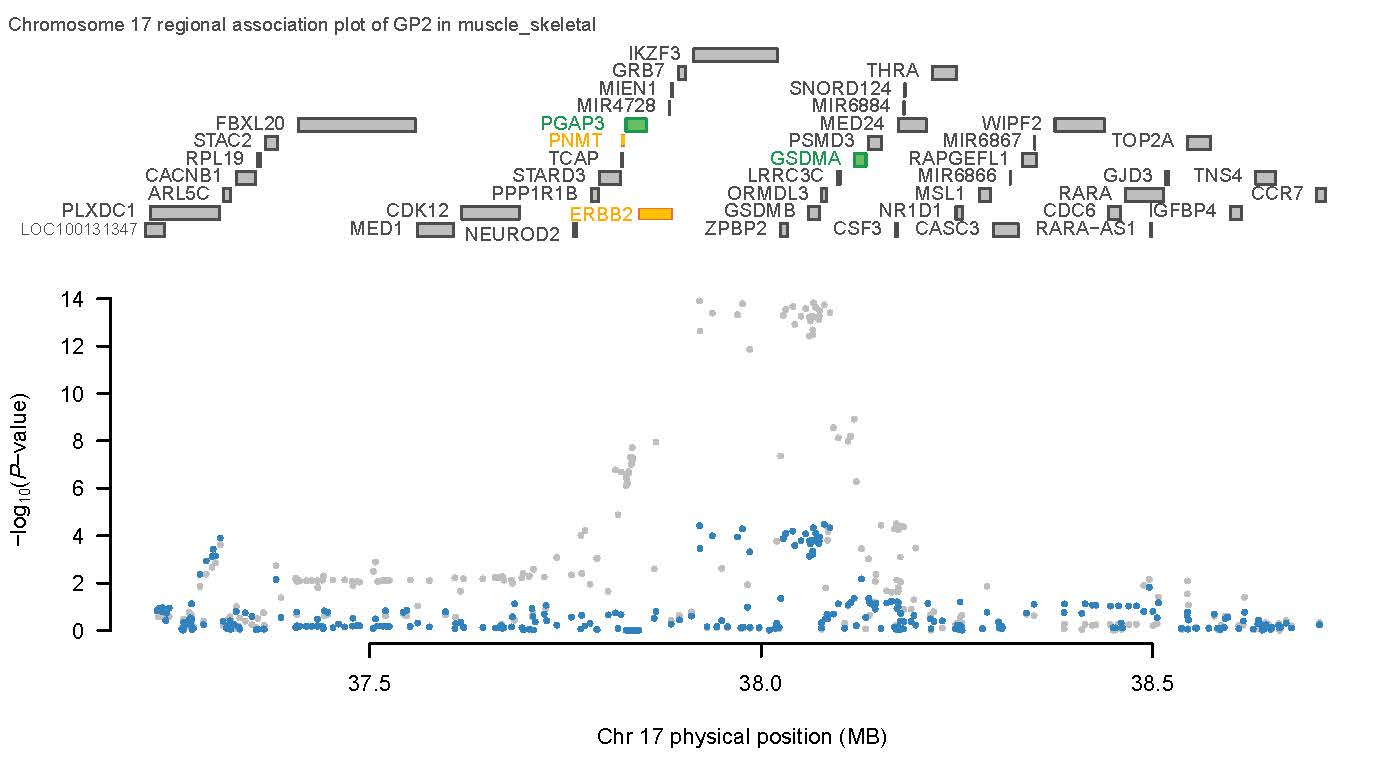


**Supplementary Figure 3.2 |** Regional association of novel TWAS hits. The top panel in each plot highlights all genes in this 1 Mb window. The marginally significant genes identified by TWAS are coloured in orange, and the jointly significant genes are highlighted in green. The bottom panel shows a Manhattan plot of the GWAS data before (grey) and after (blue) conditioning on the predicted expression of the green genes.


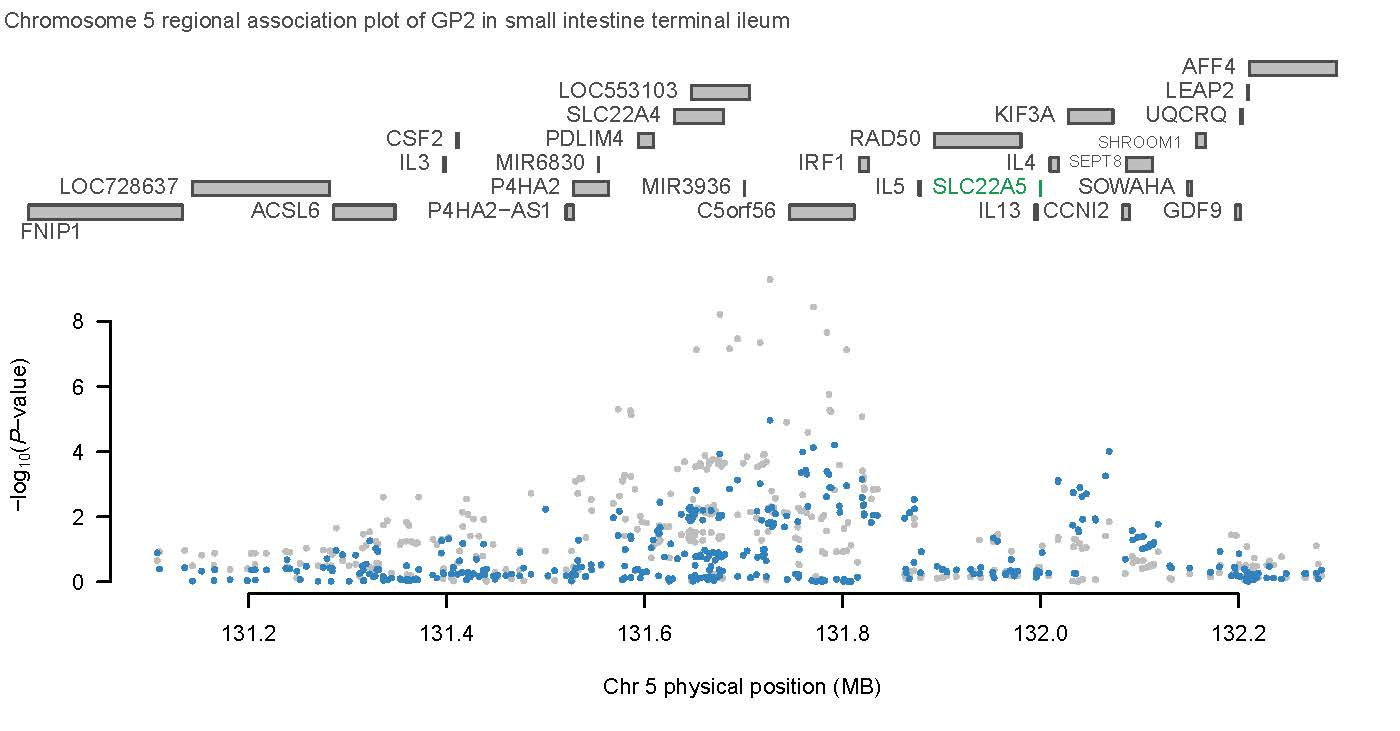


**Supplementary Figure 3.3 |** Regional association of novel TWAS hits. The top panel in each plot highlights all genes in this 1 Mb window. The marginally significant genes identified by TWAS are coloured in orange, and the jointly significant genes are highlighted in green. The bottom panel shows a Manhattan plot of the GWAS data before (grey) and after (blue) conditioning on the predicted expression of the green genes.


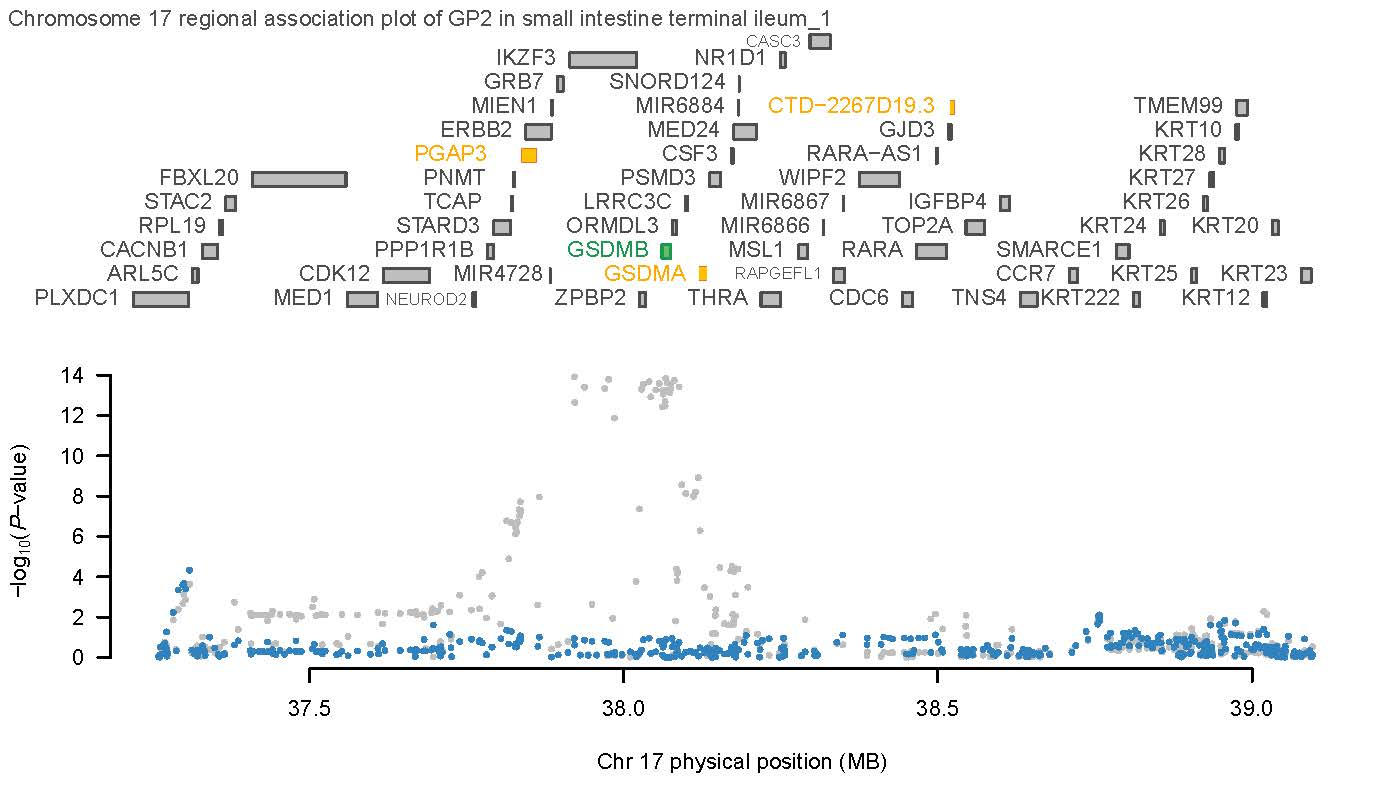


**Supplementary Figure 3.4 |** Regional association of novel TWAS hits. The top panel in each plot highlights all genes in this 1 Mb window. The marginally significant genes identified by TWAS are coloured in orange, and the jointly significant genes are highlighted in green. The bottom panel shows a Manhattan plot of the GWAS data before (grey) and after (blue) conditioning on the predicted expression of the green genes.


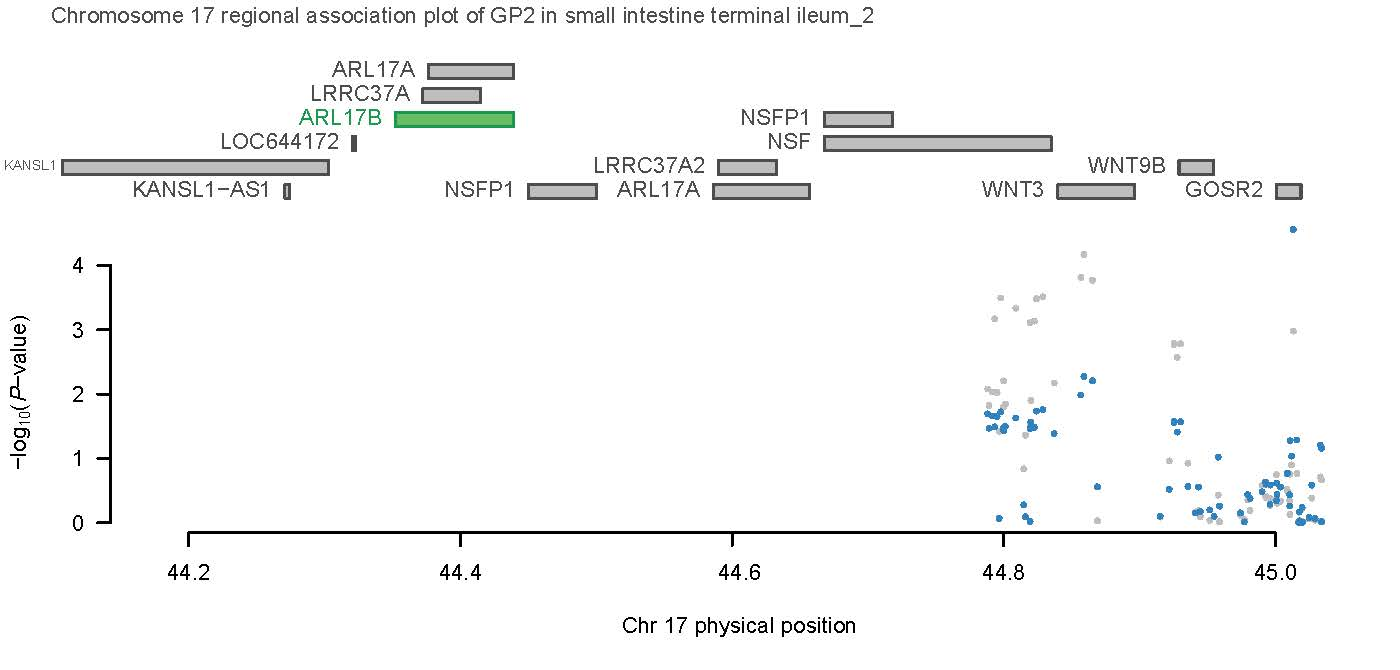


**Supplementary Figure 3.5 |** Regional association of novel TWAS hits. The top panel in each plot highlights all genes in this 1 Mb window. The marginally significant genes identified by TWAS are coloured in orange, and the jointly significant genes are highlighted in green. The bottom panel shows a Manhattan plot of the GWAS data before (grey) and after (blue) conditioning on the predicted expression of the green genes.


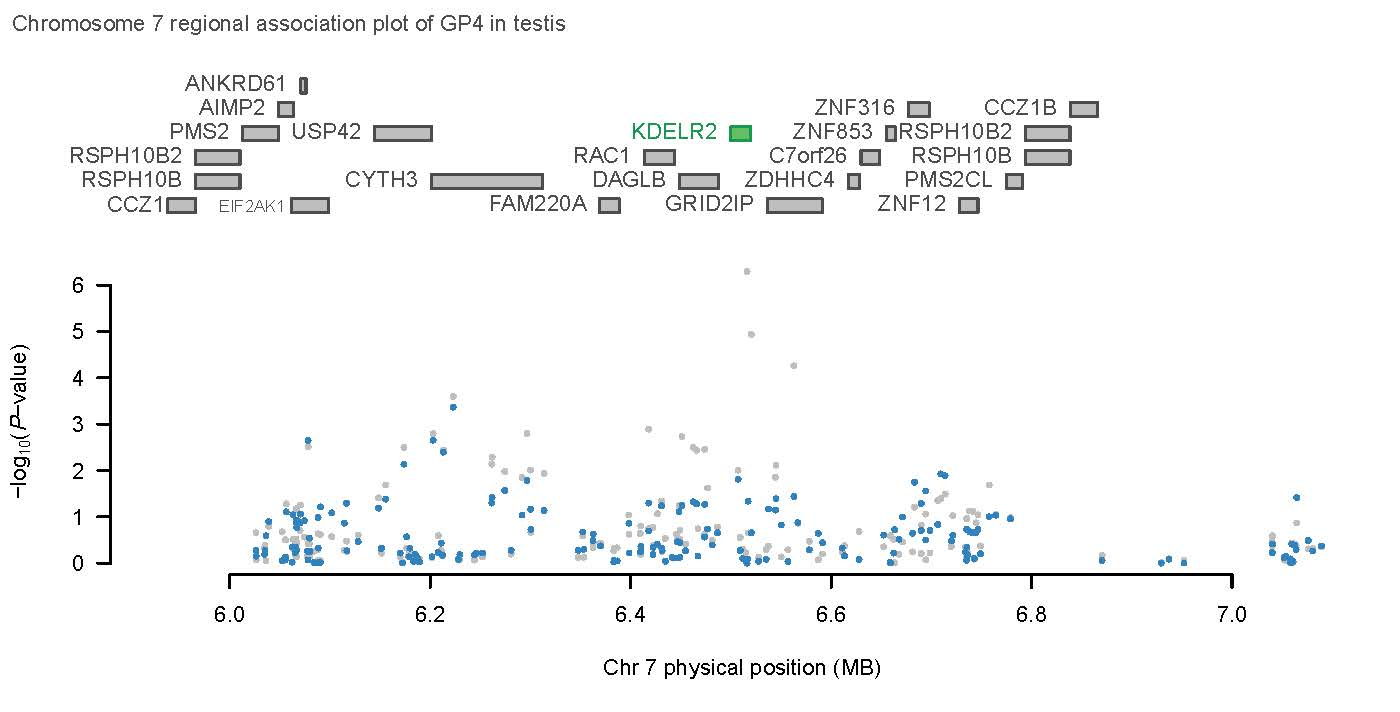


**Supplementary Figure 3.6 |** Regional association of novel TWAS hits. The top panel in each plot highlights all genes in this 1 Mb window. The marginally significant genes identified by TWAS are coloured in orange, and the jointly significant genes are highlighted in green. The bottom panel shows a Manhattan plot of the GWAS data before (grey) and after (blue) conditioning on the predicted expression of the green genes.


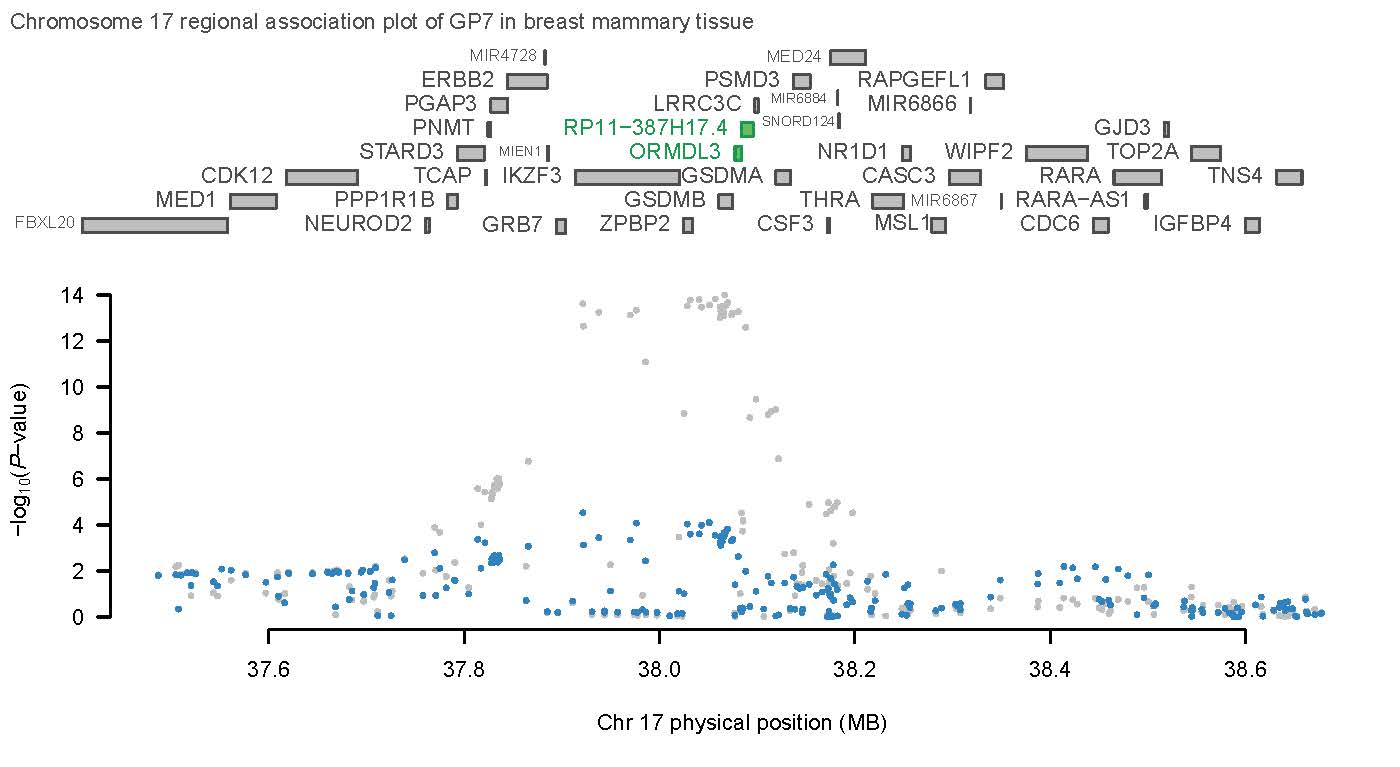


**Supplementary Figure 3.7 |** Regional association of novel TWAS hits. The top panel in each plot highlights all genes in this 1 Mb window. The marginally significant genes identified by TWAS are coloured in orange, and the jointly significant genes are highlighted in green. The bottom panel shows a Manhattan plot of the GWAS data before (grey) and after (blue) conditioning on the predicted expression of the green genes.


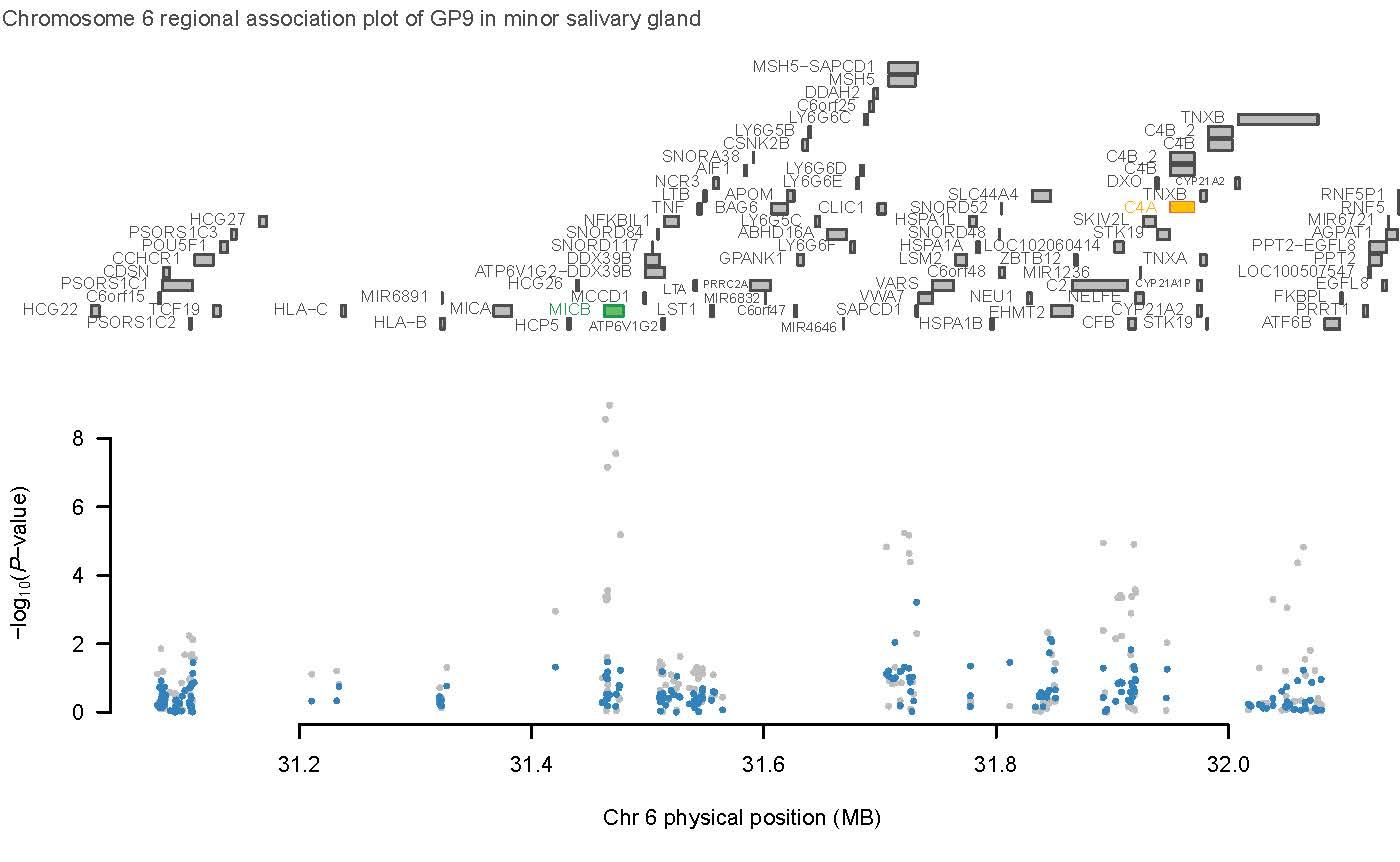


**Supplementary Figure 3.8 |** Regional association of novel TWAS hits. The top panel in each plot highlights all genes in this 1 Mb window. The marginally significant genes identified by TWAS are coloured in orange, and the jointly significant genes are highlighted in green. The bottom panel shows a Manhattan plot of the GWAS data before (grey) and after (blue) conditioning on the predicted expression of the green genes.


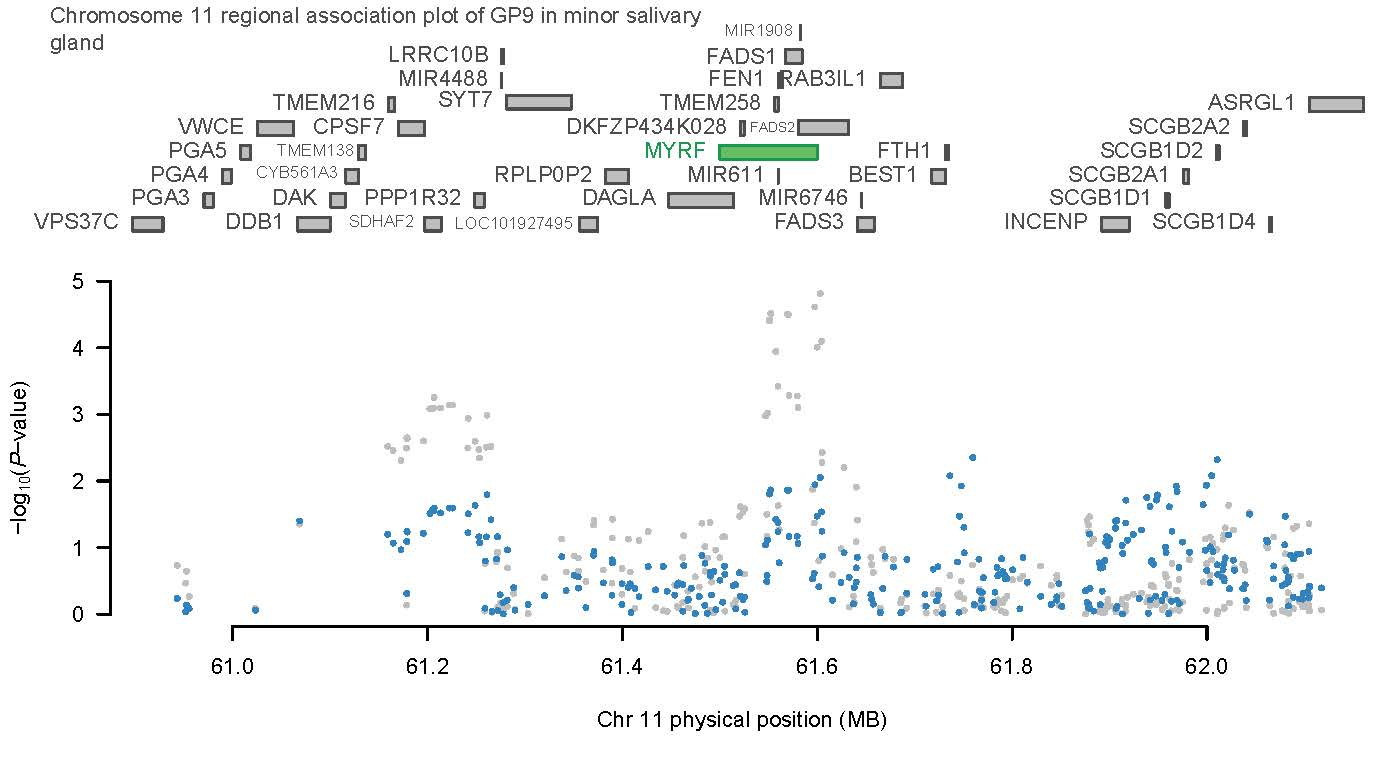


**Supplementary Figure 3.9 |** Regional association of novel TWAS hits. The top panel in each plot highlights all genes in this 1 Mb window. The marginally significant genes identified by TWAS are coloured in orange, and the jointly significant genes are highlighted in green. The bottom panel shows a Manhattan plot of the GWAS data before (grey) and after (blue) conditioning on the predicted expression of the green genes.


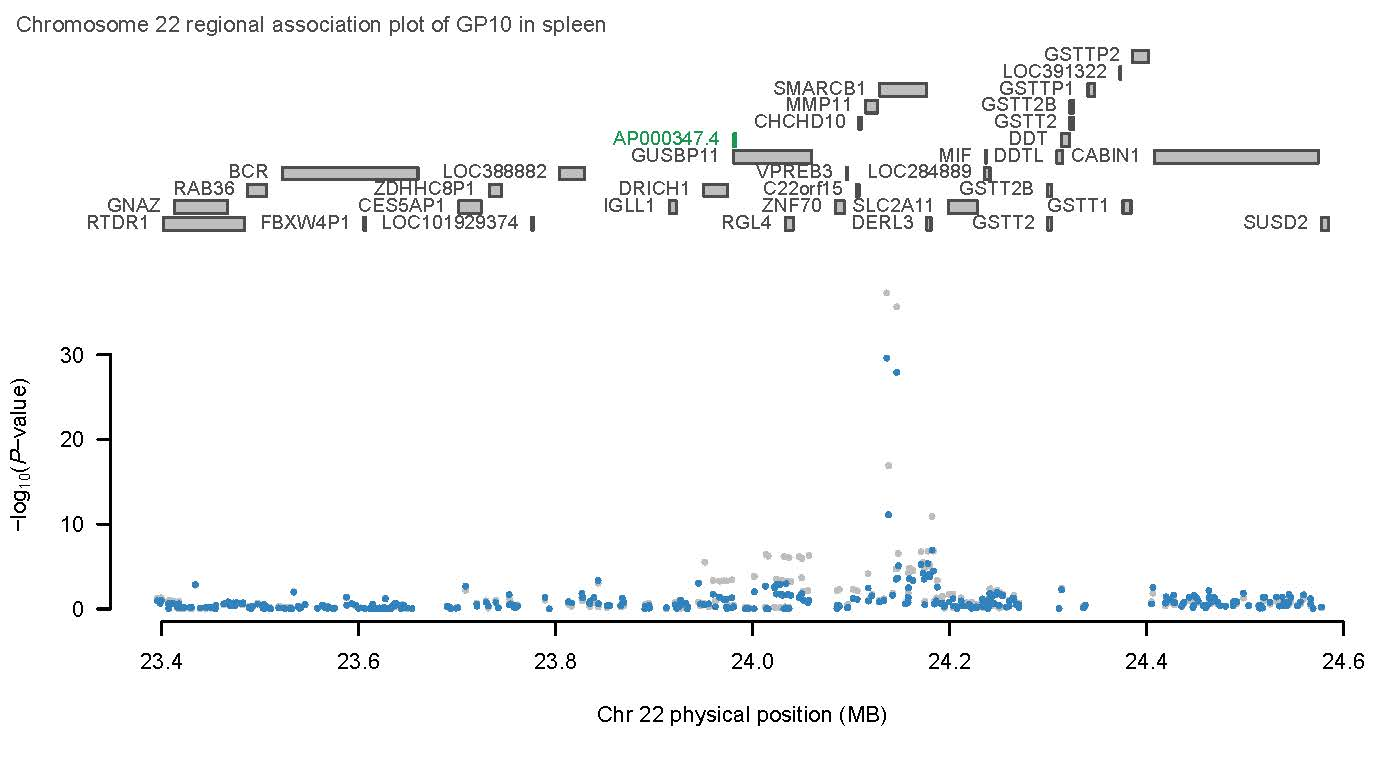


**Supplementary Figure 3.10 |** Regional association of novel TWAS hits. The top panel in each plot highlights all genes in this 1 Mb window. The marginally significant genes identified by TWAS are coloured in orange, and the jointly significant genes are highlighted in green. The bottom panel shows a Manhattan plot of the GWAS data before (grey) and after (blue) conditioning on the predicted expression of the green genes.


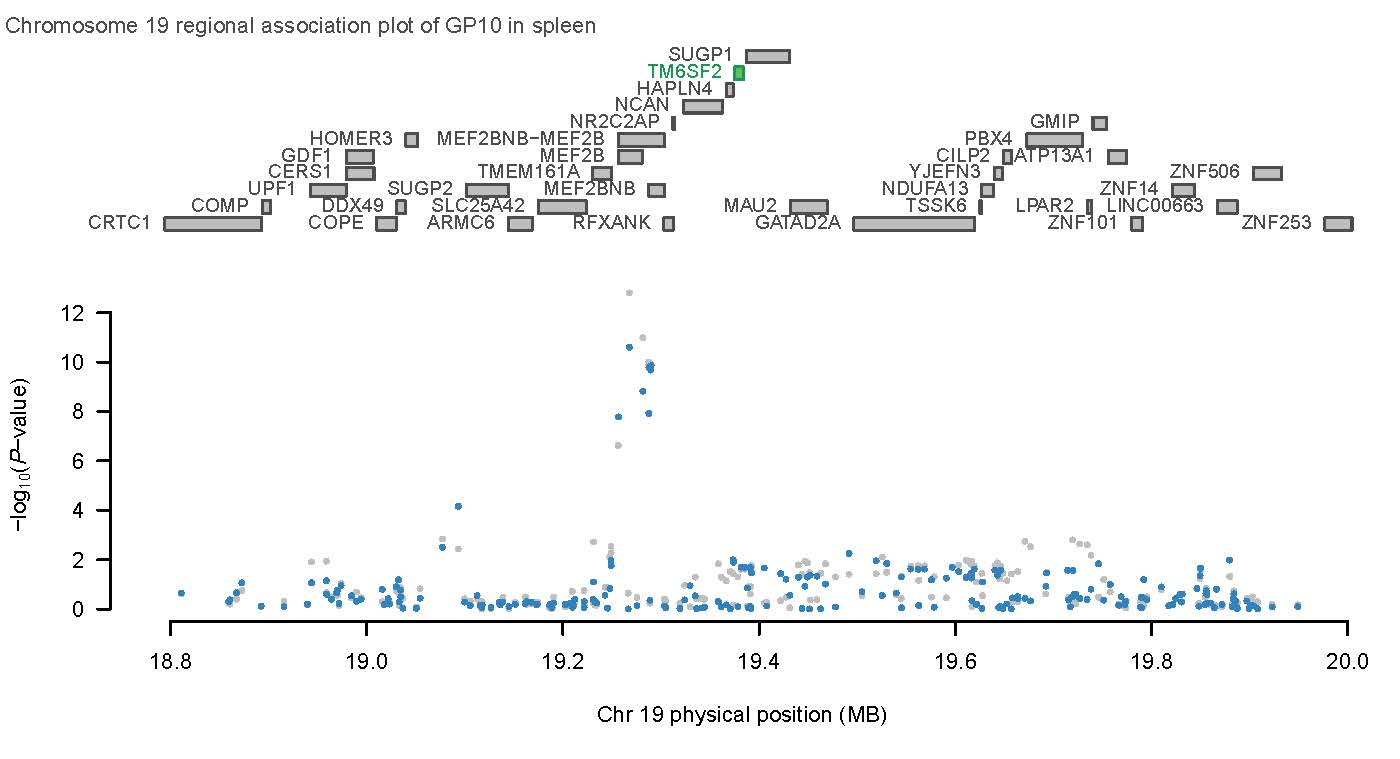


**Supplementary Figure 3.11 |** Regional association of novel TWAS hits. The top panel in each plot highlights all genes in this 1 Mb window. The marginally significant genes identified by TWAS are coloured in orange, and the jointly significant genes are highlighted in green. The bottom panel shows a Manhattan plot of the GWAS data before (grey) and after (blue) conditioning on the predicted expression of the green genes.


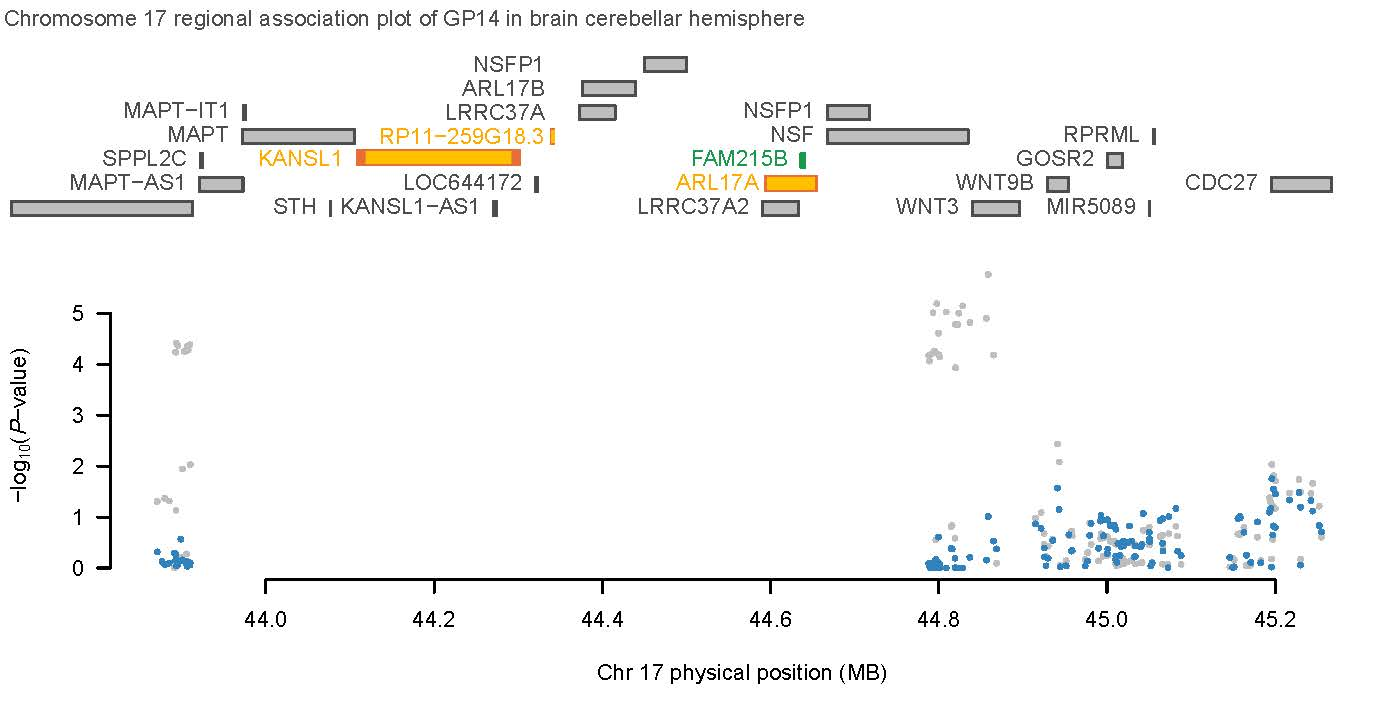


**Supplementary Figure 3.12 |** Regional association of novel TWAS hits. The top panel in each plot highlights all genes in this 1 Mb window. The marginally significant genes identified by TWAS are coloured in orange, and the jointly significant genes are highlighted in green. The bottom panel shows a Manhattan plot of the GWAS data before (grey) and after (blue) conditioning on the predicted expression of the green genes.


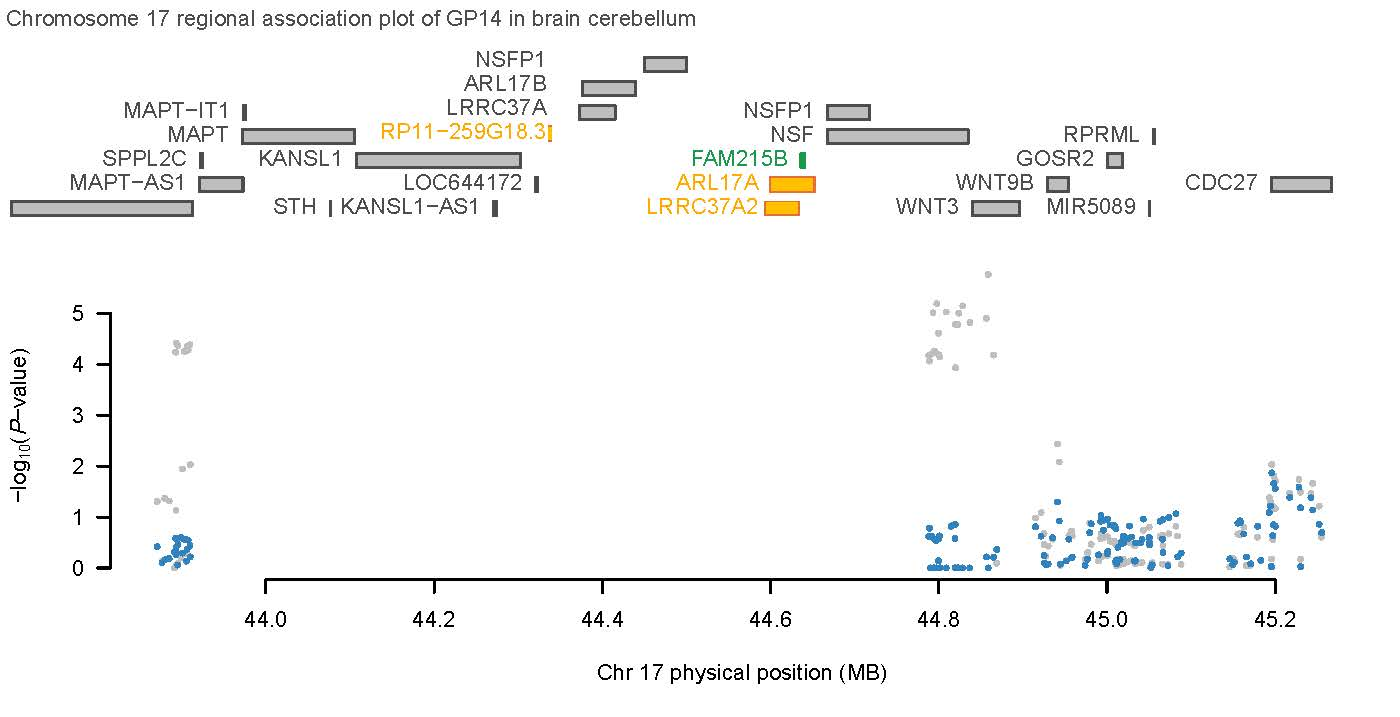


**Supplementary Figure 3.13 |** Regional association of novel TWAS hits. The top panel in each plot highlights all genes in this 1 Mb window. The marginally significant genes identified by TWAS are coloured in orange, and the jointly significant genes are highlighted in green. The bottom panel shows a Manhattan plot of the GWAS data before (grey) and after (blue) conditioning on the predicted expression of the green genes.


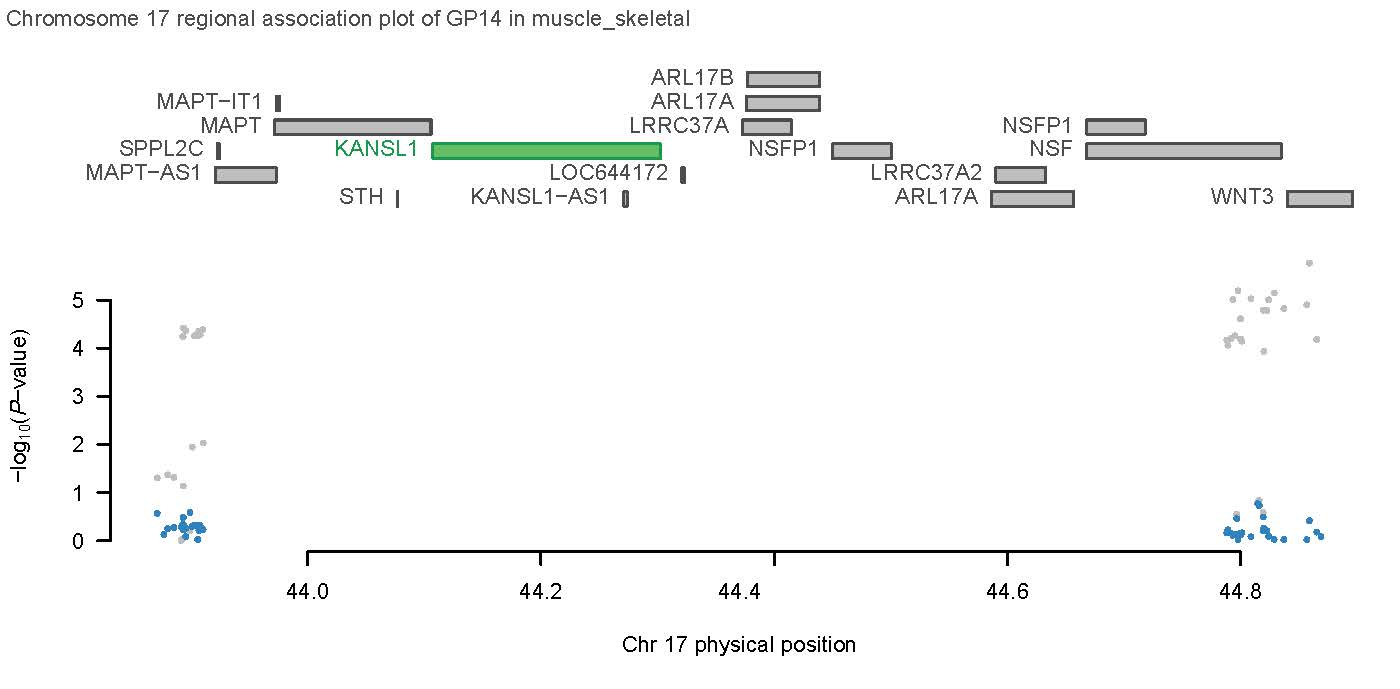


**Supplementary Figure 3.14 |** Regional association of novel TWAS hits. The top panel in each plot highlights all genes in this 1 Mb window. The marginally significant genes identified by TWAS are coloured in orange, and the jointly significant genes are highlighted in green. The bottom panel shows a Manhattan plot of the GWAS data before (grey) and after (blue) conditioning on the predicted expression of the green genes.


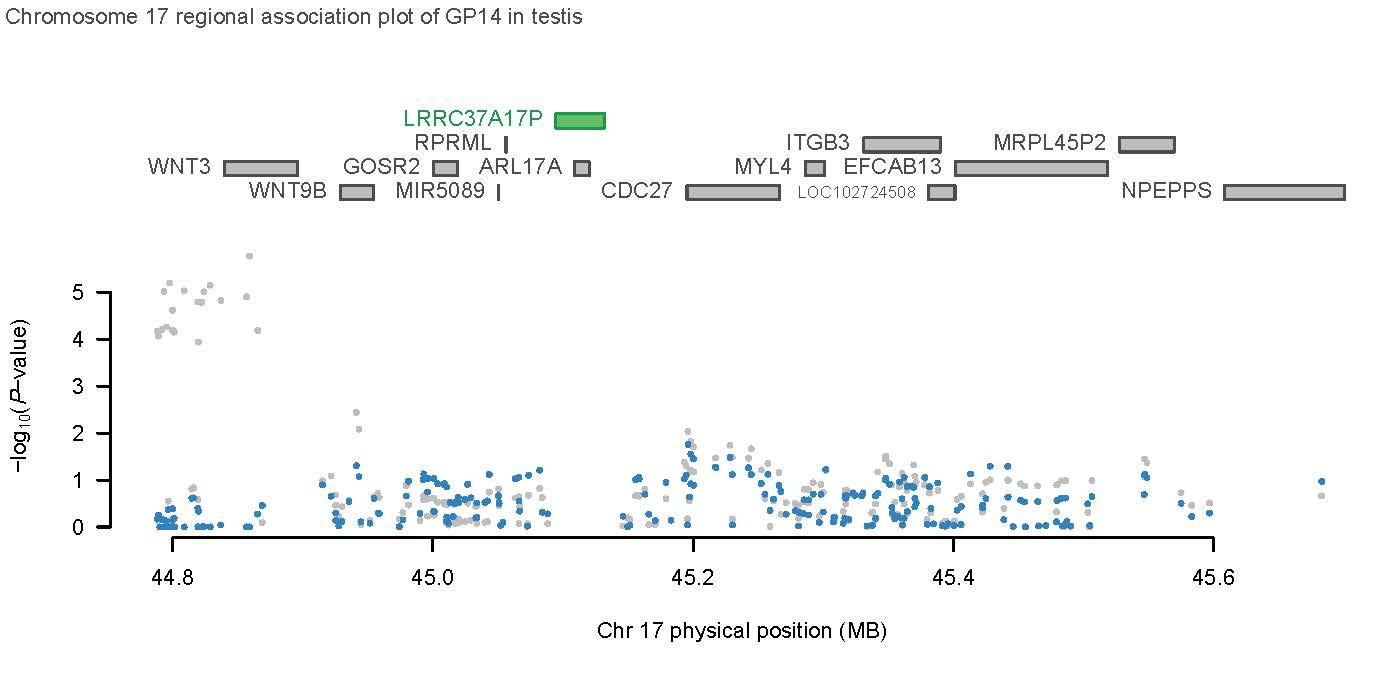


**Supplementary Figure 3.15 |** Regional association of novel TWAS hits. The top panel in each plot highlights all genes in this 1 Mb window. The marginally significant genes identified by TWAS are coloured in orange, and the jointly significant genes are highlighted in green. The bottom panel shows a Manhattan plot of the GWAS data before (grey) and after (blue) conditioning on the predicted expression of the green genes.


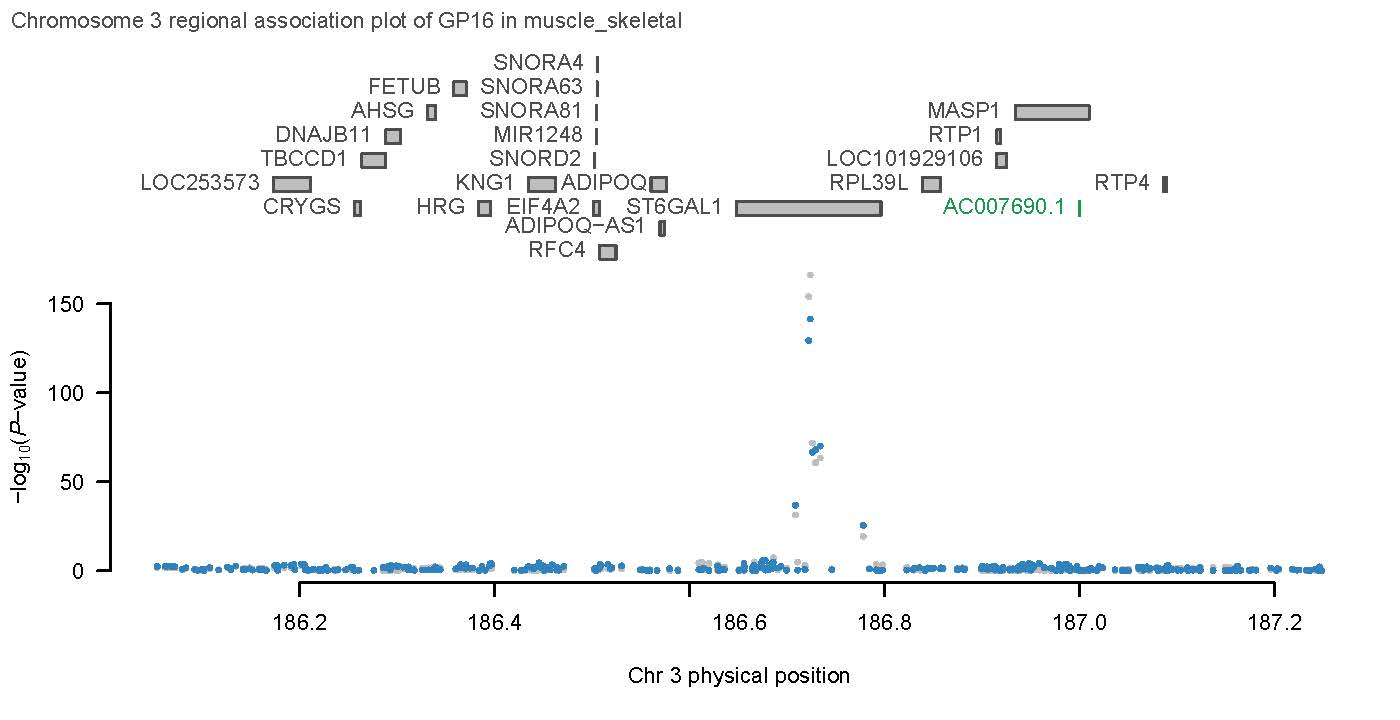


**Supplementary Figure 3.16 |** Regional association of novel TWAS hits. The top panel in each plot highlights all genes in this 1 Mb window. The marginally significant genes identified by TWAS are coloured in orange, and the jointly significant genes are highlighted in green. The bottom panel shows a Manhattan plot of the GWAS data before (grey) and after (blue) conditioning on the predicted expression of the green genes.


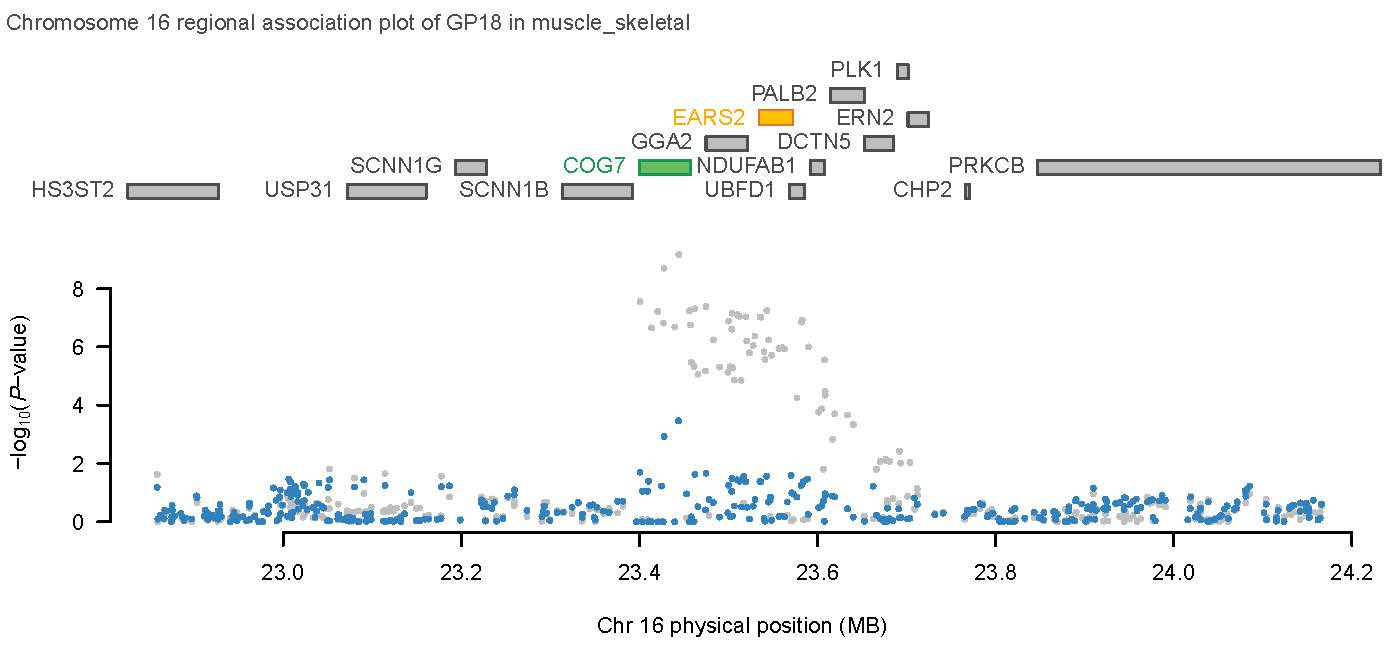


**Supplementary Figure 3.17 |** Regional association of novel TWAS hits. The top panel in each plot highlights all genes in this 1 Mb window. The marginally significant genes identified by TWAS are coloured in orange, and the jointly significant genes are highlighted in green. The bottom panel shows a Manhattan plot of the GWAS data before (grey) and after (blue) conditioning on the predicted expression of the green genes.


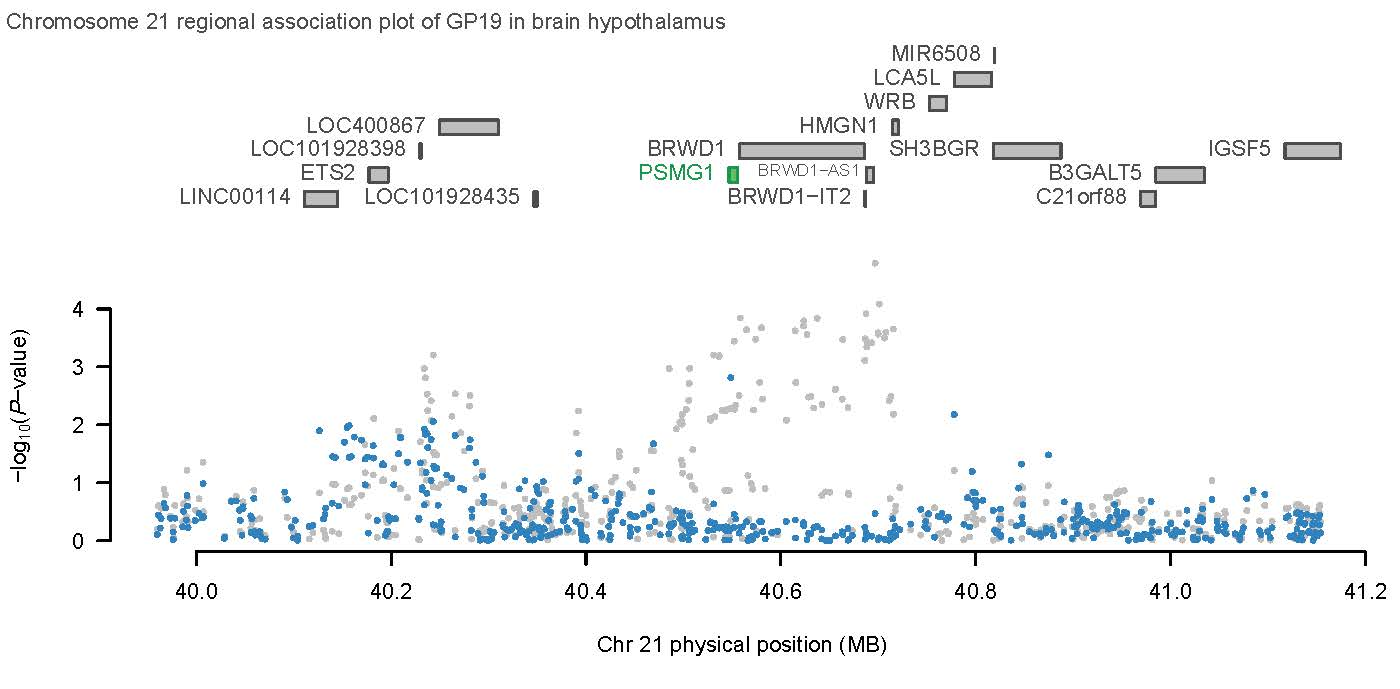


**Supplementary Figure 3.18 |** Regional association of novel TWAS hits. The top panel in each plot highlights all genes in this 1 Mb window. The marginally significant genes identified by TWAS are coloured in orange, and the jointly significant genes are highlighted in green. The bottom panel shows a Manhattan plot of the GWAS data before (grey) and after (blue) conditioning on the predicted expression of the green genes.


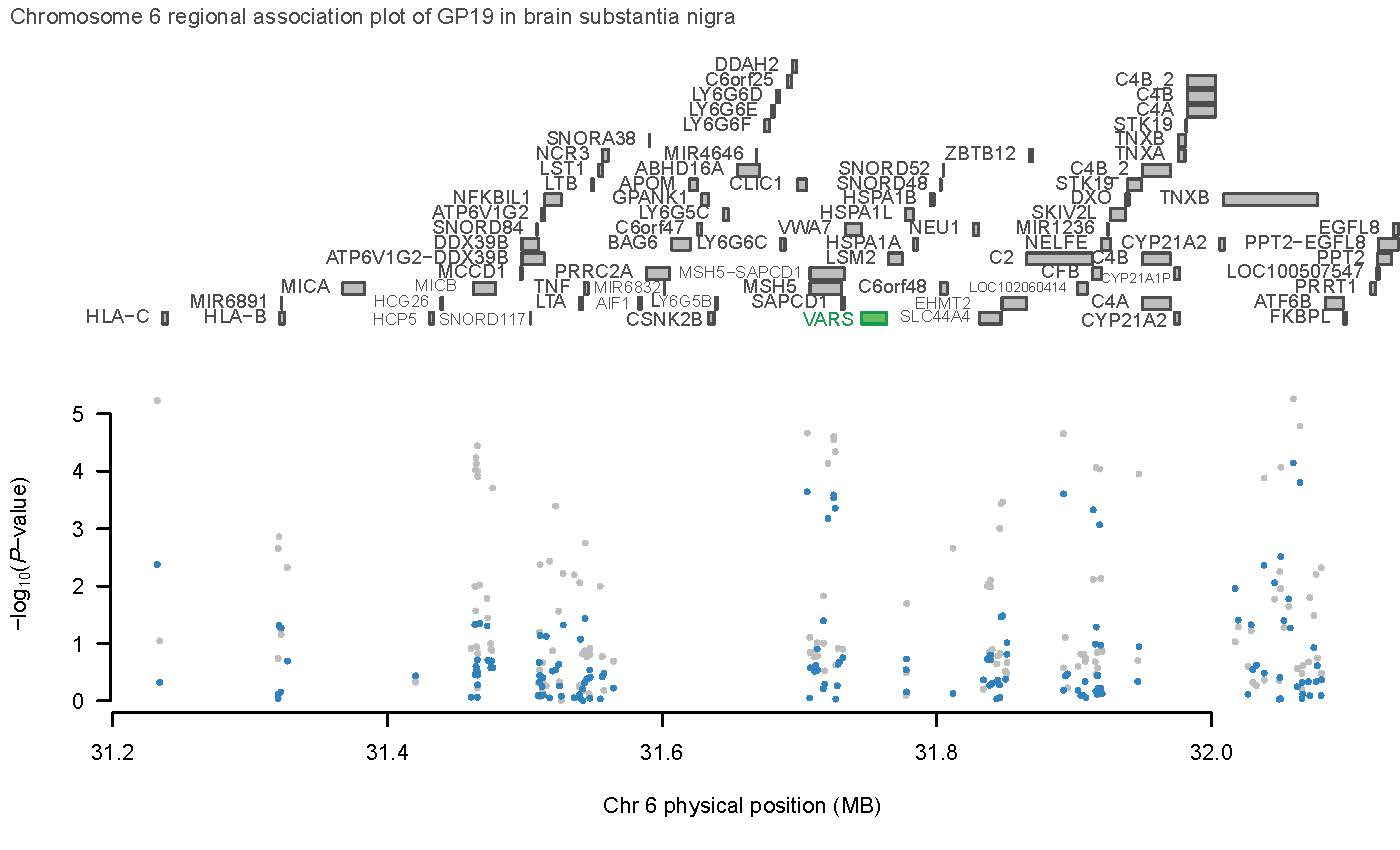


**Supplementary Figure 3.19 |** Regional association of novel TWAS hits. The top panel in each plot highlights all genes in this 1 Mb window. The marginally significant genes identified by TWAS are coloured in orange, and the jointly significant genes are highlighted in green. The bottom panel shows a Manhattan plot of the GWAS data before (grey) and after (blue) conditioning on the predicted expression of the green genes.


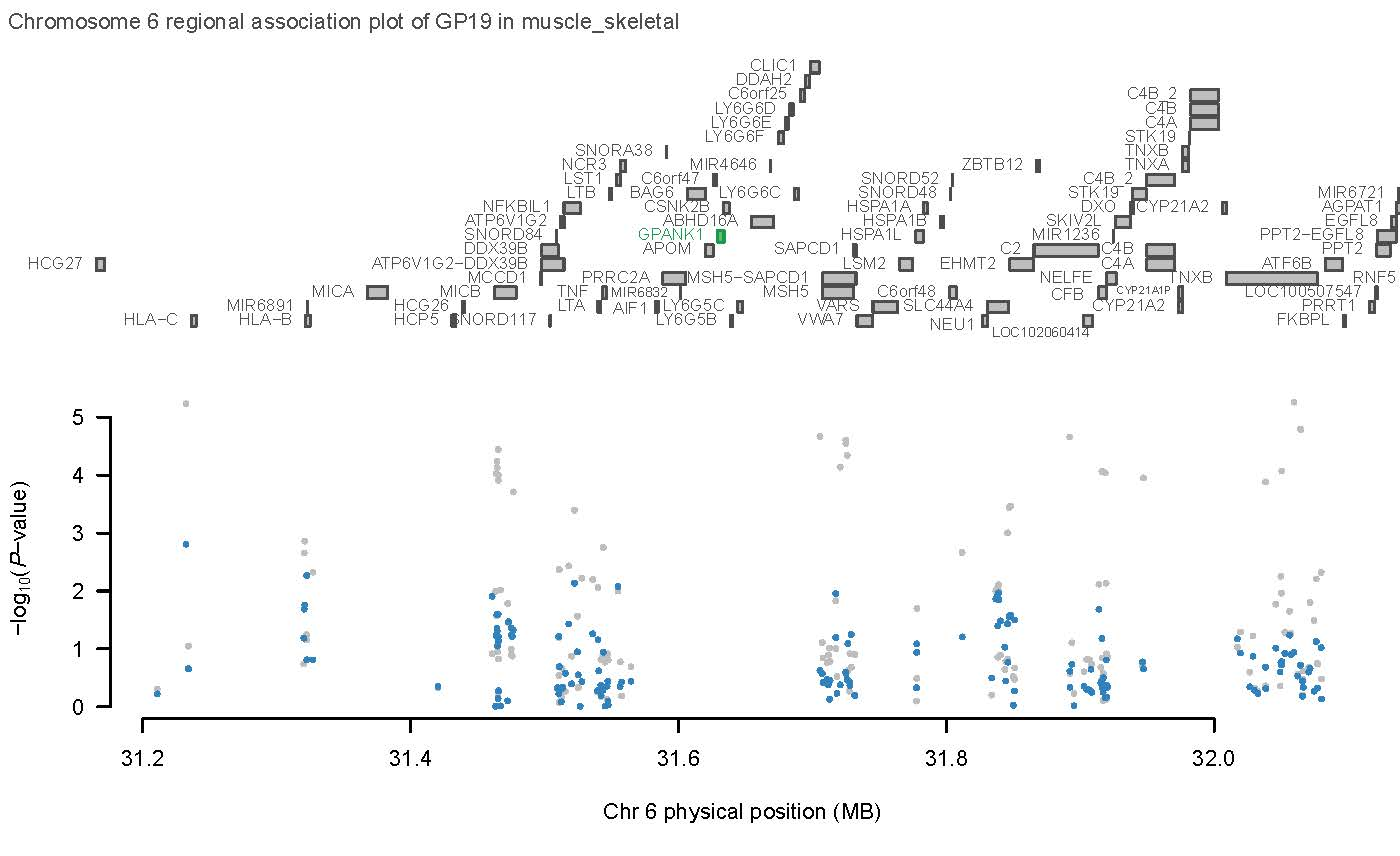


**Supplementary Figure 3.20 |** Regional association of novel TWAS hits. The top panel in each plot highlights all genes in this 1 Mb window. The marginally significant genes identified by TWAS are coloured in orange, and the jointly significant genes are highlighted in green. The bottom panel shows a Manhattan plot of the GWAS data before (grey) and after (blue) conditioning on the predicted expression of the green genes.


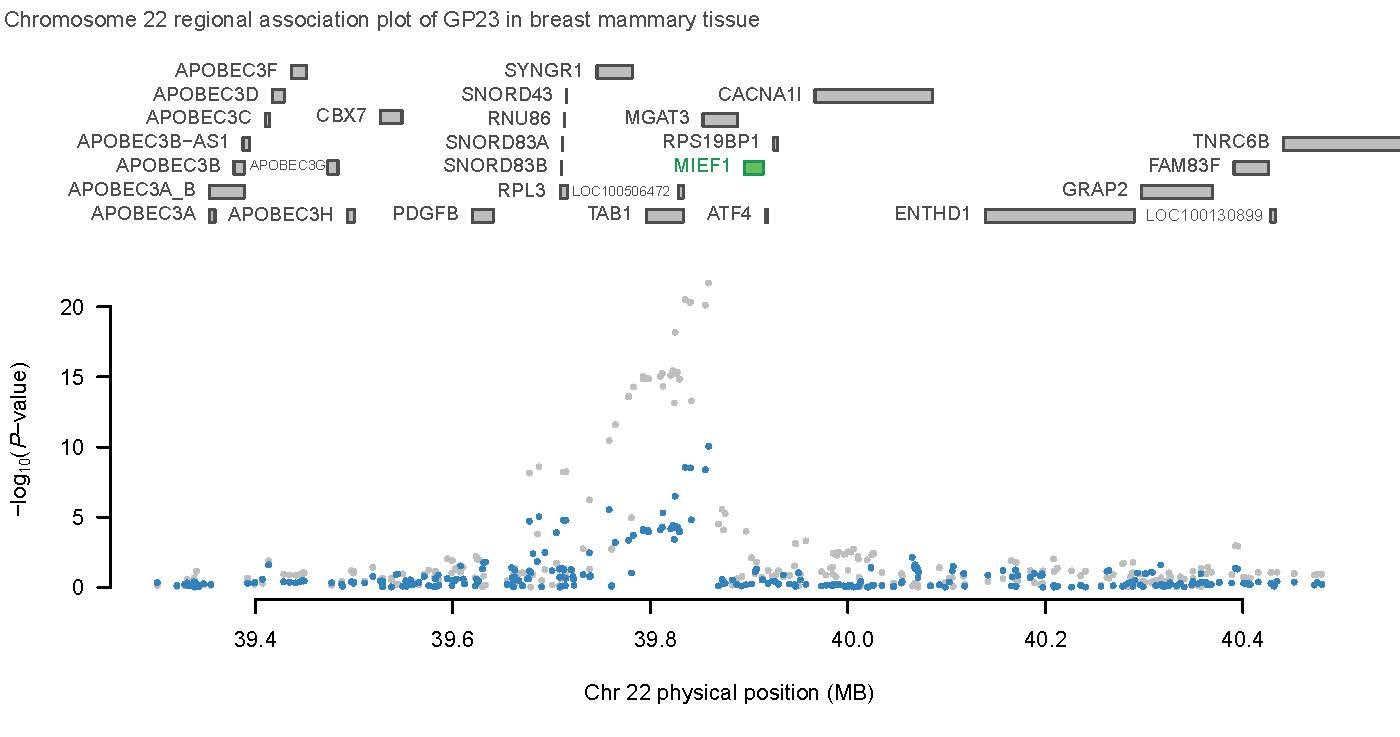


**Supplementary Figure 3.21 |** Regional association of novel TWAS hits. The top panel in each plot highlights all genes in this 1 Mb window. The marginally significant genes identified by TWAS are coloured in orange, and the jointly significant genes are highlighted in green. The bottom panel shows a Manhattan plot of the GWAS data before (grey) and after (blue) conditioning on the predicted expression of the green genes.


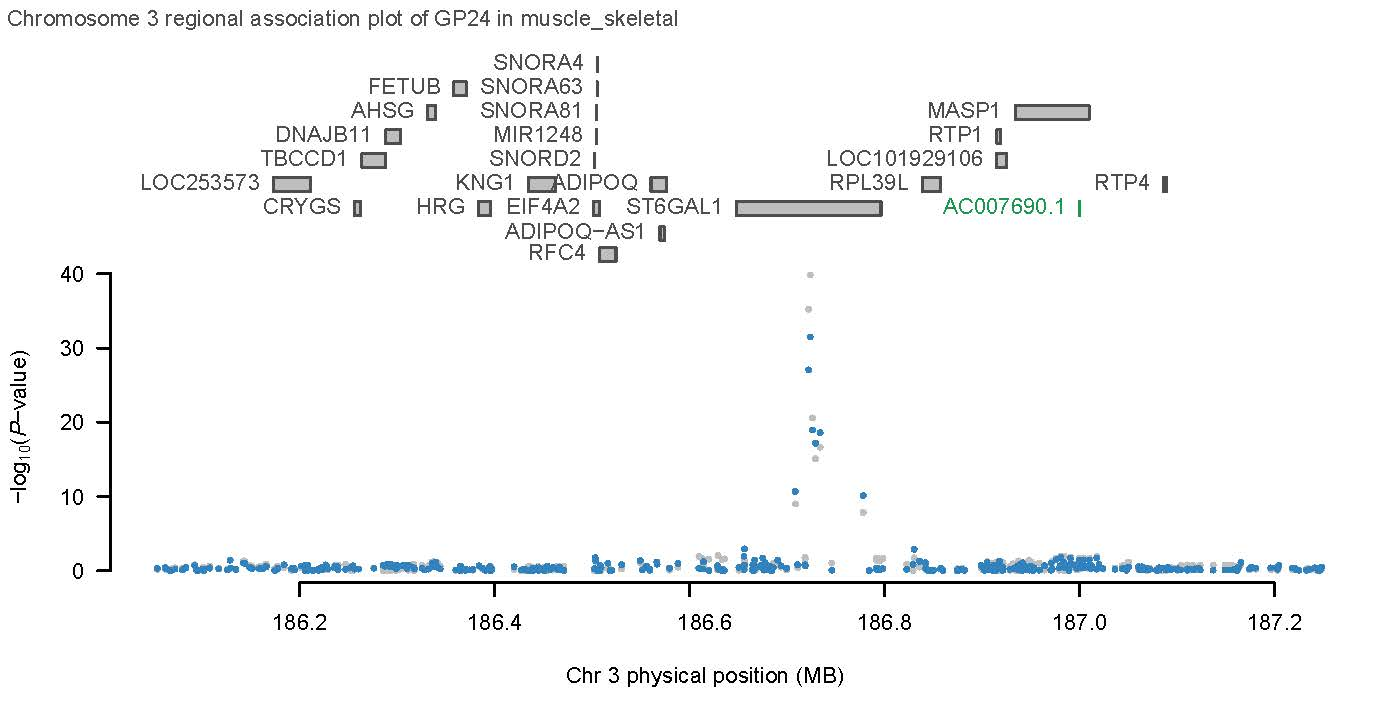


**Supplementary Figure 3.22 |** Regional association of novel TWAS hits. The top panel in each plot highlights all genes in this 1 Mb window. The marginally significant genes identified by TWAS are coloured in orange, and the jointly significant genes are highlighted in green. The bottom panel shows a Manhattan plot of the GWAS data before (grey) and after (blue) conditioning on the predicted expression of the green genes.


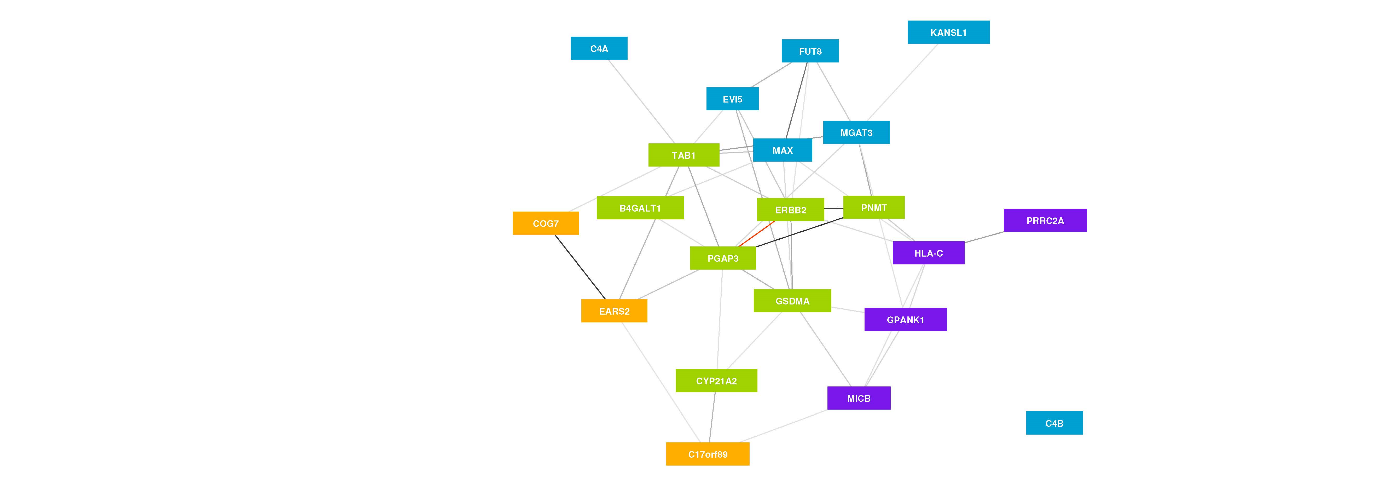


**Supplementary Figure 4.1 |** The co-expression of IgG N-glycosylation related genes in skeletal muscle. Public RNA sequencing data (N = 31,499) was used to identify co-expression patterns. Darker lines suggest a stronger co-expression. Red lines reminder an edge threshold *Z*-score > 13.26.


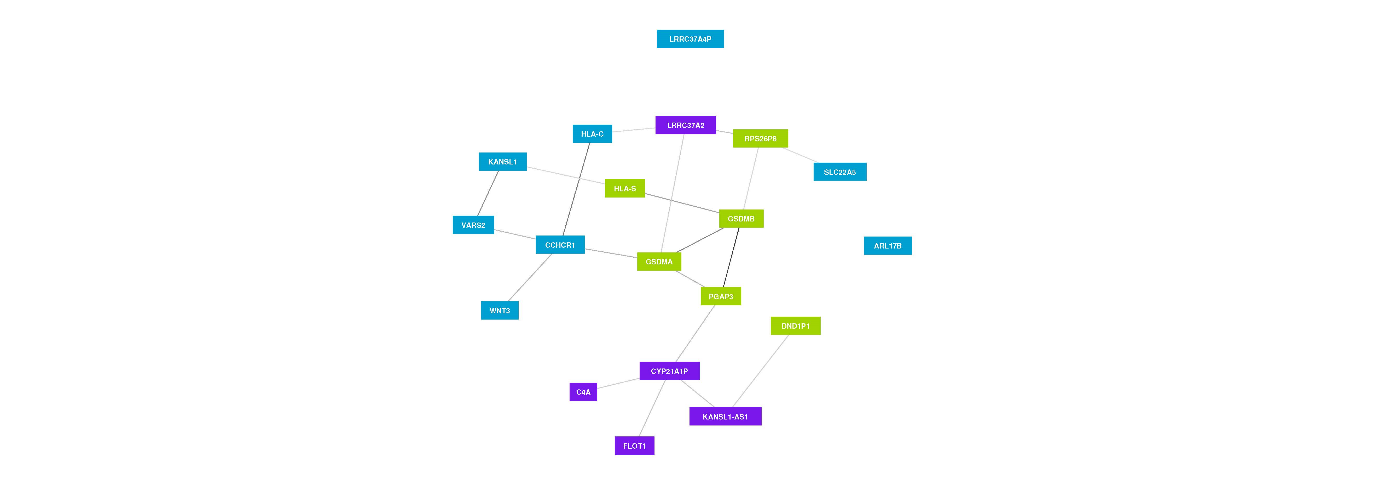


**Supplementary Figure 4.2 |** The co-expression of IgG N-glycosylation related genes in small intestine terminal ileum. Public RNA sequencing data (N = 31,499) was used to identify co-expression patterns. Darker lines suggest a stronger co-expression. Red lines reminder an edge threshold *Z*-score > 13.26.


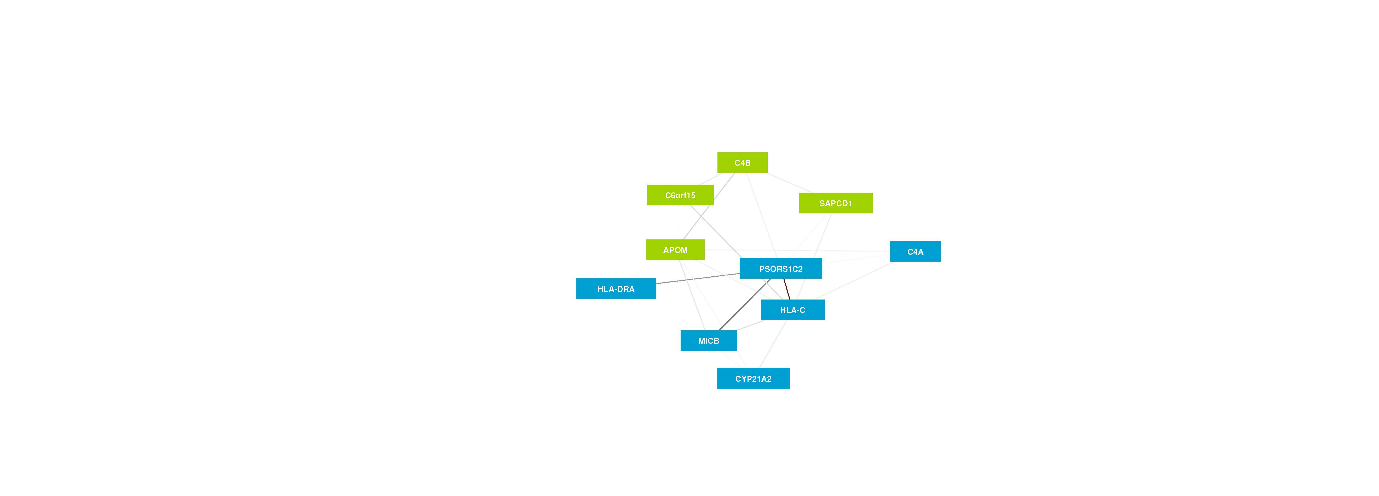


**Supplementary Figure 4.3 |** The co-expression of IgG N-glycosylation related genes in skin sun exposed lower leg. Public RNA sequencing data (N = 31,499) was used to identify co-expression patterns. Darker lines suggest a stronger co-expression. Red lines reminder an edge threshold *Z*-score > 13.26.


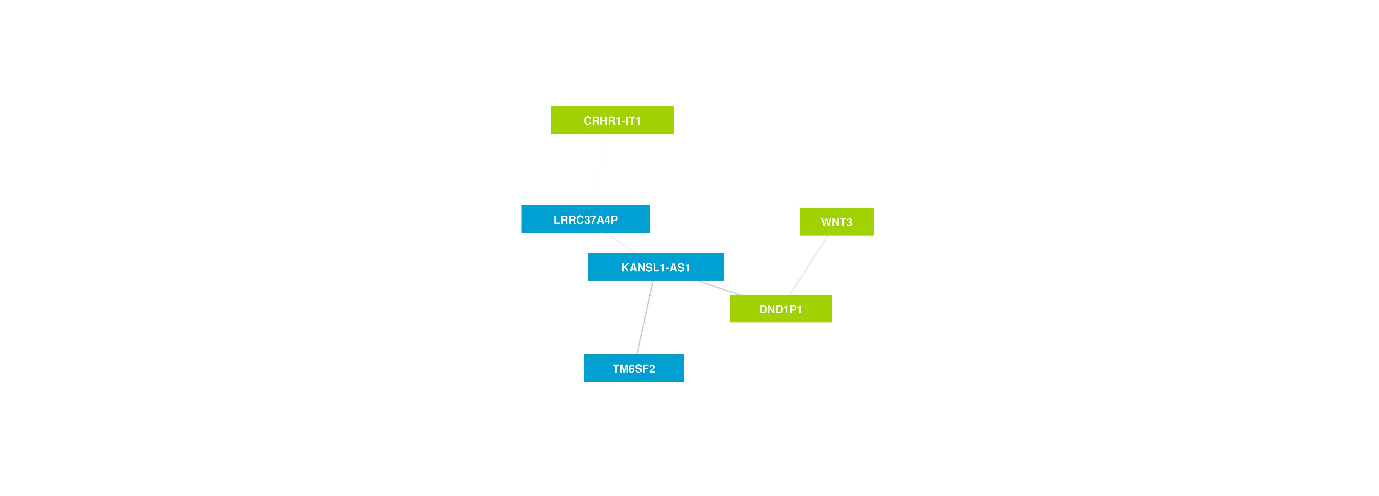


**Supplementary Figure 4.4 |** The co-expression of IgG N-glycosylation related genes in spleen. Public RNA sequencing data (N = 31,499) was used to identify co-expression patterns. Darker lines suggest a stronger co-expression. Red lines reminder an edge threshold *Z*-score > 13.26.


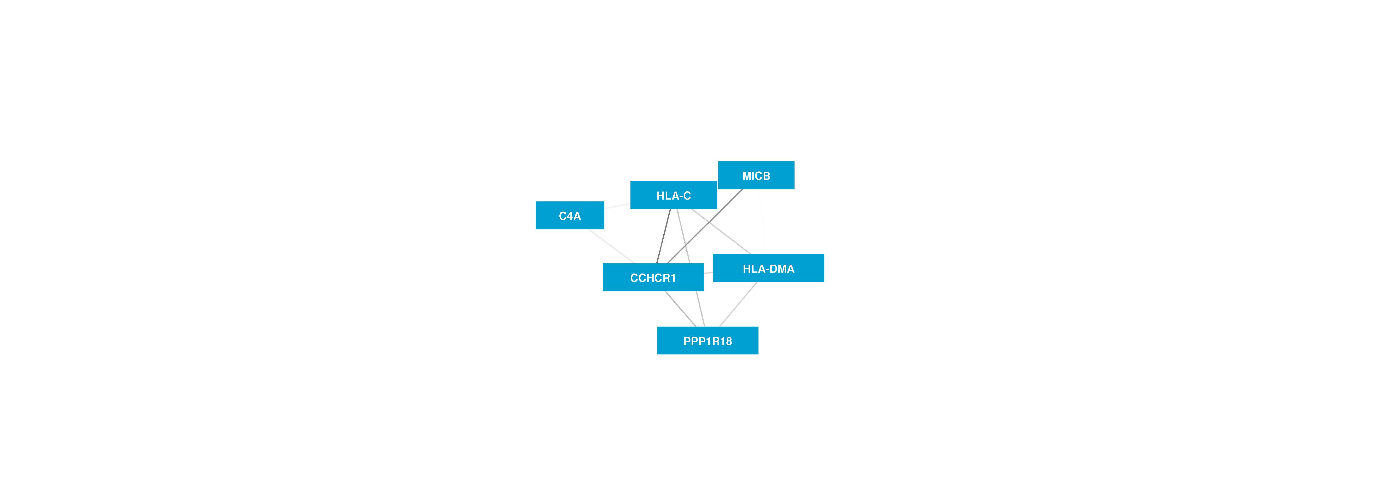


**Supplementary Figure 4.5 |** The co-expression of IgG N-glycosylation related genes in heart atrial appendage. Public RNA sequencing data (N = 31,499) was used to identify co-expression patterns. Darker lines suggest a stronger co-expression. Red lines reminder an edge threshold *Z*-score > 13.26.


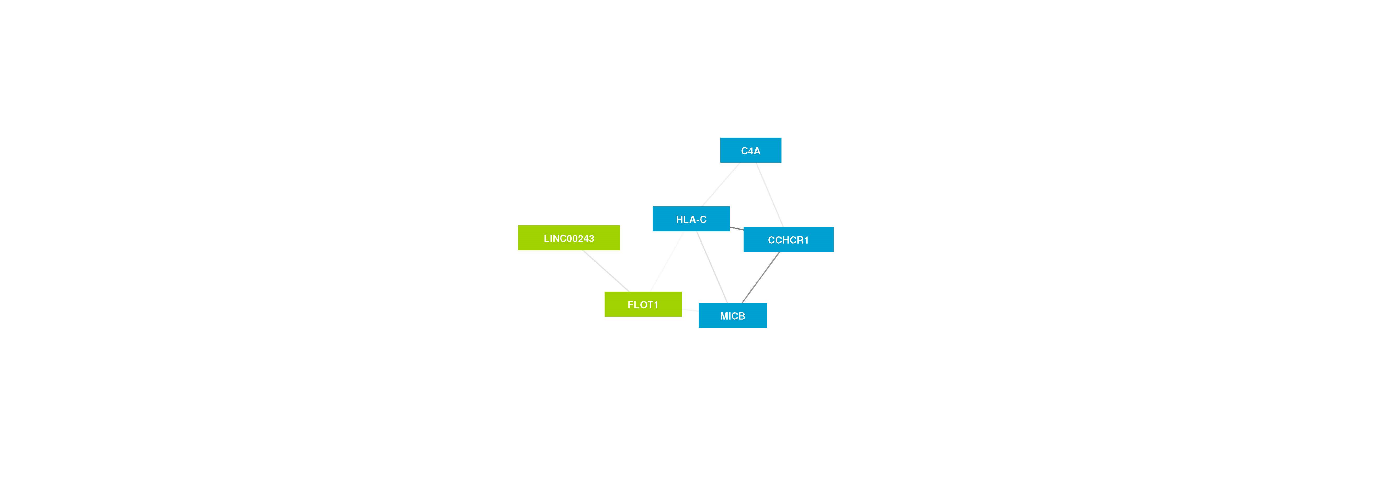


**Supplementary Figure 4.6 |** The co-expression of IgG N-glycosylation related genes in lung. Public RNA sequencing data (N = 31,499) was used to identify co-expression patterns. Darker lines suggest a stronger co-expression. Red lines reminder an edge threshold *Z*-score > 13.26.


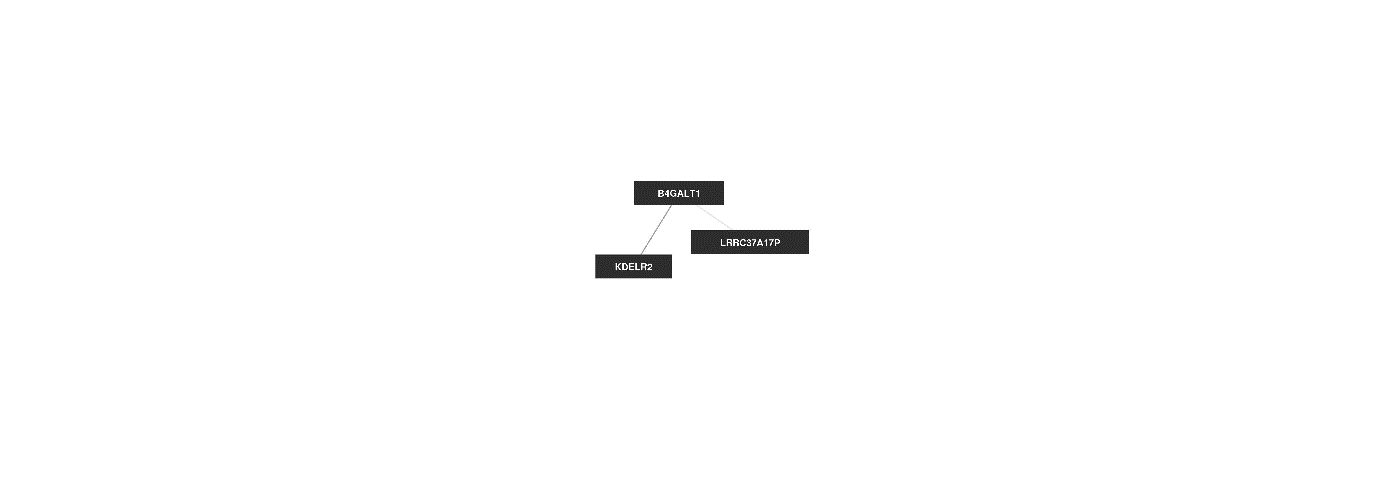


**Supplementary Figure 4.7 |** The co-expression of IgG N-glycosylation related genes in testis. Public RNA sequencing data (N = 31,499) was used to identify co-expression patterns. Darker lines suggest a stronger co-expression. Red lines reminder an edge threshold *Z*-score > 13.26.


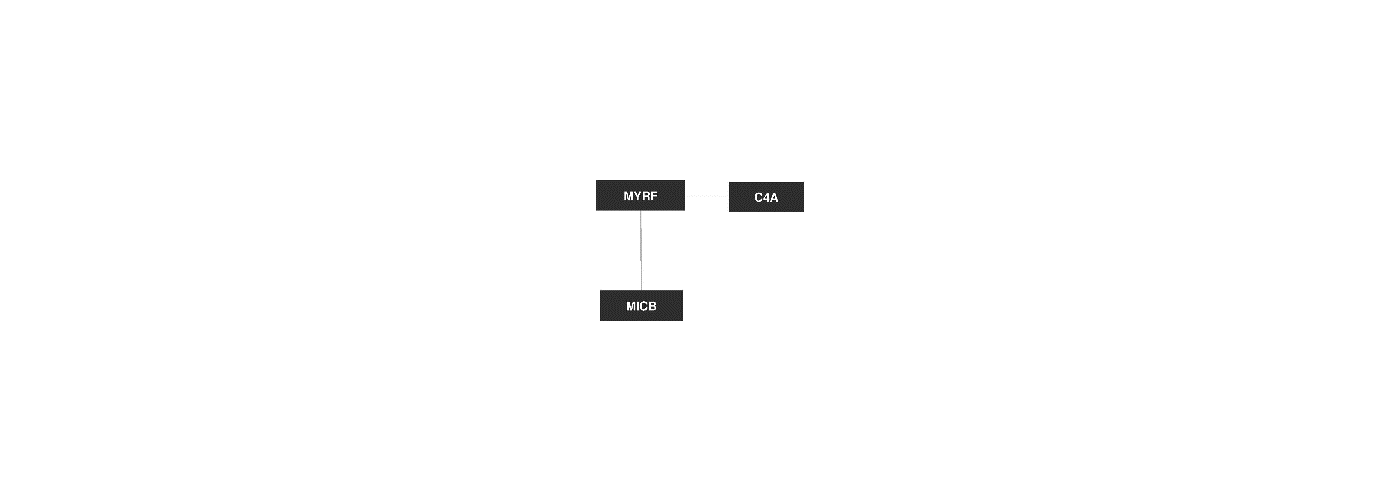


**Supplementary Figure 4.8 |** The co-expression of IgG N-glycosylation related genes in minor salivary gland. Public RNA sequencing data (N = 31,499) was used to identify co-expression patterns. Darker lines suggest a stronger co-expression. Red lines reminder an edge threshold *Z*-score > 13.26.


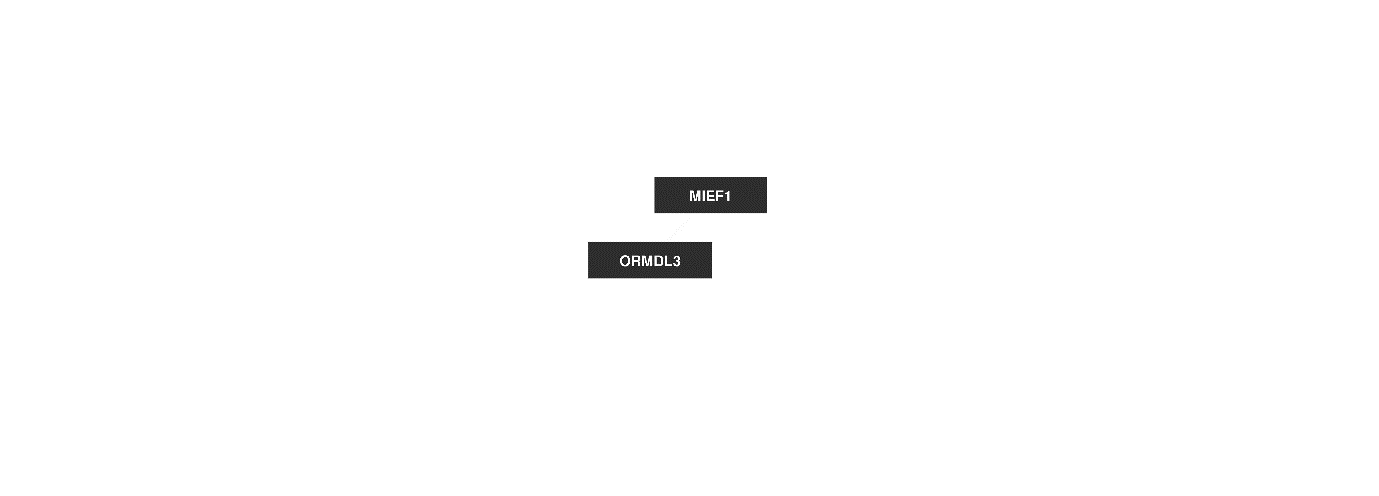


**Supplementary Figure 4.9 |** The co-expression of IgG N-glycosylation related genes in breast mammary tissue. Public RNA sequencing data (N = 31,499) was used to identify co-expression patterns. Darker lines suggest a stronger co-expression. Red lines reminder an edge threshold *Z*-score > 13.26.


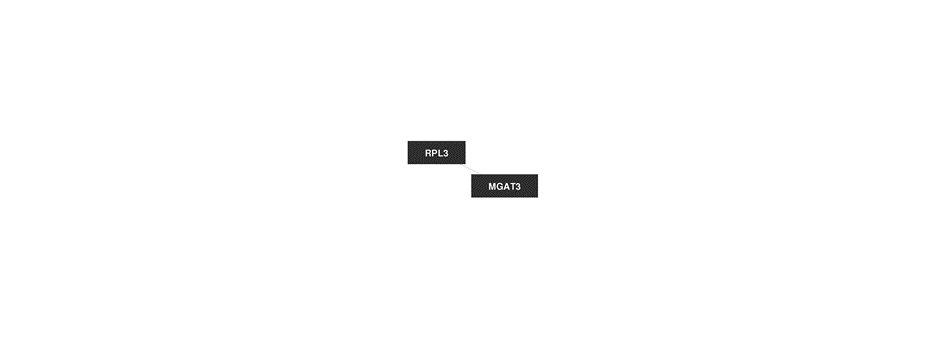


**Supplementary Figure 4.10 |** The co-expression of IgG N-glycosylation related genes in adipose visceral omentum. Public RNA sequencing data (N = 31,499) was used to identify co-expression patterns. Darker lines suggest a stronger co-expression. Red lines reminder an edge threshold *Z*-score > 13.26.


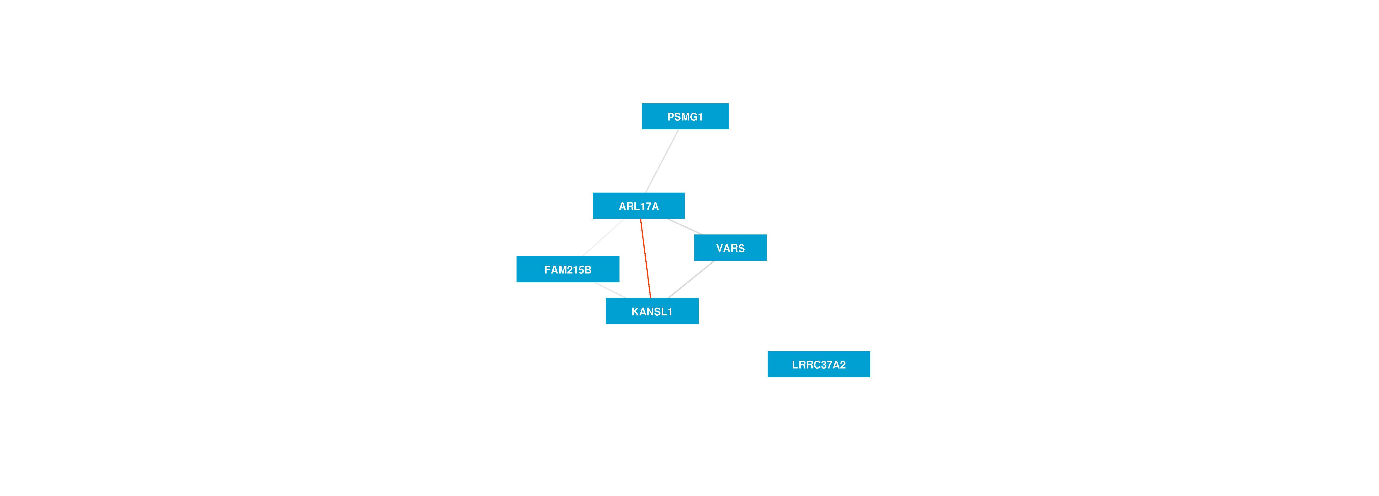


**Supplementary Figure 4.11 |** The co-expression of IgG N-glycosylation related genes in brain. Public RNA sequencing data (N = 31,499) was used to identify co-expression patterns. Darker lines suggest a stronger co-expression. Red lines reminder an edge threshold *Z*-score > 13.26.


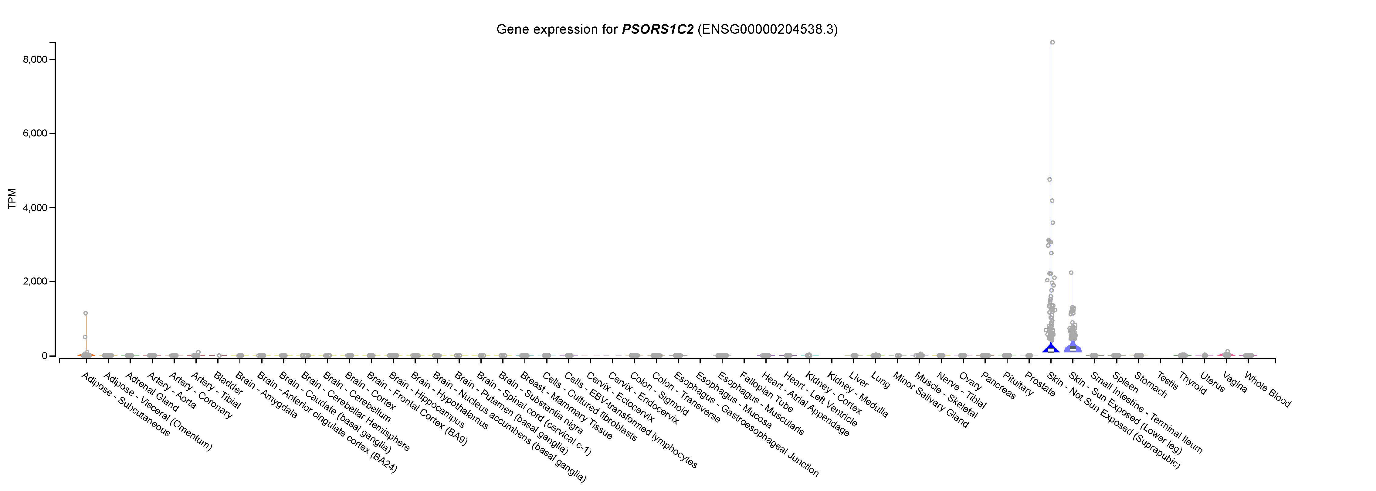


**Supplementary Figure 5** **|** *PSORS1C2* gene exp-plot in GTEx v7. TPM: Transcripts per Million.


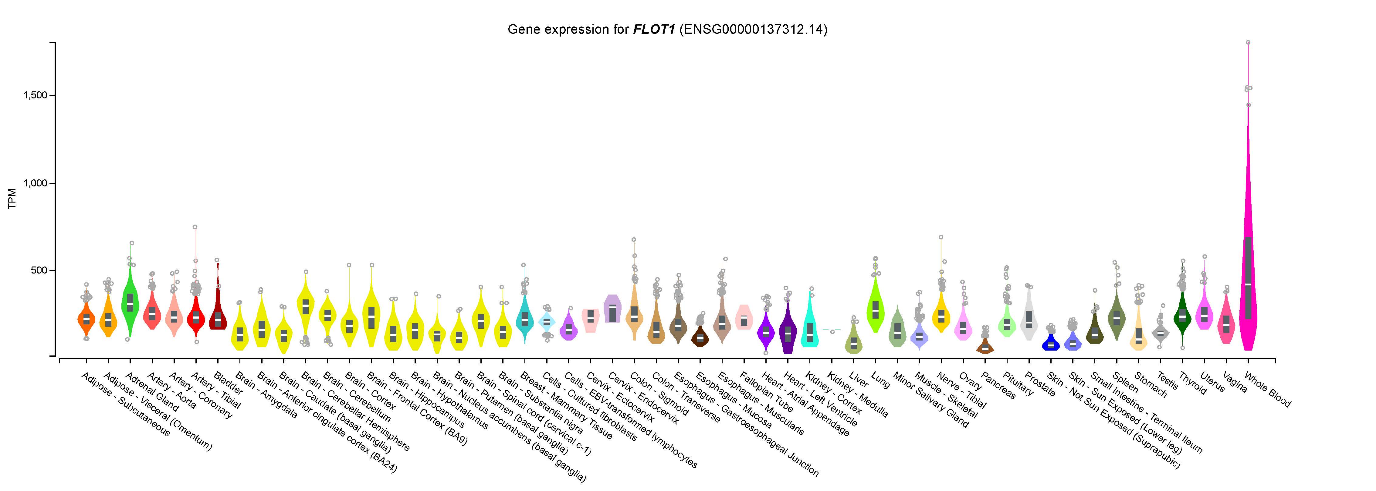


**Supplementary Figure 6 |** *FLOT1* gene exp-plot in GTEx v7. TPM: Transcripts per Million.
